# Synthesis and photoinduced switching properties of C_7_-heteroatom containing push–pull norbornadiene derivatives

**DOI:** 10.3762/bjoc.21.64

**Published:** 2025-04-22

**Authors:** Daniel Krappmann, Andreas Hirsch

**Affiliations:** 1 Department Chemistry and Pharmacy, Friedrich-Alexander-Universität Erlangen-Nürnberg, Nikolaus-Fiebiger Straße 10, 91058 Erlangen, Germany

**Keywords:** heterocycles, molecular solar thermal systems, norbornadiene, photochemistry, quadricyclane

## Abstract

We report the synthesis and characterization of heteroatom-incorporated norbornadiene (NBD) derivatives. Push–pull substitution on the 2 and 3 position as well as introduction of oxygen or nitrogen at position 7 of the NBD scaffold have led to the development of a new family of photoswitches. We studied the potential conversion of norbornadiene to quadricyclane (QC) isomers. As main investigation tools, UV–vis and NMR spectroscopy were utilized. We determined significant spectral features of the formed NBD species, including λ_max_ and λ_onset_ values, all of which exhibit redshifts compared to their isocyclic counterparts. Additionally, the selected QC isomers were subjected to thermal and catalytic back-conversion studies.

## Introduction

**Figure 1:**
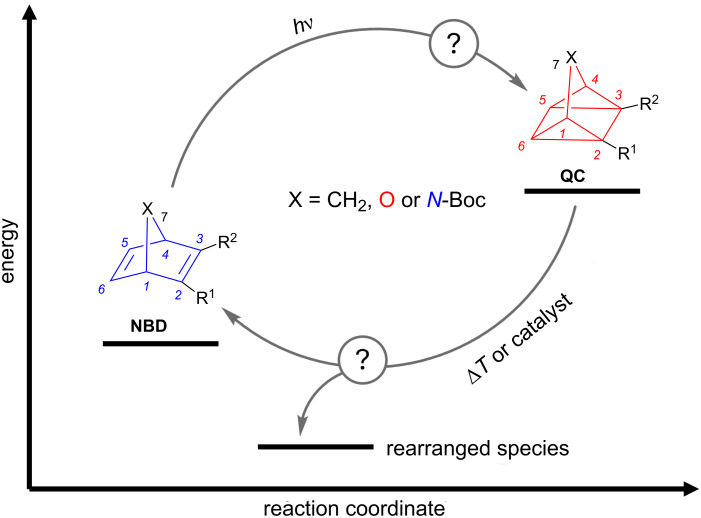
Basic principle of the NBD to QC conversion and vice versa. The bridge-atom at position 7 was varied to carbon, oxygen or nitrogen. The back-conversion induced via heat or a catalyst can result in NBD or in another rearranged species.

In recent decades, the demand for renewable energy has increased tremendously [1]. A promising alternative to fossil fuels is the utilization of so-called molecular solar thermal (MOST) systems [2]. These systems operate on the principle of converting an energy-lean isomer into an energy-rich form by irradiating it with sunlight, effectively storing energy in the form of chemical strain energy [3]. On demand, by application of an external stimulus such as heat, catalysis, or an electrochemical input [4–6], the stored energy can be released converting the molecule back in the parent form (Figure 1). Typical MOST systems are azobenzenes (*E*/*Z*-isomerization) [7–10], the isomerization of dihydroazulenes/vinylheptafulvenes [11–13], conversion of azaborines [14–15] to the corresponding Dewar isomer and the norbornadiene/quadricyclane (NBD/QC) couple [16–18], Notably, the NBD/QC couple exhibits characteristics superior to those of the other mentioned photoswitches, including the highest energy storage density [19]. For MOST systems, it is essential not only to achieve a high storage density and good quantum efficiency for the isomerization process but also to ensure large half-lifes and durability in switching reliability [20]. By making structural adjustments to the parent carbon scaffold, it is possible to target specific variations in the switching properties. However, it is important to note that altering one property typically impacts other characteristics as well [21–22].

In this regard, both the substitution of the NBD periphery to produce push–pull derivatives and the variation of the central scaffold itself have been explored. For instance, incorporation of a C_2_H_4_ moiety into the bridge led to the formation of [2.2.2]-bicyclooctadiene derivatives with increased storage capacities but significantly reduced half-lifes compared to analogues NBD derivatives [23–24]. Additionally, introduction of one or more heteroatoms at various positions of the NBD scaffold were envisioned, although such modifications have primarily been investigated theoretically thus far [25–28]. Recently, high-throughput calculations were conducted to screen a wide array of hetero-substituted NBD species. These studies predominantly considering the carbon-based scaffold as superior regarding their properties, while also highlighting some unique properties exhibited by heterocyclic derivatives [21].

C_7_-substituted NBD species have been scarcely investigated in terms of their switching behavior but have been employed as synthetic intermediates in total [29–30] and bio-synthesis protocols [31], e.g., in inositol chemistry [32] or towards aminocyclitols [33]. The potential of these compounds as functional photoswitches was primarily assessed in the 1960s and 1970s by Prinzbach and co-workers, who identified a variety of rearrangement products alongside the formation of the desired QCs [32,34–38]. Despite this, the focus has largely been on symmetrical 2,3-substituted derivatives, with reports primarily on the reversible isomerization of oxa-NBD to oxa-QC derivatives [39].

Asymmetrical push–pull derivatives have received little attention in the literature, with investigations largely limited to forward conversions [35]. Here, we present 2,3-heteroatom-incorporated NBD derivatives and compare their properties with those of previously published isocyclic derivatives. By utilizing push–pull functionalization and incorporating larger heteroatoms in the bridge moiety, we anticipate a bathochromic shift that would facilitate absorption match with the solar emission spectrum.

## Results and Discussion

### Synthesis

**Scheme 1:**
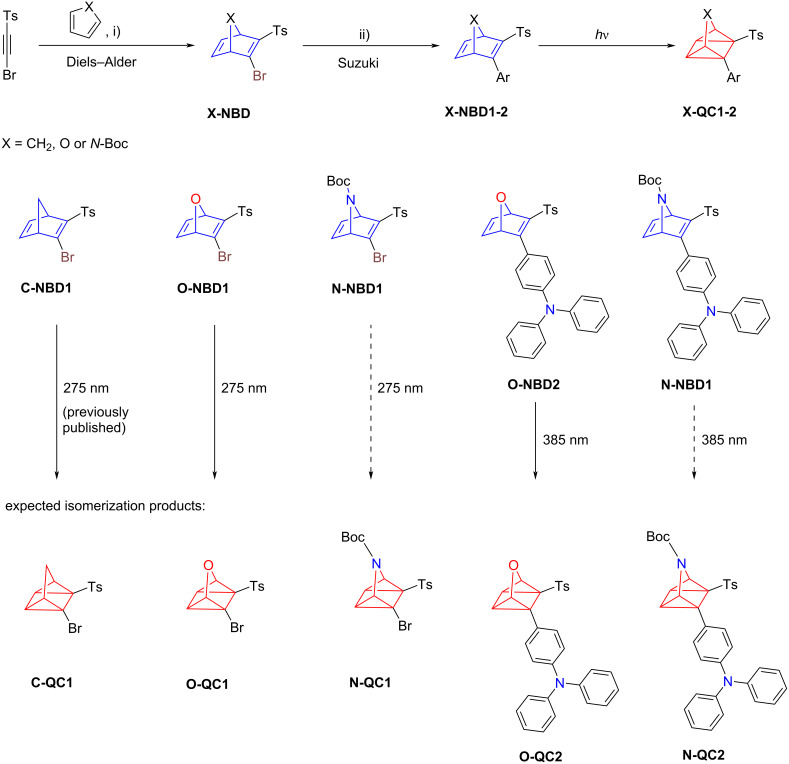
Synthetic procedure towards new **X-NBD** derivatives **C-NBD1, O-NBD1** and **N-NBD1**. 1-((Bromoethynyl)sulfonyl)-4-methylbenzene was prepared as published before [41]. i) Cyclopentadiene, toluene, rt, 24 h, 92%; furan, 45 °C, 24 h, 84%; *N*-Boc-pyrrol, 90 °C, 68 h, 53%; ii) (4-(diphenylamino)phenyl)boronic acid (1.2 equiv), K_2_CO_3_, Pd(OAc)_2_, RuPhos, 4:1 toluene/H_2_O, 80 °C , 18 h. Overview of the new NBD derivatives and the isomerization products (QCs) expected upon isomerization. Formation of **N-QC1** and **N-QC2** could not be proven.

The library of new heterocyclic NBD molecules synthesized and investigated in this study is presented in Scheme 1*.* Starting with 1-((bromoethynyl)sulfonyl)-4-methylbenzene, which was previously prepared and characterized [40–41], Diels–Alder reaction with either cyclopentadiene, furan or Boc-protected pyrrol, resulted in the NBD precursors **C-NBD1**, **O-NBD1** and **N-NBD1**, respectively [31,42]. With these precursors in hand a subsequent Suzuki cross-coupling reaction with (4-(diphenylamino)phenyl)boronic acid was performed. The reaction conditions were adapted from prior experiments with **C-NBD1** [40] and further refined for the heterocyclic analogues. Optimal results were achieved using K_2_CO_3_, Pd(OAc)_2_ and RuPhos with the corresponding boronic acid in a degassed toluene/H_2_O mixture (4:1, v/v) which was heated to 80 °C for 18 h (for detailed information see Supporting Information File 1). Using the described procedure, the oxygen containing derivatives **O-NBD2** and nitrogen substituted **N-NBD2** were successfully synthesized (Scheme 1, bottom). In contrast to the previously synthesized large library carbon-based NBDs [40], only a few heterocyclic derivatives were successfully isolated. During the monitoring of additional reactions attempting to reproduce the literature scope, in most cases traces of new NBD species were observed. However, purification was not possible. Furthermore, an alternative commonly used synthetic strategy involving the Diels–Alder reaction of a pre-functionalized push–pull acetylene with furan or pyrrol derivatives proved unsuccessful [4,43]. All synthesized NBD derivatives were characterized using UV–vis and NMR spectroscopy, as well as high-resolution mass spectrometry. An overview of the synthetic and spectroscopic data for all derivatives is provided in Table 1, while the specific spectroscopic properties of the individual QC analogues can be found in Supporting Information File 1.

**Table 1:** Overview of spectroscopic data of the newly synthesized compounds. The data given for the carbon analogues **C-NBD1** (2-bromo-3-tosylbicyclo[2.2.1]hepta-2,5-diene) and **C-NBD2** (*N*,*N*-diphenyl-4-(3-tosylbicyclo[2.2.1]hepta-2,5-dien-2-yl)aniline) were previously published [40].

Compound	Yield [%]	λ_max_ (ε)^a^ [nm]	λ_π–π*_ (ε)^a^ [nm]	λ_onset_^b^ [nm]

**C-NBD1** ^c^	92	269 (6500)	243 (12500)	317
**O-NBD1**	84	278 (4500)	239 (11200)	343
**N-NBD1**	53	289 (3800)	240 (11800)	341
**C-NBD2** ^c^	62	370 (19200)	294 (14000)	452
**O-NBD2**	29	392 (15600)	295 (14600)	498
**N-NBD2**	26	393 (17300)	294 (14200)	479

^a^Extinction coefficients are given in M^−1^·cm^−1^; ^b^onset values are defined as log (ε) = 2; ^c^data are provided for easier comparability based on literature [40].

### Photoswitching

**Figure 2:**
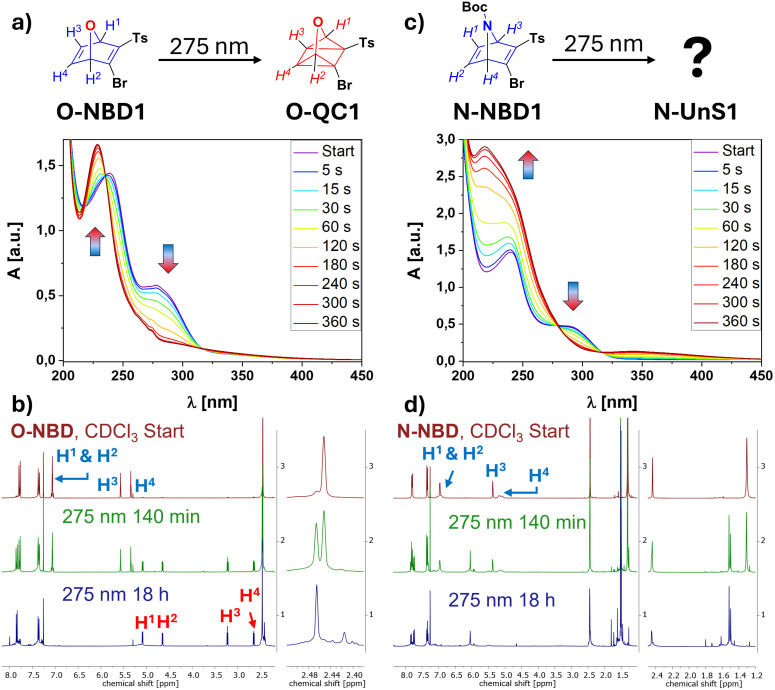
Conversion of **O-NBD1** to **O-QC1** using a 275 nm LED. The UV–vis spectrum was recorded in MeCN; b) the respective NMR spectrum was recorded in CDCl_3_; c) similar UV–vis experiment for **N-NBD1** measured in MeCN; d) conversion of **N-NBD** to an unidentified photoisomer **N-UnS1** monitored via NMR spectroscopy in CDCl_3_.

The photoswitching of the NBDs to corresponding QCs was monitored by UV–vis and ^1^H NMR spectroscopy. With increasing electronegativity of the bridge-atom, a slight bathochromic shift in the absorption spectra of the parent NBD precursors **O-NBD1** and **N-NBD1** was found (Figure 2a and 2c). For the comparison with the data obtained for **C-NBD1** we refer to previous literature studies [40]. As with **C-NBD1**, clean conversion of **O-NBD1** into the corresponding **O-QC1** was observed upon irradiation at 275 nm. The absorption maximum at λ_max_ = 278 nm decreased, correlating with a simultaneous increase in the absorbance of the QC band at λ_max_ = 229 nm. The isomerization was completed after 360 s without any indication of photodecomposition. The appearance of isosbestic points demonstrate clean conversion. To further validate these findings, similar irradiation experiments were conducted and monitored by NMR spectroscopy. Upon irradiation at 275 nm, complete formation of **O-QC1** was confirmed after 18 h. A decrease over time of the **O-NBD1** mutliplet centered signals at 7.06 ppm, 5.57 ppm and 5.35 ppm assignable to the NBD scaffold can be observed. New multiplet signals at 5.08 ppm 4.65 ppm, 3.22 ppm and 2.64 ppm belonging to the scaffold of **O-QC1** arise. Further, the singlet signal corresponding to the CH_3_ group of the tosyl moiety shifts from 2.45 ppm to 2.47 ppm. A spectrum containing approximately a 45:55 ratio of **O-NBD1**/**O-QC1** can be found after 140 minutes of irradiation (Figure 2b, green).

A different behavior was observed for **N-NBD1** (Figure 2c). In the UV–vis experiments, a decrease in the absorption maximum at λ_max_ = 289 nm was noted, accompanied by a significant emergence of a new band at λ_max_ = 218 nm. Additionally, either an increase in the baseline at wavelengths longer than 315 nm or the generation of a new absorption band with a maximum around λ_max_ = 346 nm was observed. However, the absence of isosbestic points, along with the atypical line shape of the final species after 360 seconds of irradiation, precluded the conclusion of a clean isomerization to **N-QC1**.

The corresponding ^1^H NMR experiments reveal two significant signal changes (Figure 2d). First, the CH_3_ singlet signal belonging to the tosyl group shifted from 2.44 ppm to 2.45 ppm and began to split into a pseudo-doublet. Second, the singlet signal corresponding to the nine protons of the Boc group at 1.30 ppm vanishes while two new singlet signals at 1.52 ppm and 1.50 ppm are formed, which combined integrate to nine. Due to the inversion of the nitrogen located in the bridge, not only the splitting of the signals, but also the line broadening of the signals corresponding to the NBD scaffold can be explained. Specifically, the multiplet signals at 7.06–6.88 ppm (H^1^ and H^2^) and 5.22–5.04 ppm as well as the singlet at 5.36 ppm (H^3^ and H^4^) correspond to the carbon framework of **N-NBD1**. During the experiment, these signals decreased in intensity, and a set of new signals appeared in the aromatic region, alongside a new multiplet signal in the range of 6.15–5.90 ppm. However, due to the lack of new formed aliphatic signals, and in comparison to literature [35], formation of **N-QC1** is very unlikely. Furthermore, additional signals in the region below 2.0 ppm indicate the formation of decomposition products. Studies conducted by Bansal and co-workers [44] revealed the potential formation of aza-QC derivatives at low temperatures. However, isolation of these derivatives proved difficult, as rapid rearrangement to azepine analogues occurred at temperatures exceeding 0 °C. On the basis of the quadricyclane or azepine structure, various light-, heat-, and acid-induced rearrangements have been reported [35], complicating the distinct structural determination of these compounds. In comparison to published NMR and UV–vis data, formation of neither known species can be assumed with certainty.

Last, through addition of mono-carboxylic acid-Co^II^-porphyrin (**POR**, see Supporting Information File 1), which was already available within our group [4], regeneration of the parent **N-NBD1** was unsuccessful (Supporting Information File 1). Consequently, the presence of **N-QC1** can be excluded, and the spectroscopic data likely corresponds to an unknown photoisomer (**N-UnS1**). Structural investigations using exhaustive 2D-correlation NMR spectroscopy were also inconclusive.

**Figure 3:**
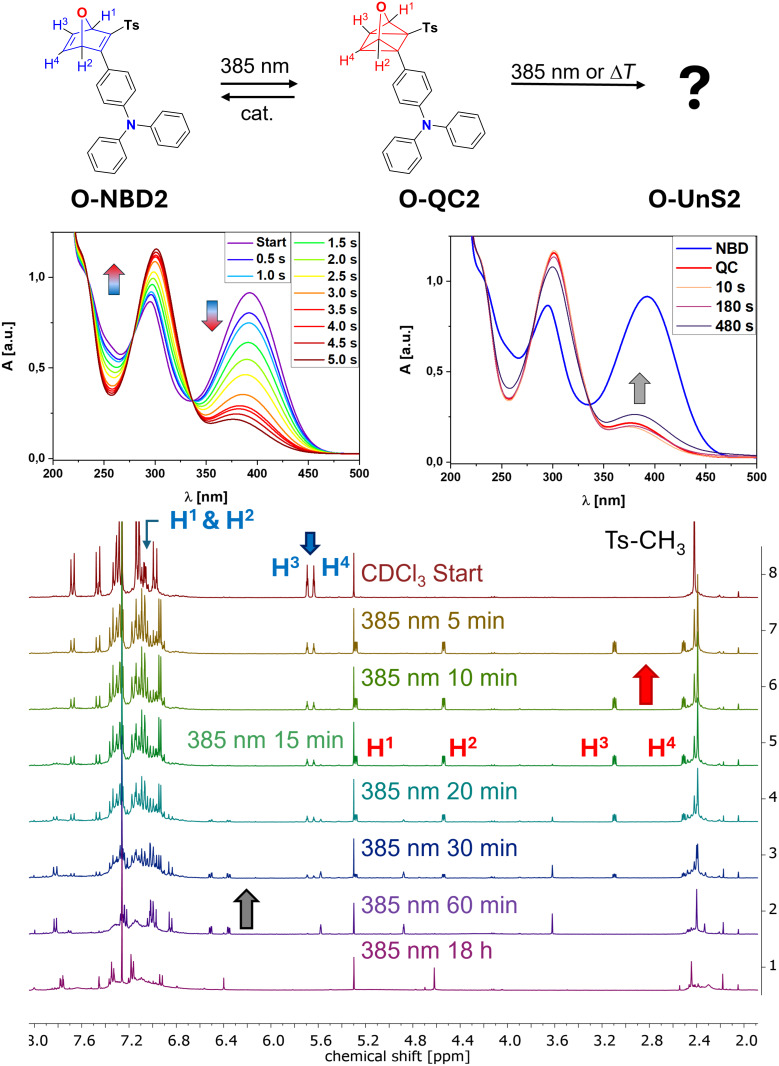
Rearrangement of **O-NBD2** to **O-QC2** using a 385 nm LED. The UV–vis measurement in the middle were conducted in MeCN, NMR spectra at the bottom with 6.9 mg substance in 650 μL CDCl_3_. Prolonged irradiation resulted first in the formation of an unidentified species **O-UnS2** (after 60 min) and further unknown rearrangement combined with photodecomposition after 18 h.

Next, the push–pull functionalized derivatives **O-NBD2** and **N-NBD2** were investigated. Notably, **N-NBD2** exhibited the most pronounced red shift (Table 1) and was the only nitrogen-containing push–pull derivative that could be synthesized in rather high quantity and purity. Monitoring first the switching process from **O-NBD2** to **O-QC2** by UV–vis spectroscopy showed complete formation of **O-QC2** was achieved after 5 seconds, indicated by the major decrease of the NBD signal at λ_max_ = 392 nm and the simultaneous increase of the QC´s band at λ_max_ = 301 nm accompanied by the presence of isosbestic points (Figure 3). Extended irradiation times resulted in a decrease in the QC signal, accompanied by a slight blue shift and the emergence of a new absorption band at λ_max_ = 382 nm, suggesting potential further rearrangements or photodecomposition.

To gain a better understanding of the ongoing conversion processes, we conducted NMR spectroscopy. The assignment of individual proton signals was supported by 2D correlation NMR spectroscopy (Supporting Information File 1). First, **O-NBD2** is characterized by the mutliplet signal around 7.07 ppm for the olefinic (H^1^ and H^2^) and the two pseudo-triplet signals at 5.69 ppm (H^3^) and 5.64 ppm (H^4^) corresponding to the bridgehead protons. In addition, a sharp singlet signal assigned to the CH_3_ protons of the tosyl group is found at 2.42 ppm like for the precursor **O-NBD1**. Upon irradiation at 385 nm, after 15 minutes, approximately 70% of **O-NBD2** are converted to **O-QC2**, indicated by the emerge of new signals at 5.28 ppm, 4.54 ppm, 3.09 ppm and 2.51 ppm, corresponding to H^1^, H^2^, H^3^ and H^4^ respectively. Furthermore, the CH_3_ singlet is shifted to 2.39 ppm. However, after longer irradiation times (60 minutes), the remaining NBD signals, along with the newly formed QC signals, completely vanish, and a new set of signals corresponding to an unidentified species (**O-UnS2**) appears, as already noted during the UV–vis analysis.

Prinzbach, Stusche and Vogel have described a variety of potential rearrangements for oxa-NBD and oxa-QC derivatives [32,34,36–37]. Therefore, in-depth 2D NMR analysis combined with mass spectrometry was attempted to elucidate the structure of **O-UnS2**. Notably, an additional singlet proton signal (integrated as 1 proton) corresponding to the unidentified species was observed, which cannot be present in the structure of **O-QC2**. Further irradiation over 18 hours resulted in additional rearrangements or photodecomposition, which were not analyzed further.

At slightly lower concentrations (5.0 mg substance in 650 μL CDCl_3_) quantitative formation of **O-QC2** was achieved after 15 minutes (Supporting Information File 1). Next, thermally induced back-conversion to **Q-NBD2** was attempted by heating the sample to 50 °C overnight. This process resulted in the initial formation of **O-UnS2**, followed by further rearrangement, consistent with the observations made during prolonged irradiation experiments. Due to the thermal instability of **O-QC2** noted during the investigation, kinetic experiments for determining the half-life time *t*_1/2_ were not conducted. However, the addition of Co^II^-porphyrin **POR** allowed for the successful regeneration of the parent **O-NBD2** (Supporting Information File 1), demonstrating the intermediate availability of **O-QC2**.

**Figure 4:**
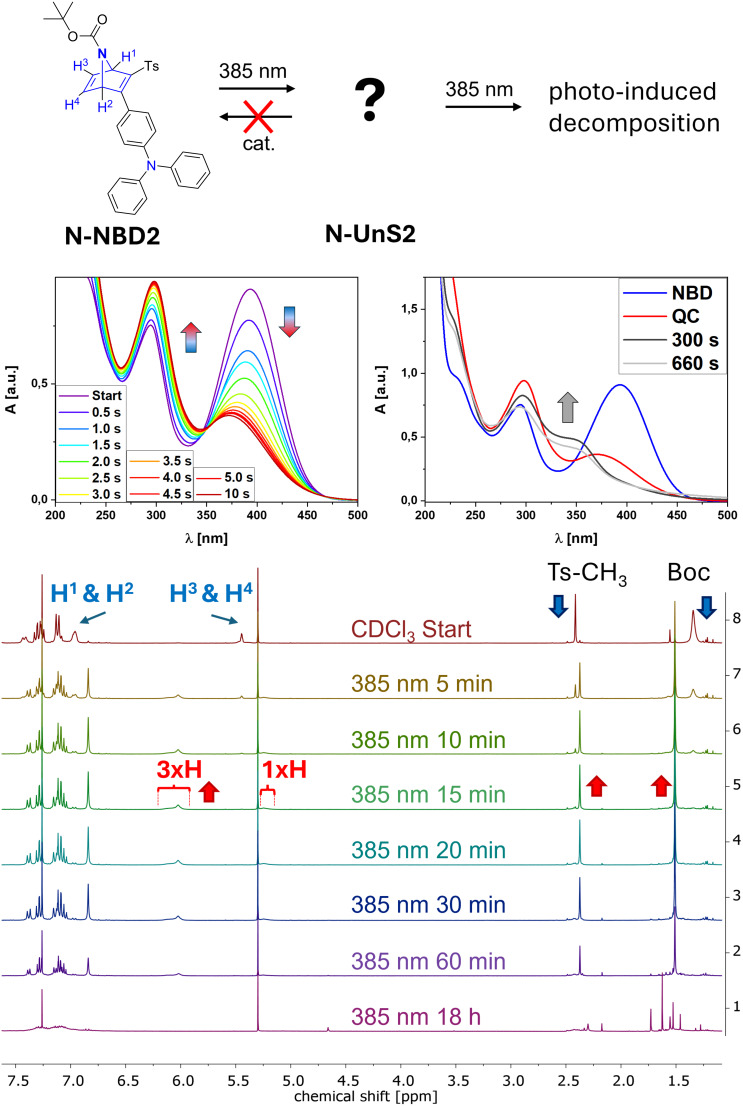
Rearrangement of **N-NBD2** using a 385 nm LED. The UV–vis measurement in the middle were conducted in MeCN, NMR spectra at the bottom with 6.9 mg substance in 650 μL CDCl_3_. Generation of the new photoisomer **N-UnS2** is completed after 20 minutes, while further irradiation induces photodecomposition.

In a similar manner, the aza analogue **N-NBD2** was investigated. The UV–vis experiments (Figure 4) yielded results comparable to those obtained for **N-NBD1**. Initially, the conversion to **N-QC2** is anticipated at short time scales with irradiation at 385 nm, as indicated by a decrease in the NBD signal at λ_max_ = 393 nm. However, during this initial time segment, no isosbestic points developed, and no distinct new QC signal was detected. Instead, there was a slight redshift and an increase in the previously observed π–π* absorption from λ_π–π*_ = 294 nm to 298 nm was found. Prolonged irradiation up to 660 s led to a complete change in the overall absorption properties with maxima at λ = 348 nm and 297 nm and an absorption shoulder at λ = 228 nm suggesting photodecomposition.

Next, NMR monitoring of the irradiation experiment was conducted. The signal sets for **N-NBD2** were generally similar to those of **O-NBD2**. As previously described, the possibility of inversion of the nitrogen's lone pair led to significant line broadening, particularly evident in the proton signals of the carbon scaffold near the heteroatom. As an additional significant signal, the singlet at 1.34 ppm corresponding to the nine protons of the Boc group can be found. After 5 minutes of irradiation, the majority of **N-NBD2** is consumed, finished after 15 minutes with the complete generation of a newly formed species. The spectral features were largely consistent with those described for **N-NBD1**, suggesting the formation of a similar unidentified species, designated as **N-UnS2**. Again, comprehensive 2D NMR spectroscopic investigations did not deliver satisfactory results unravelling the structure. Further irradiation first seems to induce no additional spectroscopic changes, while the initially yellow solution turned deep black in the time frame between 15 and 60 minutes (Supporting Information File 1). Prolonged irradiation overnight ultimately resulted in complete photodecomposition, indicated by the absence of significant signals corresponding to either **N-NBD2** and **N-UnS2**. Addition of the previously used Co^II^-catalyst **POR** did not result in regeneration of the parent **N-NBD2** species strongly excluding the presence of **N-QC2**.

Given the challenges in generating heterocyclic QC derivatives – successfully achieved only for oxa derivatives – the investigation of photophysical key properties for molecular solar thermal (MOST) systems was not pursued at this stage. The limited availability of material, combined with the thermal instability of the formed QC species, precluded the determination of half-lifes and storage densities. Additionally, as all NBD to QC conversions were either incomplete due to photodecomposition or led to the formation of additional species, it was not feasible to ascertain isomerization quantum yields.

## Conclusion

In summary, we have presented the synthesis and photophysical properties of a library of heterocyclic push–pull NBD derivatives. All interconversions were studied by UV–vis spectroscopy and confirmed with ^1^H NMR measurements. Both oxa-NBD derivatives could be converted to the QC counterparts upon irradiation. A detailed description of the conversion of **O-NBD2** to **O-QC2** is provided, noting that **O-QC2** further isomerizes into another species with prolonged irradiation. The formation of the same species was also observed while attempting the back-isomerization of **O-QC2** to **O-NBD2** at elevated temperatures. However, prolonged heating resulted in rearrangement to a 4^th^ unidentified species. Clean recovery of **O-NBD2** from the respective QC derivative was accomplished by addition of a Co^II^-porphyrin catalyst. For the nitrogen containing NBDs **N-NBD1** and **N-NBD2** photoinduced generation of an unidentified species was observed, followed by complete photodecomposition upon extended irradiation. The formation of **N-QC2** was excluded, as evidenced by the unsuccessful attempts to catalytically back-convert it. The distinct determination of the unknown structures was attempted through exhaustive NMR spectroscopy, however, these efforts were ultimately unsuccessful. When compared to literature values, a notable redshift in the absorption maximum and onset was observed for the heterocyclic derivatives in relation to their isocyclic analogues.

## Supporting Information

File 1 Experimental, characterization data, copies of NMR spectra and switching experiments.

## Data Availability

Data generated and analyzed during this study is available from the corresponding author upon reasonable request.
