# Supplementary material for: Synthesis and photoinduced switching properties of C7-heteroatom containing push–pull norbornadiene derivatives
Source: Beilstein J Org Chem. 2025 Apr 22;21:807–16. doi: 10.3762/bjoc.21.64 (PMC12035880; doi:10.3762/bjoc.21.64)
Supplement: File 1 — Experimental, characterization data, copies of NMR spectra and switching experiments. [file Beilstein_J_Org_Chem-21-807-s001.pdf]

BEILSTEIN JOURNAL OF ORGANIC CHEMISTRY

Supporting Information

for

Synthesis and photoinduced switching properties of C_7-heteroatom containing push–pull norbornadiene derivatives

Daniel Krappmann and Andreas Hirsch

Beilstein J. Org. Chem. **2025,** *21,* 807–816. doi:10.3762/bjoc.21.64

Experimental, characterization data, copies of NMR spectra and switching experiments

License and Terms: This is a supporting information file under the terms of the Creative Commons Attribution License (https://creativecommons.org/licenses/by/4.0). Please note that the reuse, redistribution and reproduction in particular requires that the author(s) and source are credited and that individual graphics may be subject to special legal provisions.
The license is subject to the *Beilstein Journal of Organic Chemistry* terms and conditions: (https://www.beilstein-journals.org/bjoc/terms)

Table of Contents

1.1 Material and Methods S2

1.2 Syntheses S4

1.2.1 C-NBD1 and acetylene precursors....S5

1.2.2 POR [5-(p-Carboxyphenyl)-10,15,20-(p-tert-butyltriphenylphenyl)porphyrinato] cobalt (II) (carboxylic acid A_3B Cobalt (II) -Porphyrin) S5

1.2.3 O-NBD1 (2-bromo-3-tosyl-7-oxabicyclo[2.2.1]hepta-2,5-diene) S5

1.2.4 N-NBD1 (*tert*-butyl-2-bromo-3-tosyl-7-azabicyclo[2.2.1]hepta-2,5-diene-7-carboxylate) S8

1.2.5 O-NBD2 (*N,N*-diphenyl-4-((1S,4R)-3-tosyl-7-oxabicyclo[2.2.1]hepta-2,5-dien-2-yl)aniline) S16

1.2.6 N-NBD2 (tert-butyl (1S,4R)-2-(4-(diphenylamino)phenyl)-3-tosyl-7-azabicyclo[2.2.1]hepta-2,5-diene-7-carboxylate) S21

1.2.7 C-QC1 (1-bromo-5-tosyltetracyclo[3.2.0.0^{2,7}.0^{4,6}]heptane) S27

1.2.8 O-QC1 (1-bromo-5-tosyl-3-oxatetracyclo[3.2.0.0^{2,7}.0^{4,6}]heptane)....S27

1.2.9 N-QC1 (*tert*-butyl 1-bromo-5-tosyl-3-azatetracyclo[3.2.0.0^{2,7}.0^{4,6}]heptane-3-carboxylate)....S29

1.2.10 O-QC2 (*N,N*-diphenyl-4-(5-tosyl-3-oxatetracyclo[3.2.0.0^{2,7}.0^{4,6}]heptan-1-yl)aniline) S29

1.2.11 N-QC2 (*tert*-butyl 1-(4-(diphenylamino)phenyl)-5-tosyl-3-azatetracyclo[3.2.0.0^{2,7}.0^{4,6}]heptane-3-carboxylate S34

1.3 Switching Experiments....S34

1.3.1 C-NBD1 to C-QC1....S34

1.3.2 O-NBD1 to O-QC1....S35

1.3.3 N-NBD1 to N-QC1/N-UnS1 ....S37

1.3.4 O-NBD2 to O-QC2 and further rearrangement to O-UnS2....S40

1.3.5 N-NBD2 to N-QC2/N-UnS2 ....S44

1.4 References S47

1.1 Material and Methods

All reactions involving moisture or oxygen sensitive compounds were carried out under an inert gas atmosphere of nitrogen or argon while using anhydrous solvents and standard Schlenk techniques. All used solvents were distilled on a rotary evaporator before usage. All required chemicals were purchased from commercial suppliers Sigma-Aldrich (St. Louis, Mo, USA), ABCR (Karlsruhe, Germany), VWR (Darmstadt, Germany), Acros Organics (Geel, Belgium) or Roth (Karlsruhe, Germany) and used directly without further purification. TLC analysis was performed on aluminum plates coated with 0.20 mm Merck® silica gel 60 containing a fluorescent indicator (Machery-Nagel, ALUGRAM®, SILG/UV_{254}). Spots on TLC plates were visualized by exposure to ultraviolet light (λ = 254 nm and 366 nm). Purification via column chromatography was performed on silica gel 60M deactivated (0.04–0.063 mm / 230–400 mesh ASTM) from Macherey-Nagel®, Düren, Germany.

Flash column chromatography: Flash column chromatography was performed with a Biotage® Selekt, with the software SELEKT 1.1.1.-13044 using pre-packed columns purchase from Büchi (St. Gallen, Switzerland).

NMR: All NMR spectra were recorded on a BRUKER Avance gX (^{1}H NMR: 300 MHz, 400 MHz, 500 MHz or 600 MHz; ^{13}C NMR: 75 MHz, 100 MHz, 125 MHz or 151 MHz). All measurements were performed at room temperature if not stated otherwise. Chemical shifts δ are reported in ppm and were referenced to the residual solvent signal as an internal reference (*Table S1*). Solvent impurities were determined according to the work of *Fulmer et. al*.

Table S1 List of deuterated solvents used for ^{1}H- and ^{13}C-NMR spectroscopy. The respective residual solvent signal was calibrated on the following values [1].

**Solvent**	δ_H **[ppm]**	δ_C **[ppm]**
$CDCl_3$	7.26	77.16
Toluene-d_8	2.08	20.43
Acetonitrile-d_3	1.94	118.26

Progressing of the raw data was done with MestReNova [2]. During the characterization of the signal multiplicities the following abbreviations were used: s = singlet, d = doublet, dd = doublet of doublet, ddd = doublet of doublet of doublet, t = triplet, q = quartet, m = multiplet. ^{13}C-NMR spectra were recorded by broadband decoupling.

High-resolution mass spectrometry (HRMS): MS was performed on a BRUKER DALTONICS MaXis4g (ESI & APPI). As ionization method either ESI or atmospheric pressure photoionization (APPI) was used. For

detection of the formed ions Time of Flight (TOF) was performed. The mass to charge ratios *m/z* are given in u.

UV–vis spectroscopy: UV–vis spectroscopy was carried out on a Varian Carry 5000 UV–vis-NIR spectrometer. The baseline and zero transmittance were calibrated before measurements were taken. A 10 × 10 mm high precision cell from Hellma®Analytics, Müllheim, Germany was used. All measurements were conducted using HPLC grade MeCN if not stated otherwise. λ_{onset} values are determined as log(ε) = 2.

LED/Irradiation:

For all switching experiments, a in-house built irradiation setup was used [3]. All utilized LED were purchased from Neumüller Elektronik GmbH® or Conrad Electronics SE. The power of the used LEDs in respective to their emission wavelength are given for the maximum current in *Table S2*. The samples were irradiated directly in the NMR tube or a Quartz glass cuvette for NMR and UV–vis switching studies, respectively. The distance between sample and LED was kept at 1.5 cm. For irradiation experiments, the maximum power of every LED was tied to use (specifications are provided in *table S2*).

Table S 2: LEDs used in the home-made irradiation setup.

Wavelength [nm]	Article Number (Supplier)	Max. Power and characteristics	Measured current during the experiment
275	CUD8AF4D (Neumüller Elektronik GmbH)	60 mW at 600 mA / 6.4V	6.9-7.4 V with 0.31-0.52 A
385	CUN86A1B (Neumüller Elektronik GmbH)	1160 mW at 500 mA / 3.5V	3.2-3.3 V with 0.32-0.45 A

Onset irradiation for N-NBD1 and N-NBD2 with 310 nm and 425 nm, respectively, were performed using the Lucent360^{TM} Advanced Photoreactor by HepatoChem. Each LED block 310 nm and 425nm (approx. maximal power = 50 W) were used at 80% performance. The samples were directly irradiatied in the NMR tubes which were placed in the screening holder of the instrument while cooling the sample to 15 °C.

Melting points: melting points (mp) were rmeasured on a MPM-H3 melting point meter from "Schorpp Gerätetechnik" in visual determination mode and a heating rate of 1 °C/min.

1.2 Syntheses

All experiments under inert atmosphere were carried out using standard Schlenk technique.

General cross-coupling procedure A [4]:

The general Suzuki cross-coupling procedure was adapted from literature [4]. 2-bromo-3-tosylbicyclo[2.2.1]hepta-2,5-diene[5] (1.0 equiv), boronic acid (1.2 equiv), K_2CO_3 (4.6. equiv), RuPhos (0.1 equiv) and $Pd(OAc)_2$ (5 mol %) were submitted in a flame dried, nitrogen containing pressure tube. Toluene (10 mL) and H_2O (2.5 mL) were purged with N_2 for 30 min and added to the vial. The obtained biphasic reaction mixture was heated to 80 °C for up to 18 h or until TLC analysis showed full consumption of the staring material. Afterwards, the reaction was cooled to room temperature and the solvents removed under reduced pressure. The residue was adsorbed on SiO_2 and subjected to automatized flash column chromatography.

In a manner analogous to [3] **the general switching procedure B for NMR experiments was performed as follows**:

Photoisomerization towards QC´s was achieved using a home-made irradiation apparatus verified *via* ^{1}H NMR spectroscopy [6]. The respective NBD (5.0–10.0 mg) was dissolved in 650 μL (750 μL in case of MeCN) of the respective deuterated solvent and transferred into a NMR test tube. One initial NMR was measured of each freshly prepared sample prior to the irradiation studies. The NMR test tube was irradiated using the given LED lamp for a certain period of time. After each irradiation step, an additional ^{1}H NMR spectrum was measured to verify the conversion. This procedure was carried on until full conversion, no further conversion or photodecomposition was observed.

In a manner analogous to [3] **the general switching procedure C for UV–vis experiments was performed as follows**:

Photoisomerization towards QC´s was achieved using a home-made irradiation apparatus exclusively [6]. The respective NBD was dissolved in MeCN obtain an approximate concentration of 6.0×10^{-5} M. The prepared sample was transferred into a quartz glass cuvette and irradiated using the given LED lamp for the stated period of time. UV–vis spectra were recorded prior irradiation and after each irradiation step. This procedure was carried on until full conversion, no further conversion or photodecomposition was.

1.2.1 C-NBD1 and acetylene precursors

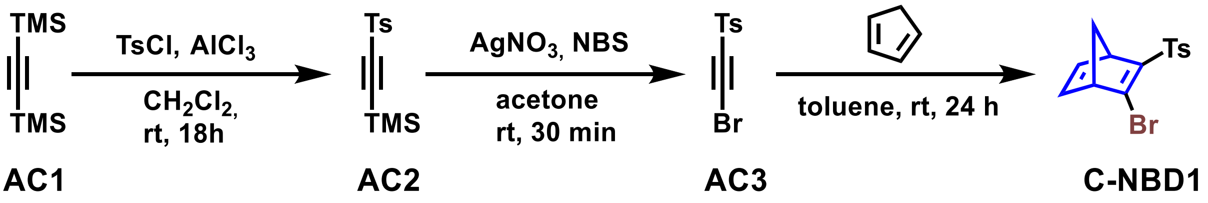

*Scheme 1: Synthesis of acetylene precursors **AC1–AC3** and **C-NBD1.***

The acetylene precursors AC1–AC3 were prepared according to literature [5]. Complete characterization or all acetylenes as well as the photophysical investigation of **C-NBD1** and **C-QC1** was published before and are in agreement with the reported analysis [3].

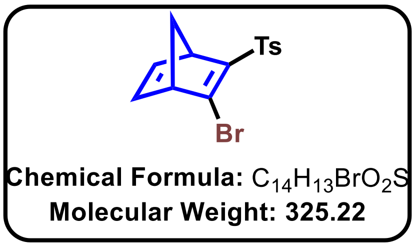

C-NBD1 was obtained as described previously [3]. The measured spectroscopic features are in accordance to literature [3].

mp: 81.5-83.1 °C.

1.2.2 POR [5-(*p*-Carboxyphenyl)-10,15,20-(*p*-*tert*-butyltriphenylphenyl)porphyrinato] cobalt (II) (carboxylic acid A_3B Cobalt (II) -Porphyrin)

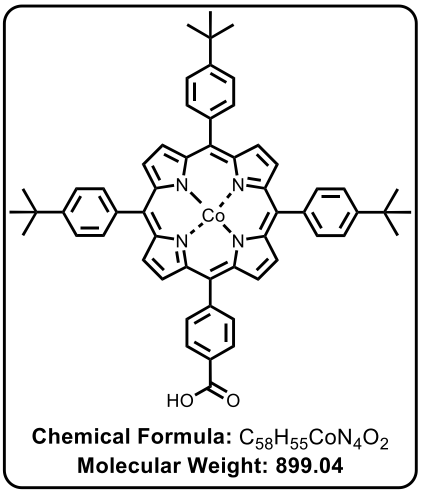

Carboxylic acid A_3B Cobalt (II) -Porphyrin (**POR**) which was used to investigate the catalytic back reaction of QC to NBD was already available in our group and prepared according to literature [7].

1.2.3 O-NBD1 (2-bromo-3-tosyl-7-oxabicyclo[2.2.1]hepta-2,5-diene)

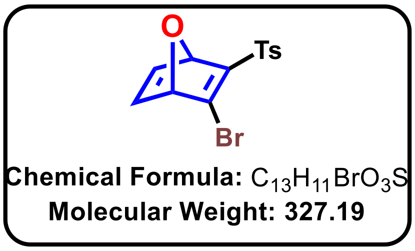

O-NBD1 was prepared according to literature [5,8,9]. In a flame dried apparatus under N_2 atmosphere, **AC3** (3.86 mmol, 1.00 g, 1.0 equiv) was combined with furan (46.3 mmol, 3.35 mL, 12 equiv) and heated to 45 °C for 24 h. Afterwards, the excessive furan was removed under reduced pressure. The crude residue was purified

via automized flash column chromatography using 20% EtOAc in hexane as eluent. The target compound was obtained as slightly beige solid in 84 % yield.

R$_f$ = 0.26 (hexanes/EtOAc 4:1)

^{1}H-NMR (400 MHz, $CDCl_3$, 25 °C) δ_H [ppm]: 7.80-7.76 (m, 2H), 7.38-7.34 (m, 2H), 7.08-7.04 (m, 2H), 5.57 (t, *J* = 1.6 Hz, 1H), 5.34 (t, *J* = 1.6 Hz, 1H), 2.45 (s, 3H).

^{13}C-NMR (100 MHz, $CDCl_3$, 25 °C): (δ) [ppm] = 150.7, 146.2, 145.5, 143.8, 140.4, 136.2, 130.2, 127.9, 90.6, 85.3, 21.9.

UV–vis: λ_{max} (ε in M^{-1} cm^{-1}) = 278 nm (4500) nm, $\lambda_{\pi-\pi^*}$ = 239 nm (11200), λ_{onset} = 343 nm.

HRMS (APPI): calc. for ($C_{13}H_{12}BrO_3S$): 326.9685; found *m/z* = 326.9685 $[M+H]^+$.

mp: 62.1-65.6 °C.

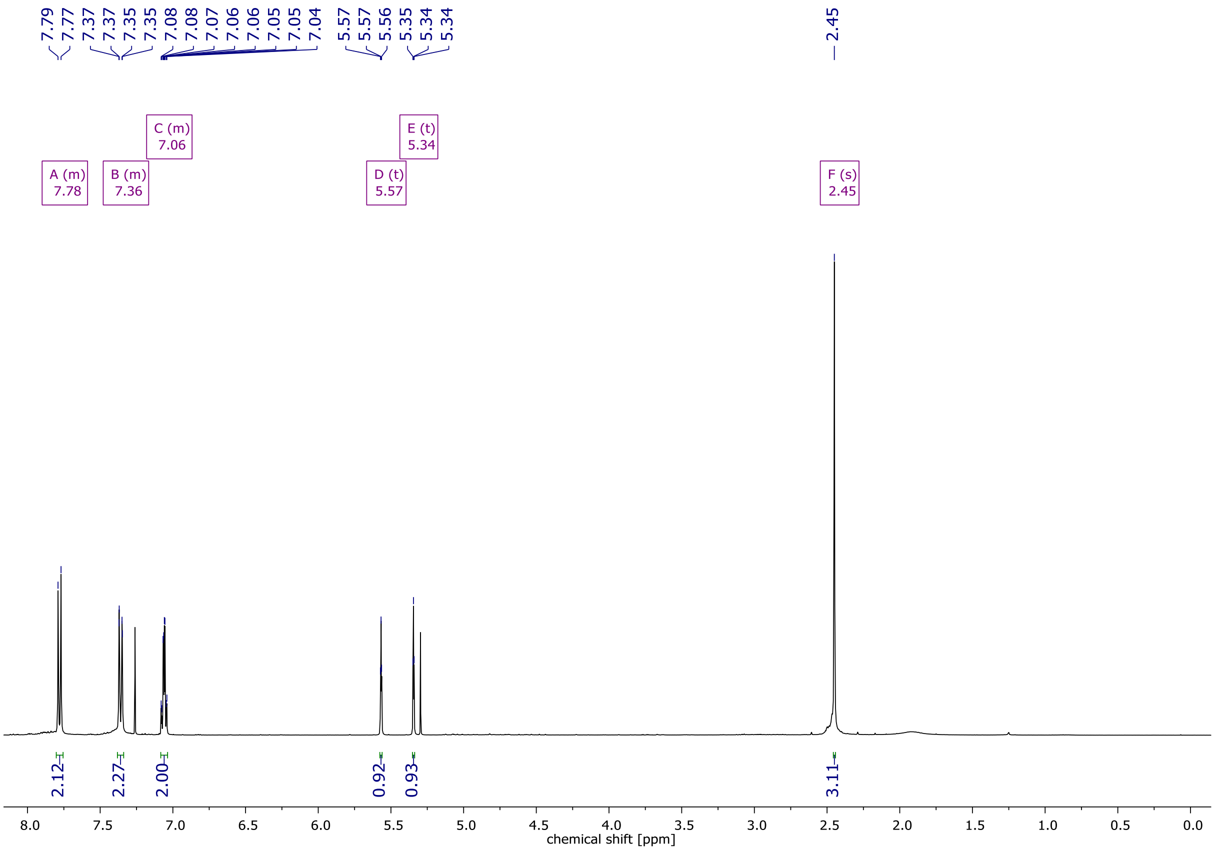

*Figure S1: ^{1}H-NMR spectrum of **O-NBD1** measured in $CDCl_3$ (400 MHz, rt).*

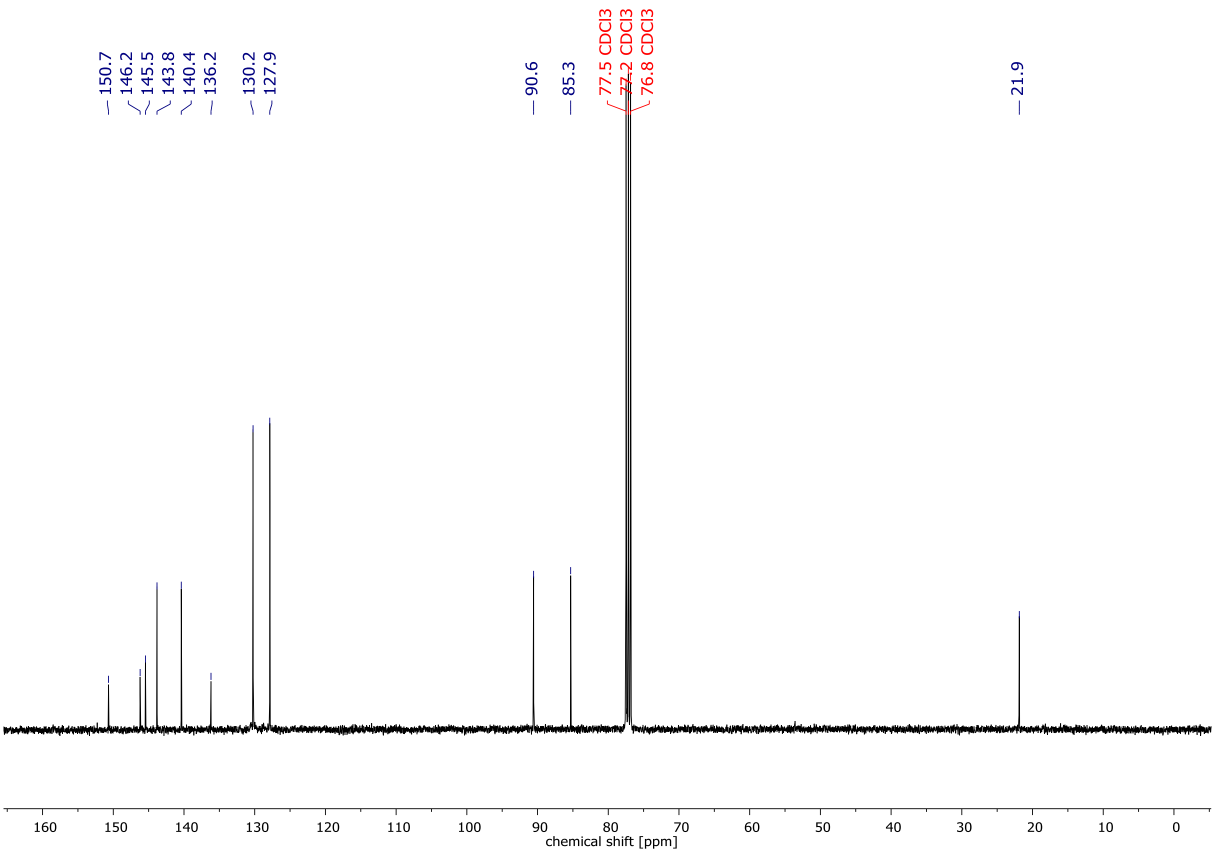

Figure S2: ^{13}C-NMR spectrum of ***O-NBD1*** *measured in in $CDCl_3$ (100 MHz, rt).*

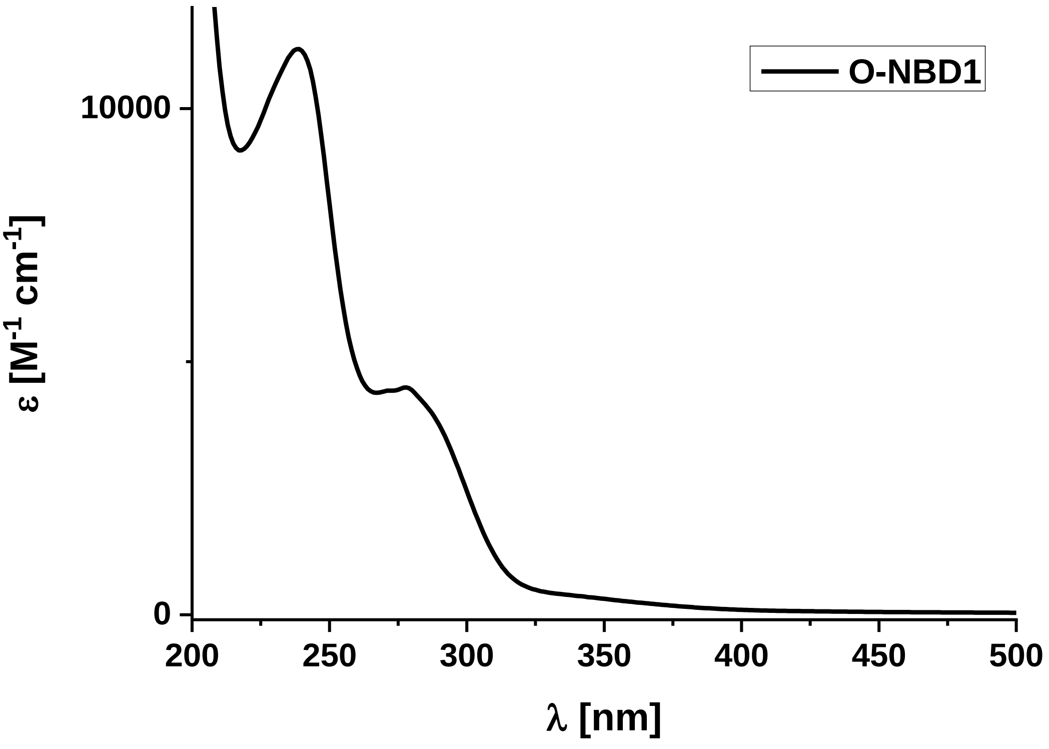

Figure S3: UV–vis spectrum of ***C-NBD1*** *measured in MeCN; ε (278 nm) = 4500 $^{l}/_{mol*cm}$;, ε (239 nm) = 11200 $^{l}/_{mol*cm}$.*

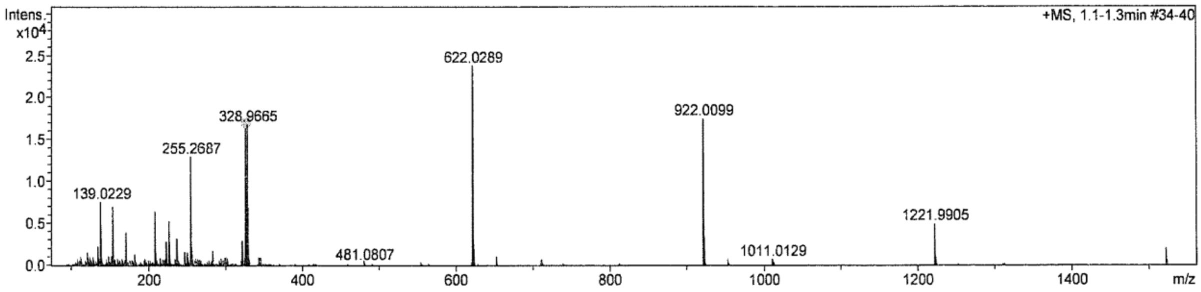

Meas. m/z	#	Ion Formula	m/z	err [ppm]	mSigma	# mSigma	Score	rdb	e⁻ Conf	N-Rule
326.9685	1	C13H12BrO3S	326.9685	0.1	7.4	1	100.00	7.5	even	ok

*Figure S4: HRMS (APPI) of **O-NBD1**.*

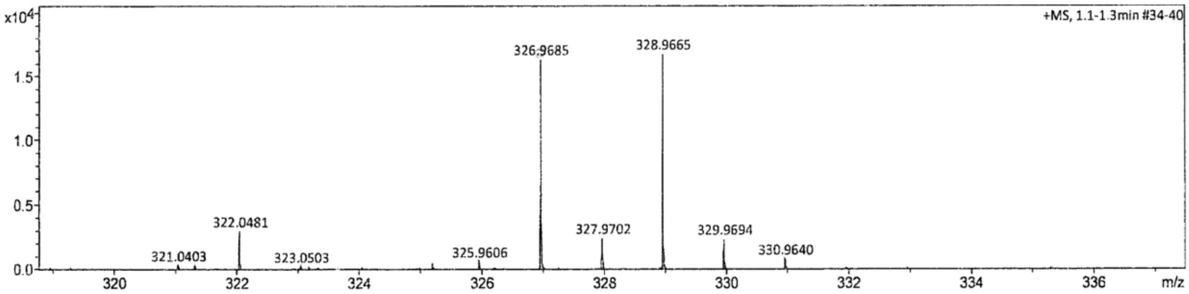

*Figure S5: Zoomed section of HRMS (APPI) of **O-NBD1**.*

1.2.4 N-NBD1 (*tert*-Butyl-2-bromo-3-tosyl-7-azabicyclo[2.2.1]hepta-2,5-diene-7-carboxylate)

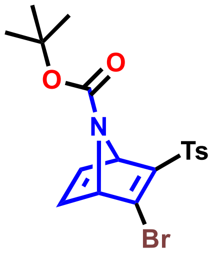

Chemical Formula: $C_{18}H_{20}BrNO_4S$
Molecular Weight: 426.33

N-NBD1 was prepared according to literature [5,8,9]. In a flame dried apparatus under N_2 atmosphere, **AC1** (8.49 mmol, 2.20 g, 1.0 equiv) was combined with *N*-Boc-pyrrol (102 mmol, 17.1 mL, 12 equiv) and heated to 90 °C for 68 h. Afterwards, the excessive *N*-Boc-pyrrol was removed under reduced pressure. The crude residue was purified via automized flash column chromatography using 20% EtOAc in hexane as eluent. The target compound was obtained as yellow liquid which solidifies in the freezer in 53 % yield.

R_f = 0.43 (hexanes/EtOAc 5:1)

^{1}H-NMR (500 MHz, $CDCl_3$, 25 °C) δ_H [ppm]: 7.83-7.78 (m, 2H), 7.37-7.33 (m, 2H), 7.08-6.88 (m, 2H), 5.37 (s, 1H), 5.24-5.04 (m, 1H), 2.44 (s, 3H), 1.31 (s, 9H).

^{13}C-NMR (125 MHz, $CDCl_3$, 25 °C): (δ) [ppm] = 153.7, 145.2, 142.7, 139.7, 136.3, 130.0, 127.9, 82.0, 75.5, 69.6, 27.8, 21.7.

UV–vis: λ_{max} (ε in M^{-1} cm^{-1}) = 289 nm (3800) nm, $\lambda_{\pi-\pi^*}$ = 240 nm (11800), λ_{onset} = 341 nm.

HRMS (ESI): calc. for $(C_{18}H_{21}BrNO_4S)^+$: 426.0369; found *m/z* = 426.0370 $[M+H]^+$. Calc. for $(C_{18}H_{20}BrNNaO_4S)$: 448.0189; found 448.0195.

HRMS (APPI): calc. for ($C_{18}H_{20}BrNNaO_4S$): 448.0189; found m/z = 448.0188 $[M+Na]^+$.calc. for ($C_{18}H_{20}BrNKO_4S$): 463.9928; found m/z = 463.9926 $[M+K]^+$.

mp: 92.4-93.7 °C.

Due to the possible inversion of the nitrogen atom, additional NMR spectroscopy was recorded at low temperatures to generate a splitting of the two signal sets.

^{1}H-NMR (main isomer, green) (600 MHz, Toluene-d_8, -10 °C) δ_H [ppm]: 7.72-7.67 (m, 2H), 6.67-6.64 (m, 2H), 6.41-6.38 (m, 1H), 6.12-6.09 (m, 1H), 5.39-5.37 (m, 1H), 5.00-4.98 (m, 1H), 1.83 (s, 3H), 1.26 (s, 9H).

^{1}H-NMR (side isomer, purple) (600 MHz, Toluene-d_8, -10 °C) δ_H [ppm]: 7.85-7.80 (m, 2H), 6.74-6.69 (m, 2H), 6.54-6.50 (m, 1H), 6.21-6.16 (m, 1H), 5.55-5.51 (m, 1H), 4.71-4.67 (m, 1H), 1.84 (s, 3H), 1.12 (s, 9H).

^{13}C-NMR* (151 MHz, Toluene-d_8, -10 °C): (δ) [ppm] = 154.6, 154.3, 153.4, 151.4, 147.2, 145.5, 144.9, 144.8, 143.6, 142.8, 139.9, 139.7, 130.4, 130.2, 81.9, 81.3, 76.7, 76.3, 70.7, 70.7, 28.1, 28.0.

**Signal set for both conformers given without separation.*

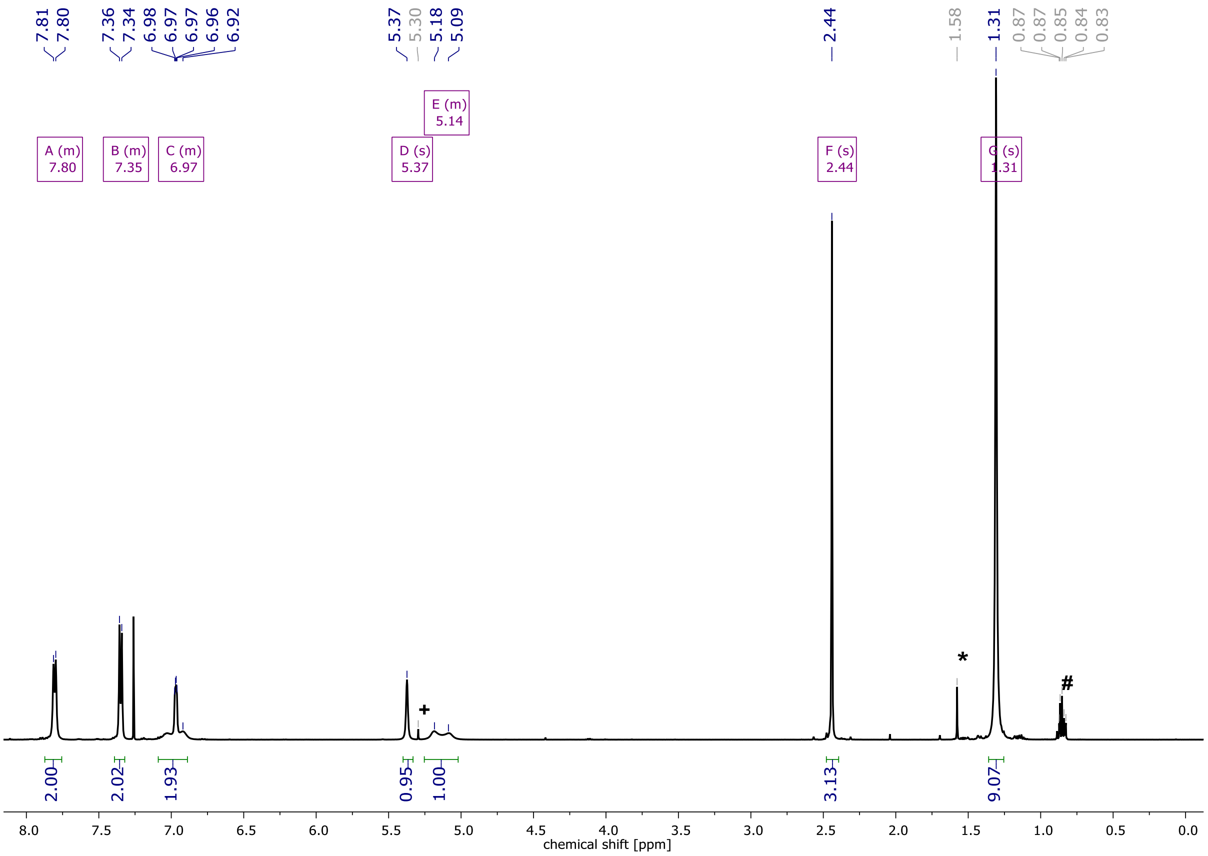

*Figure S6: ^{1}H-NMR spectrum of **N-NBD1** measured in in $CDCl_3$ (500 MHz, rt). The additional impurity signals found at 5.30 ppm (plus), 1.58 ppm (star) and 0.87-0.83 ppm (hashtag) can be assigned to methylene chloride, water and H grease, respectively.*

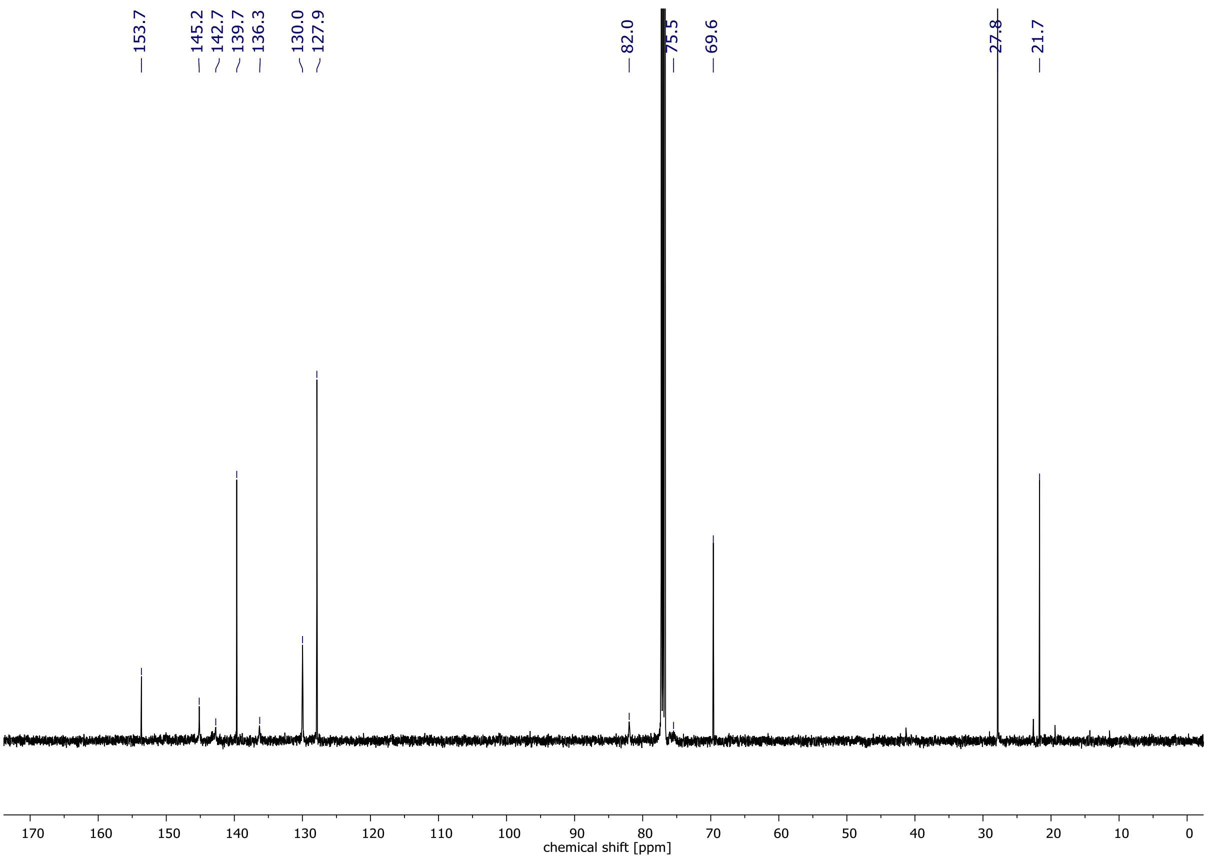

Figure S7: ^{13}C-NMR spectrum of ***N-NBD1*** *measured in in $CDCl_3$ (125 MHz, rt).*

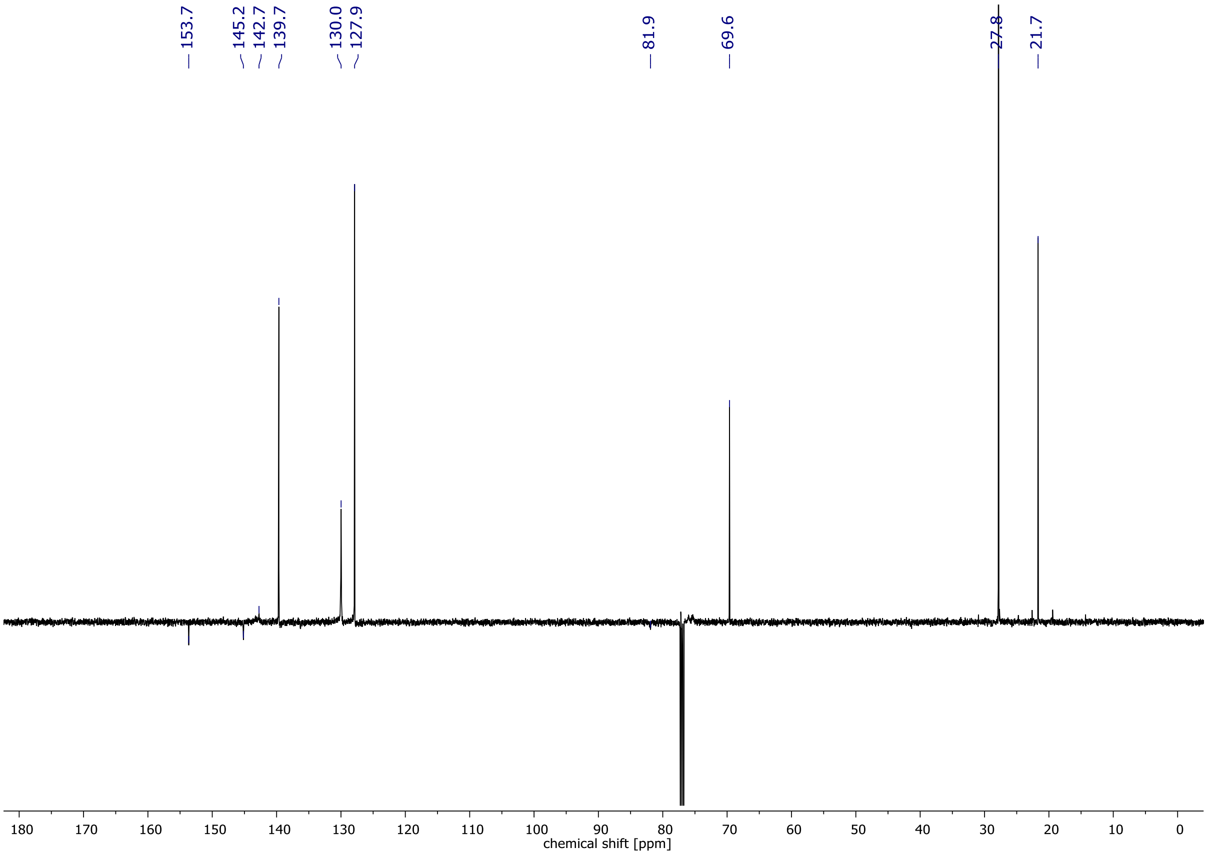

Figure S8: DEPTq spectrum of ***N-NBD1*** *measured in in $CDCl_3$ (125 MHz, rt).*

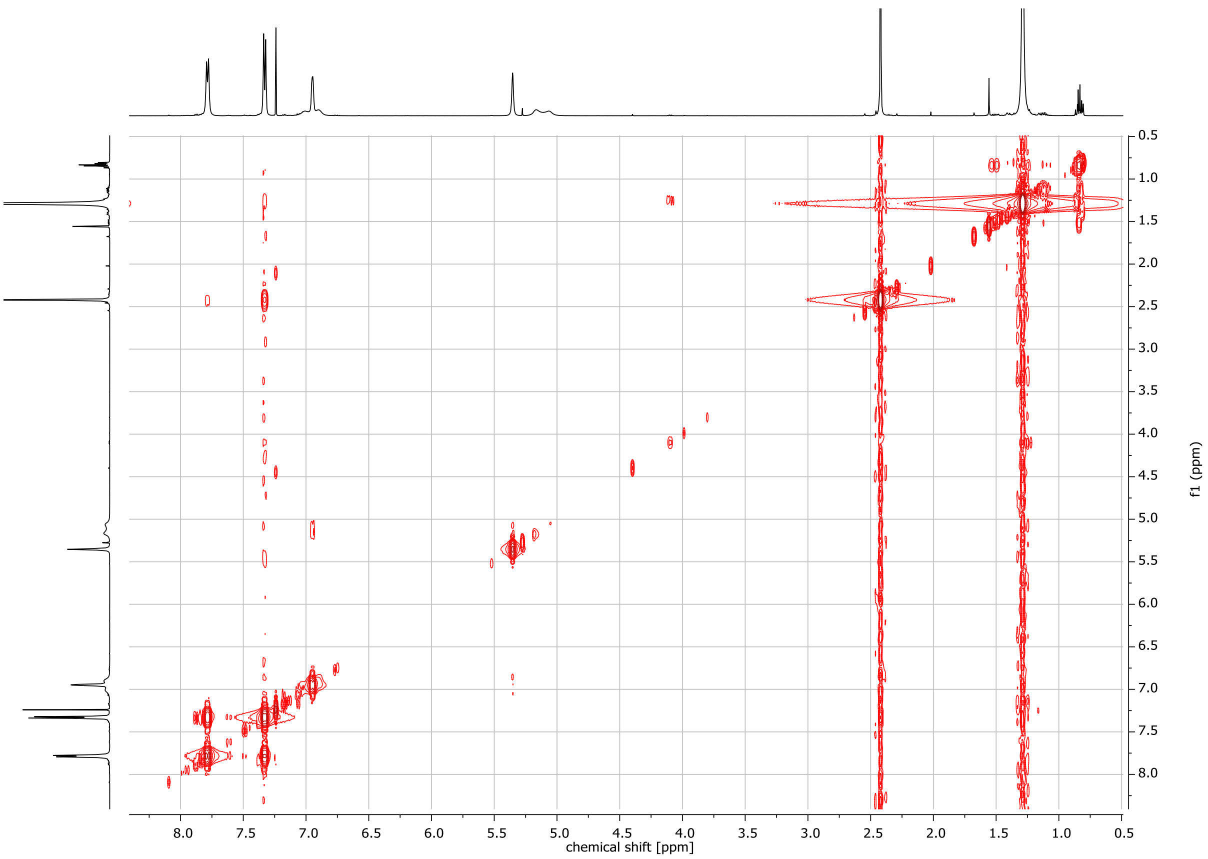

Figure S9: COSY spectrum of ***N-NBD1*** *measured in in $CDCl_3$ (500 MHz, rt).*

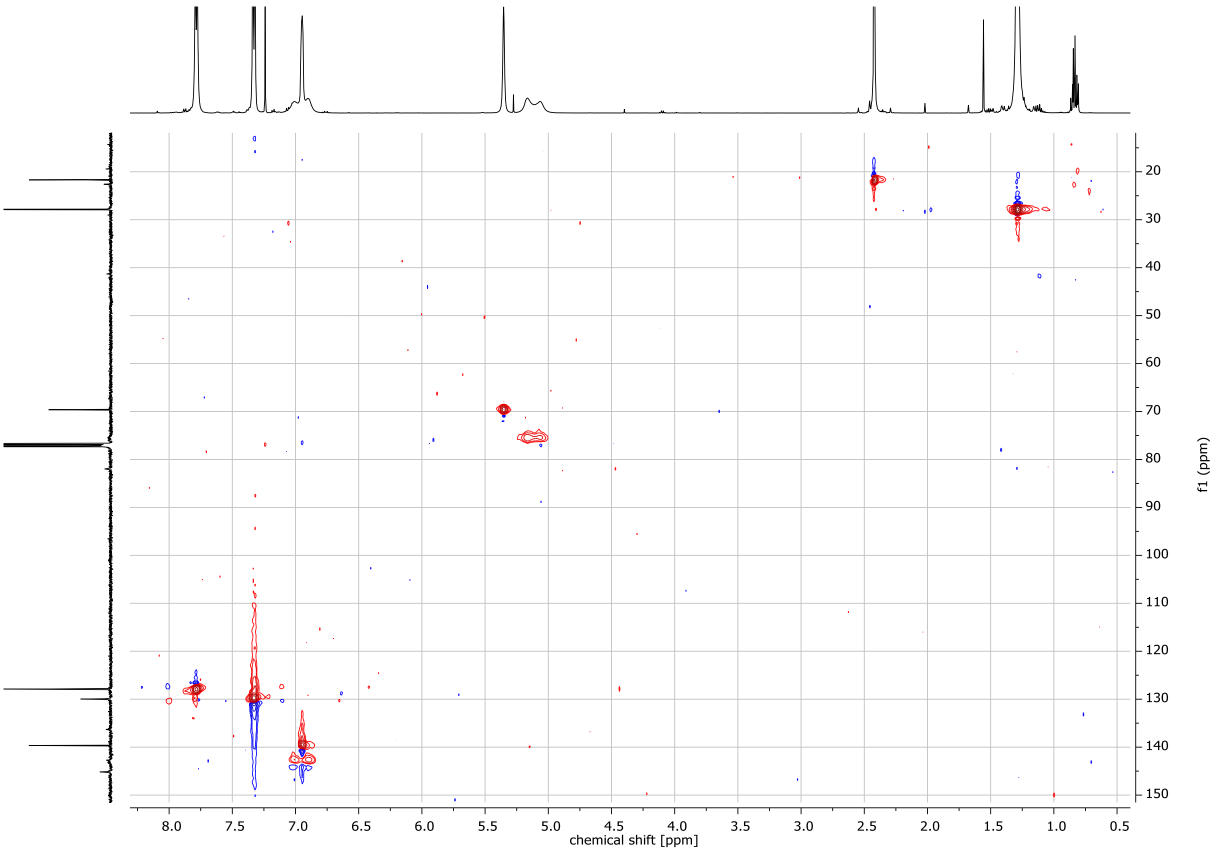

Figure S10: HSQC spectrum of ***N-NBD1*** *measured in in $CDCl_3$ (500 MHz, rt).*

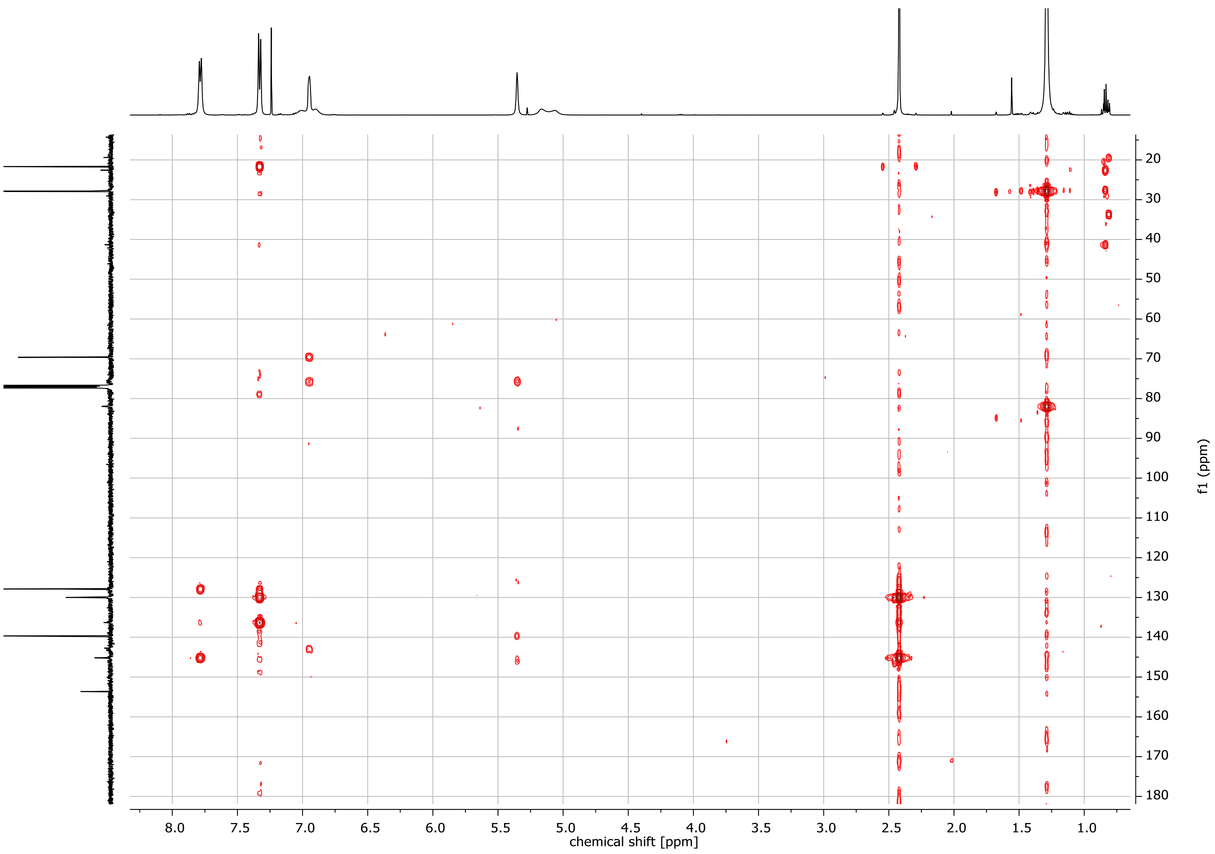

*Figure S11: HMBC spectrum of **N-NBD1** measured in in $CDCl_3$ (500 MHz, rt).*

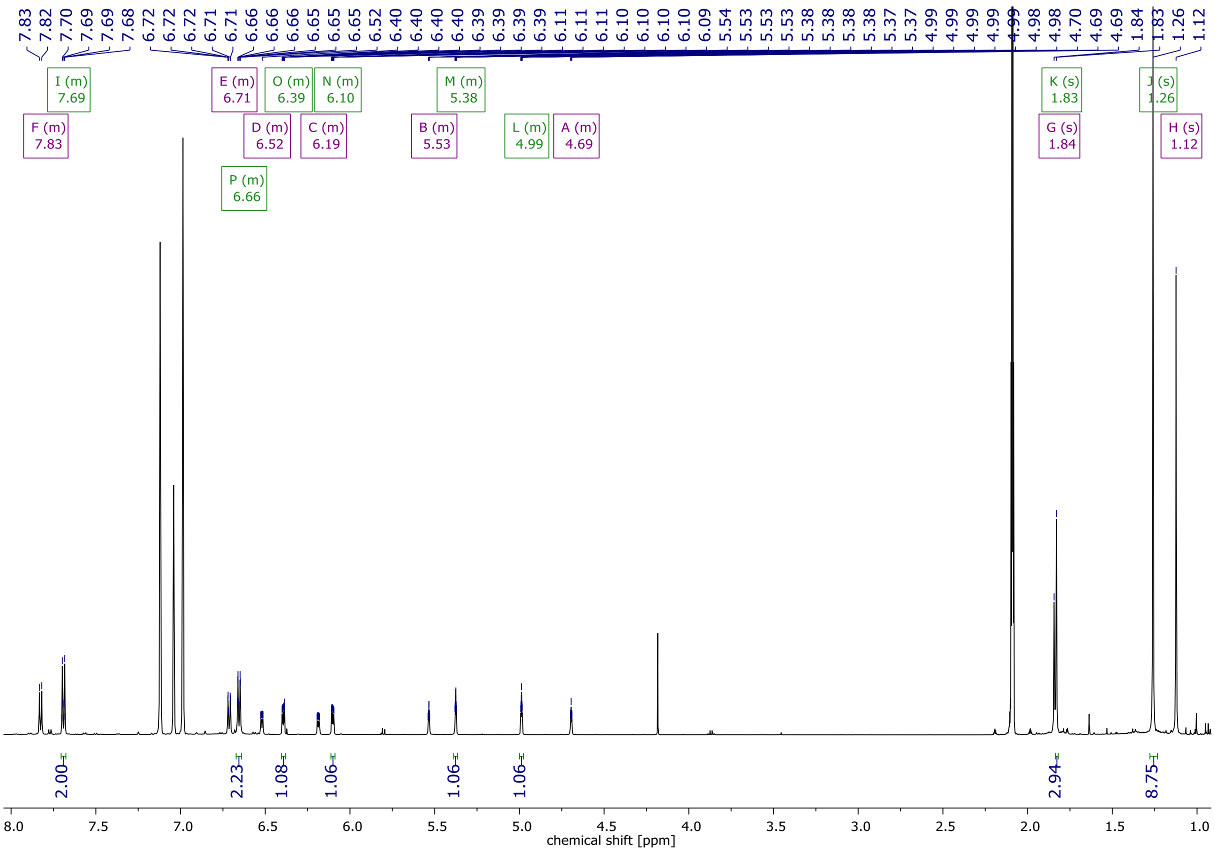

*Figure S12: Additional ^{1}H-NMR spectrum of **N-NBD1** measured in in toluene-d_8 (600 MHz, -10 °C). All signals picked in green belong to one conformer, all in purple to the other conformer.*

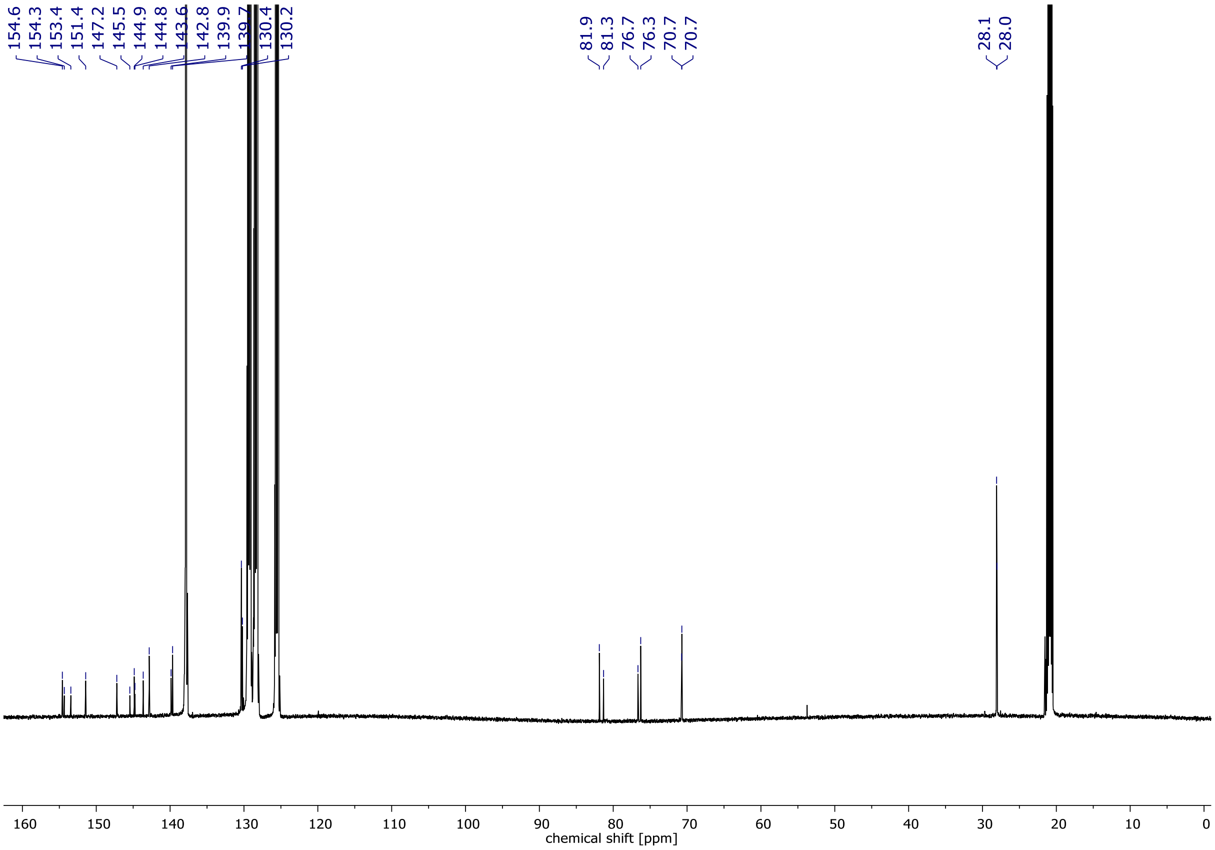

*Figure S13: Additional ^{13}C-NMR spectrum of **N-NBD1** measured in in toluene-d_8 (600 MHz, -10 °C). The picked signals belong to both conformers and were not separated.*

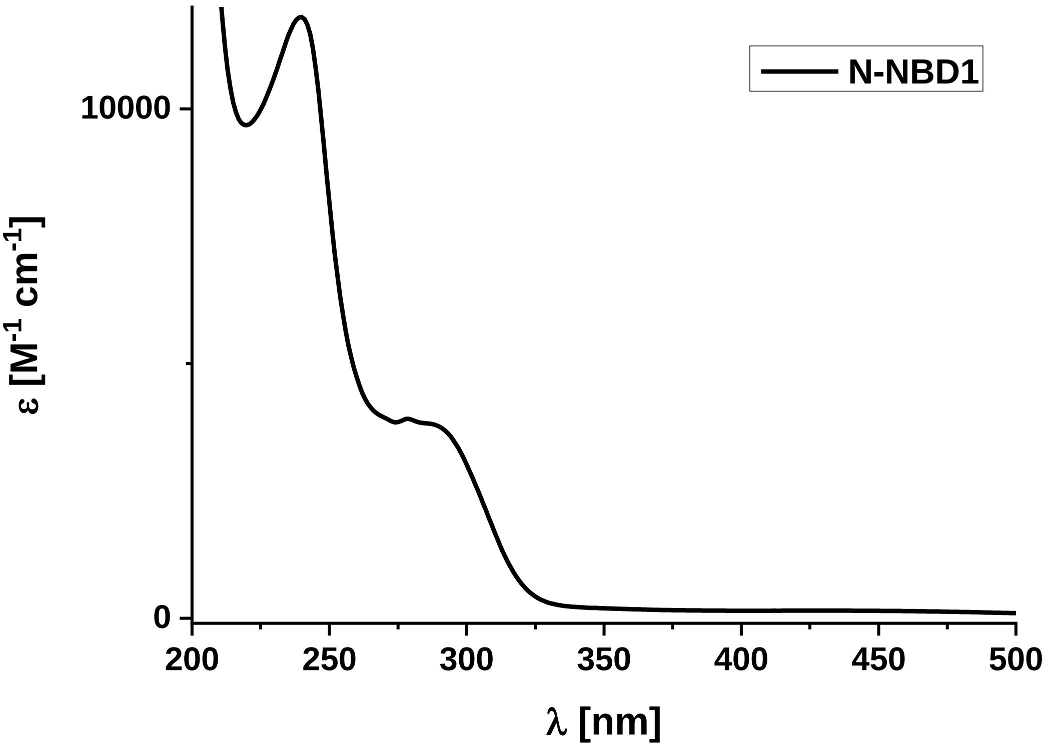

*Figure S14: UV–vis spectrum of **N-NBD1** measured in MeCN; ε (289) = 3800 $^{l}/_{mol*cm}$;, ε (240) = 11800 $^{l}/_{mol*cm}$.*

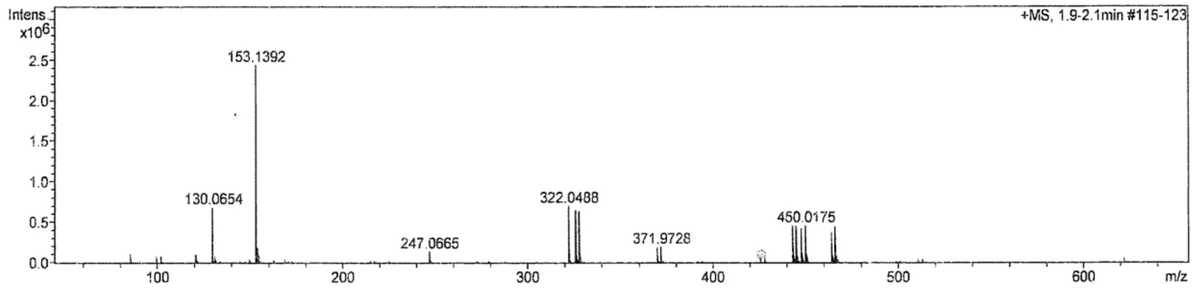

Meas. m/z	#	Ion Formula	m/z	err [ppm]	mSigma	# mSigma	Score	rdb	e⁻ Conf	N-Rule
426.0370	1	C18H21BrNO4S	426.0369	-0.1	63.9	1	100.00	8.5	even	ok

*Figure S15: HRMS (ESI) of **N-NBD1**.*

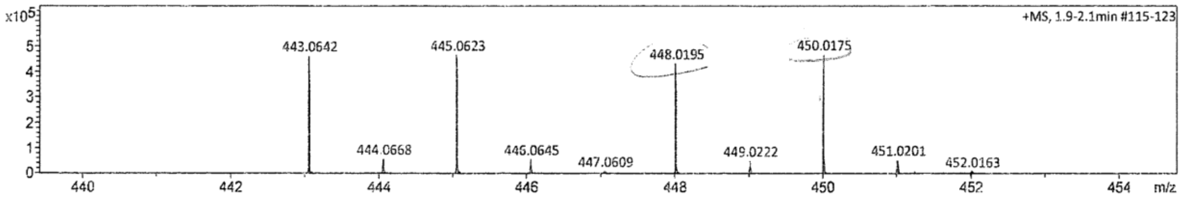

*Figure S16: Zoomed section of HRMS (ESI) of **N-NBD1** for $[M+Na]^+$.*

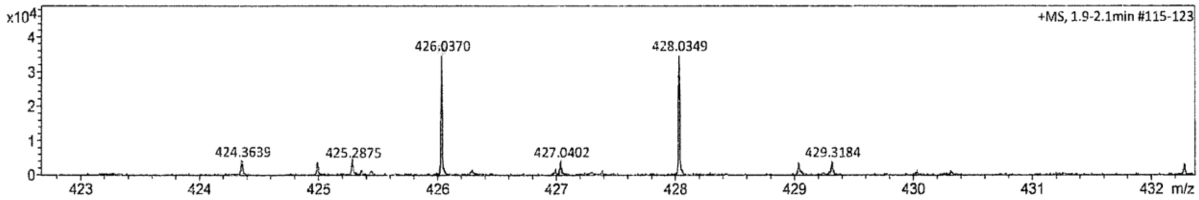

*Figure S17: Zoomed section of HRMS (ESI) of **N-NBD1** for $[M+H]^+$.*

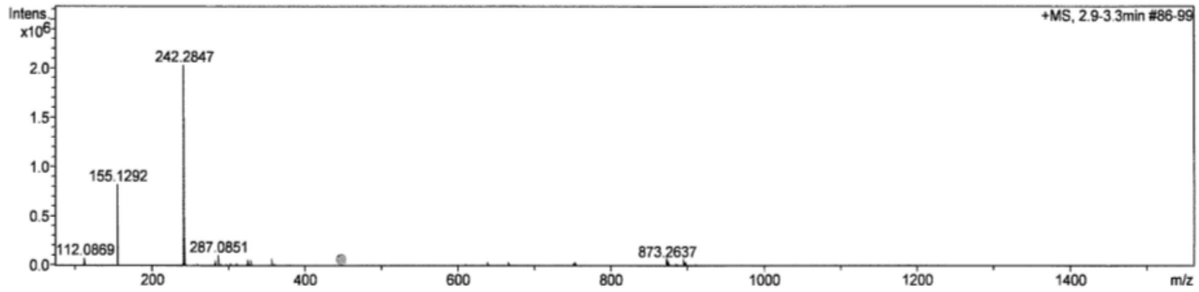

Meas. m/z	#	Ion Formula	m/z	err [ppm]	mSigma	# mSigma	Score	rdb	e⁻ Conf	N-Rule
448.0188	1	C18H20BrNNaO4S	448.0189	0.2	10.0	1	100.00	8.5	even	ok

*Figure S18: HRMS (APPI) of **N-NBD1.***

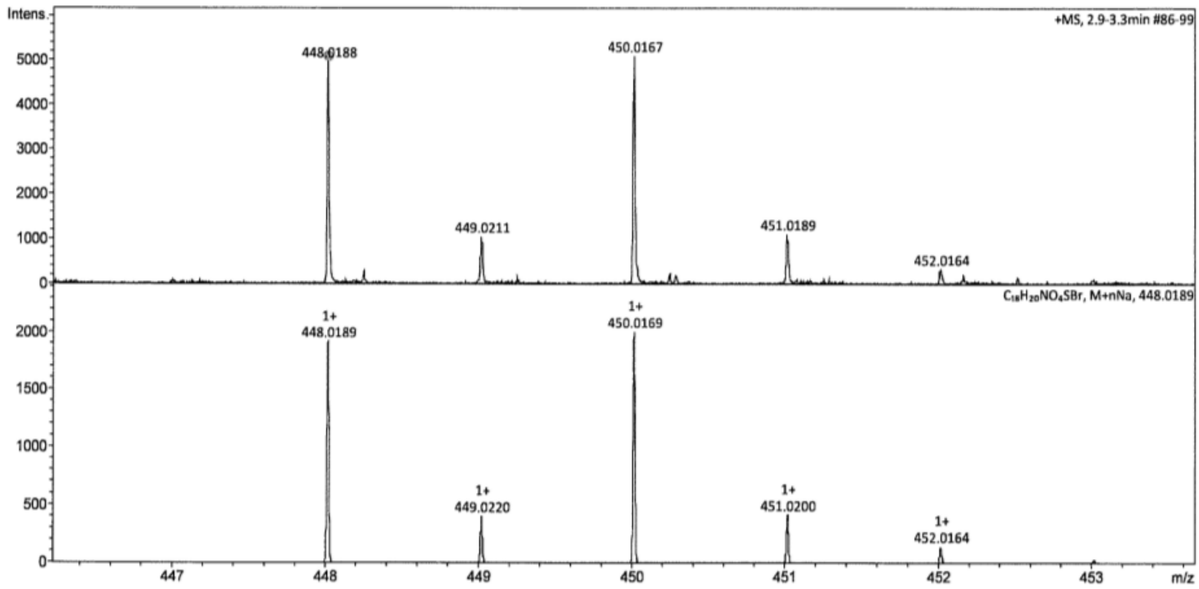

*Figure S19: Zoomed section of HRMS (APPI) of **N-NBD1** for [M+Na]+.*

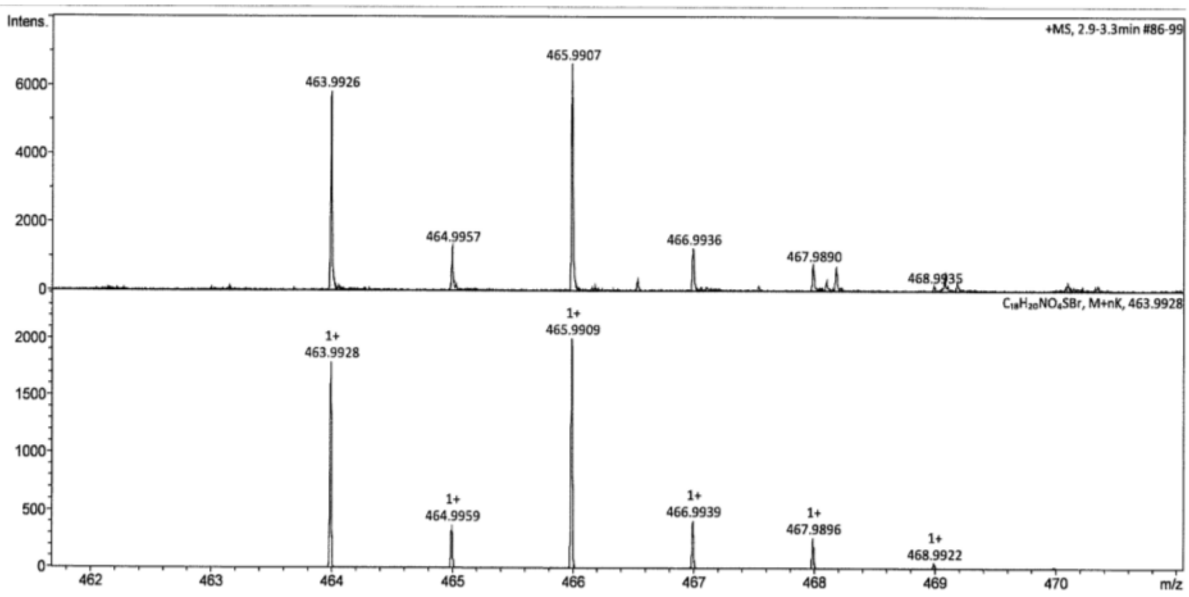

*Figure S20: Zoomed section of HRMS (APPI) of **N-NBD1** for [M+K]+.*

1.2.5 O-NBD2 (*N,N*-Diphenyl-4-((1*S*,4*R*)-3-tosyl-7-oxabicyclo[2.2.1]hepta-2,5-dien-2-yl)aniline)

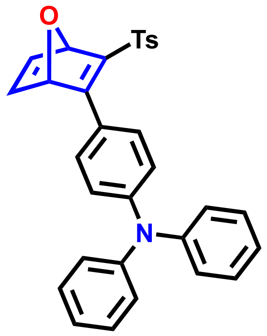

Chemical Formula: $C_{31}H_{25}NO_3S$
Molecular Weight: 491.60

According to general procedure A, 2-bromo-3-tosyl-7-oxabicyclo[2.2.1]hepta-2,5-diene (1.0 mmol, 327 mg, 1.0 equiv) was reacted with (4-(diphenylamino)phenyl)boronic acid (1.20 mmol, 347 mg, 1.2 equiv) at 80 °C for 16 h. Purification was achieve *via* automized flash column chromatography using a mixture of 25% EtOAc in hexanes as eluent yielding an orange solid.

Yield: 141 mg, 0.286 mmol, 29%.

$\mathbf{R_f}$ = 0.26 (hexanes/EtOAc 4:1)

^{1}H-NMR (400 MHz, $CDCl_3$, 25 °C) δ_H [ppm]: 7.69-7.65 (m, 2 H), 7.48-7.44 (m, 2 H), 7.33-7.22 (m, 5 H), 7.14-7.11 (m, 6 H), 7.10-7.04 (m, 3 H), 7.00-6.96 (m, 2 H), 5.70-5.59 (m, 1 H), 5.65-5.63 (m, 1 H), 2.42 (s, 3 H).

^{13}C-NMR (100 MHz, $CDCl_3$, 25 °C): (δ) [ppm] = 162.2, 150.1, 146.8, 144.4, 144.1, 142.8, 139.8, 137.5, 129.9, 129.8, 129.7, 127.5, 125.8, 124.5, 123.3, 120.6, 88.6, 85.8, 21.8.

UV–vis: λ_{max} (ε in M^{-1} cm^{-1}) = 392 nm (15600) nm, $\lambda_{\pi-\pi^*}$ = 295 nm (14600), λ_{onset} = 498 nm.

HRMS (APPI): calc. for ($C_{31}H_{26}NO_3S$): 492.1628; found *m/z* = 492.1622 $[M+H]^+$.

mp: 116.2-118.5 °C.

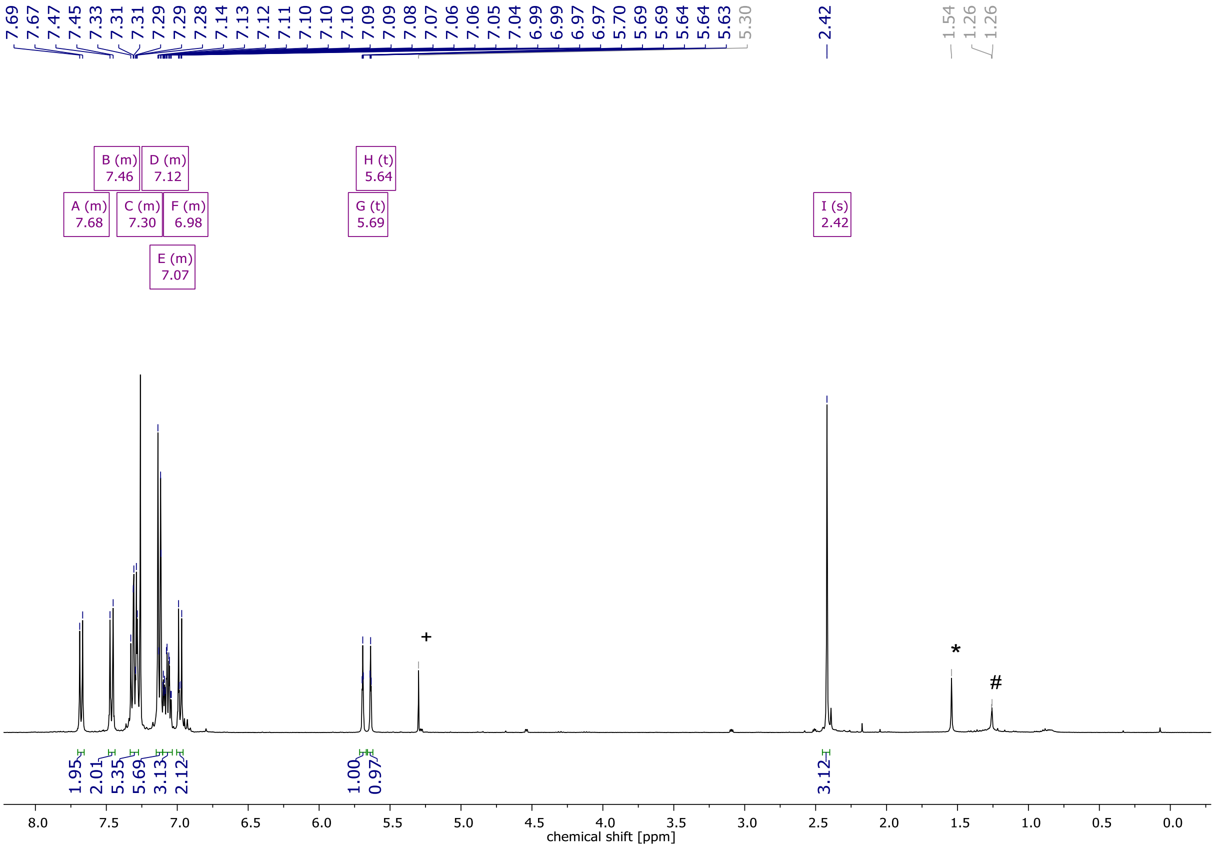

*Figure S21: ^{1}H-NMR spectrum of **O-NBD2** measured in in $CDCl_3$ (400 MHz, rt). The additional impurity signals found at 5.30 ppm (plus), 1.54 ppm (star) and 1.26 ppm (hashtag) can be assigned to methylene chloride, water and n-hexane, respectively. A very weak multiplet signal at 0.87-0.83 ppm can be assigned to H grease.*

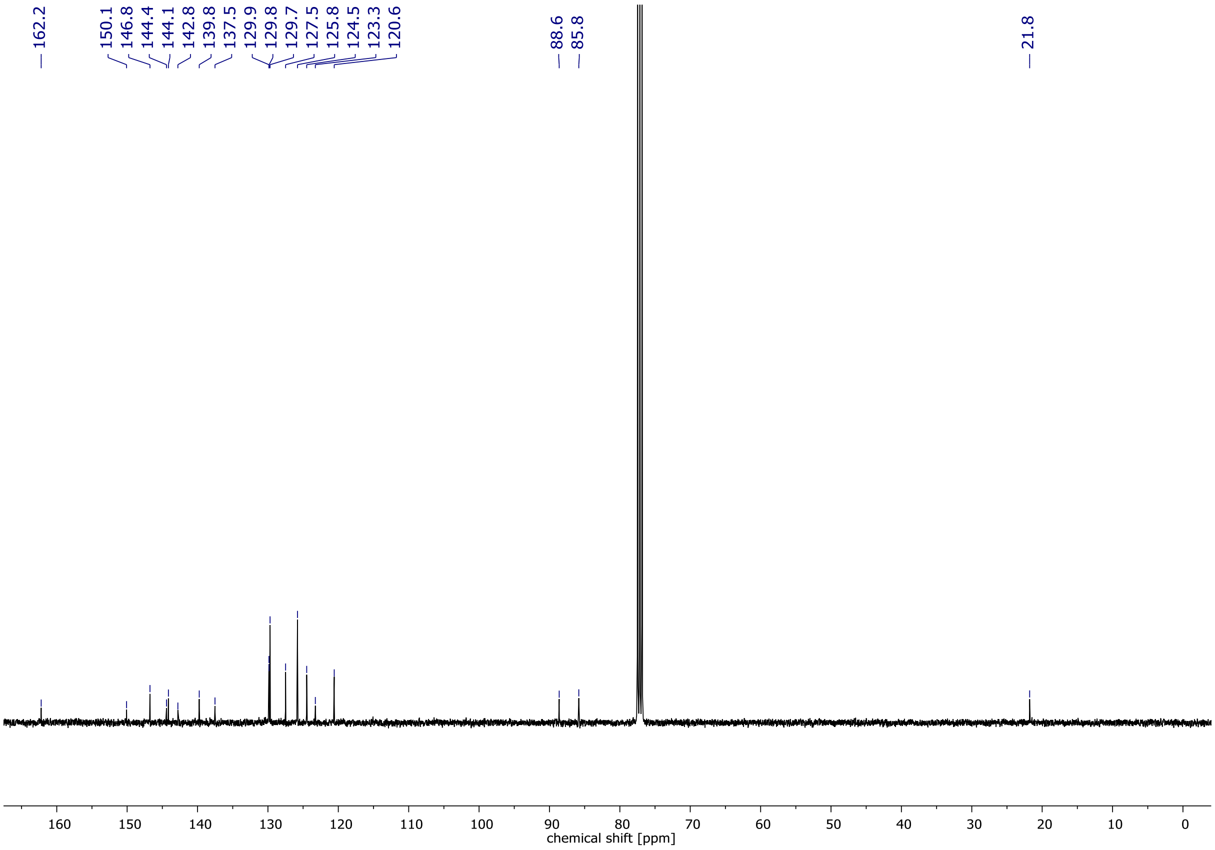

*Figure S22: ^{13}C-NMR spectrum of **O-NBD2** measured in in $CDCl_3$ (100 MHz, rt).*

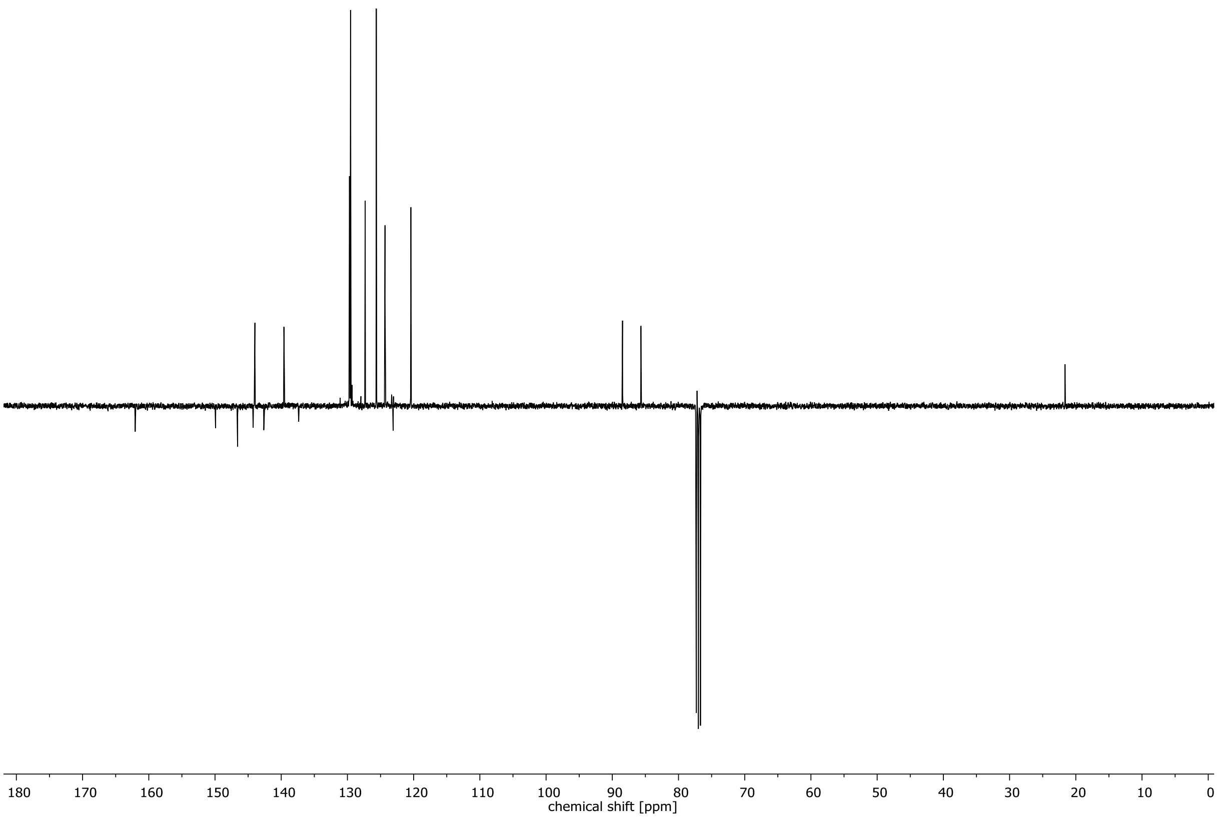

*Figure S23: DEPTq spectrum of **O-NBD2** measured in $CDCl_3$ (100 MHz, rt).*

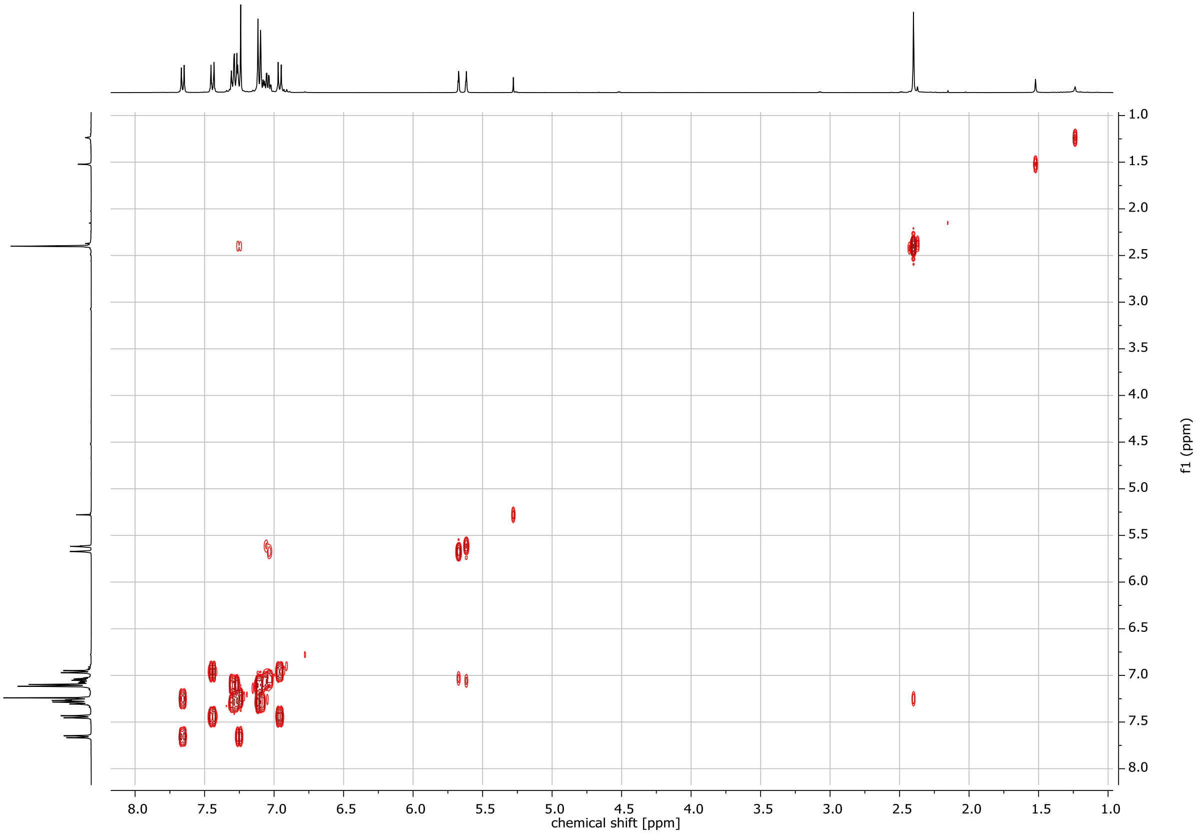

*Figure S24: COSY spectrum of **O-NBD2** measured in $CDCl_3$ (400 MHz, rt).*

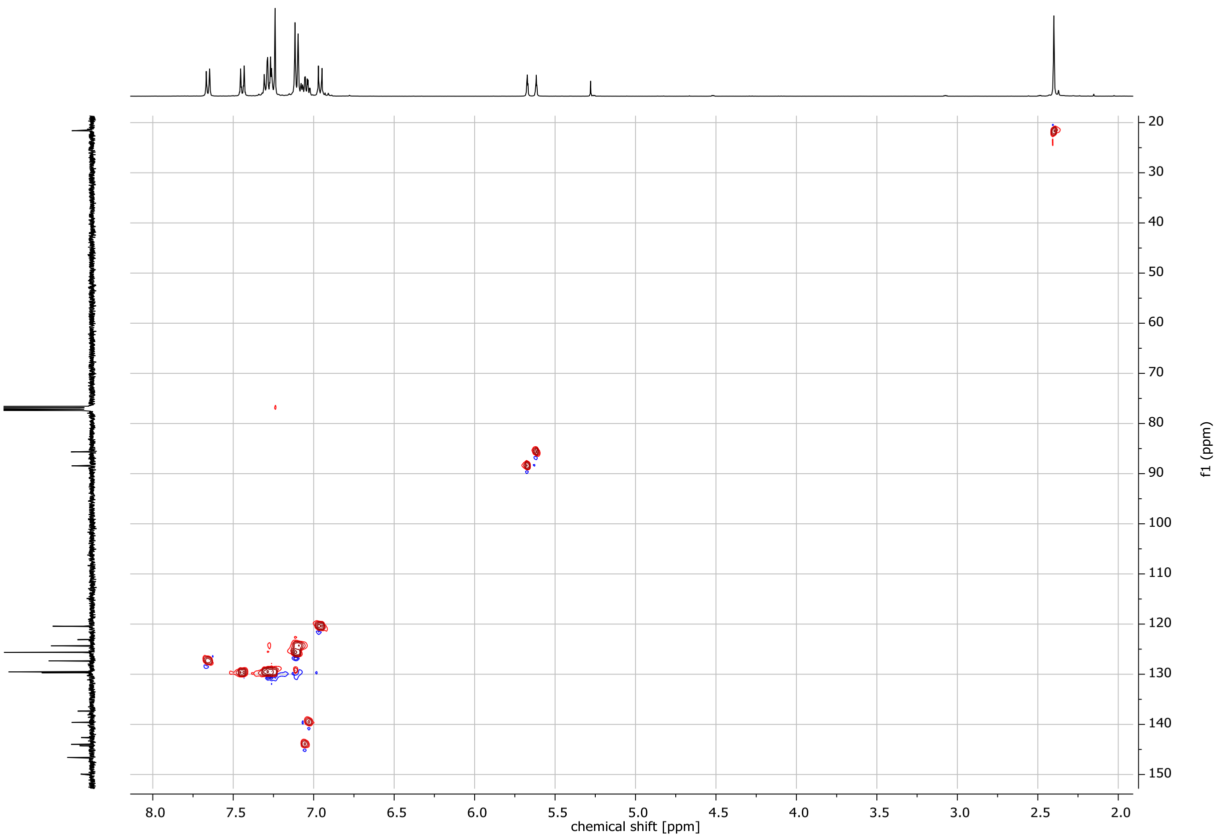

*Figure S25: HSQC spectrum of **O-NBD2** measured in $CDCl_3$ (400 MHz, rt).*

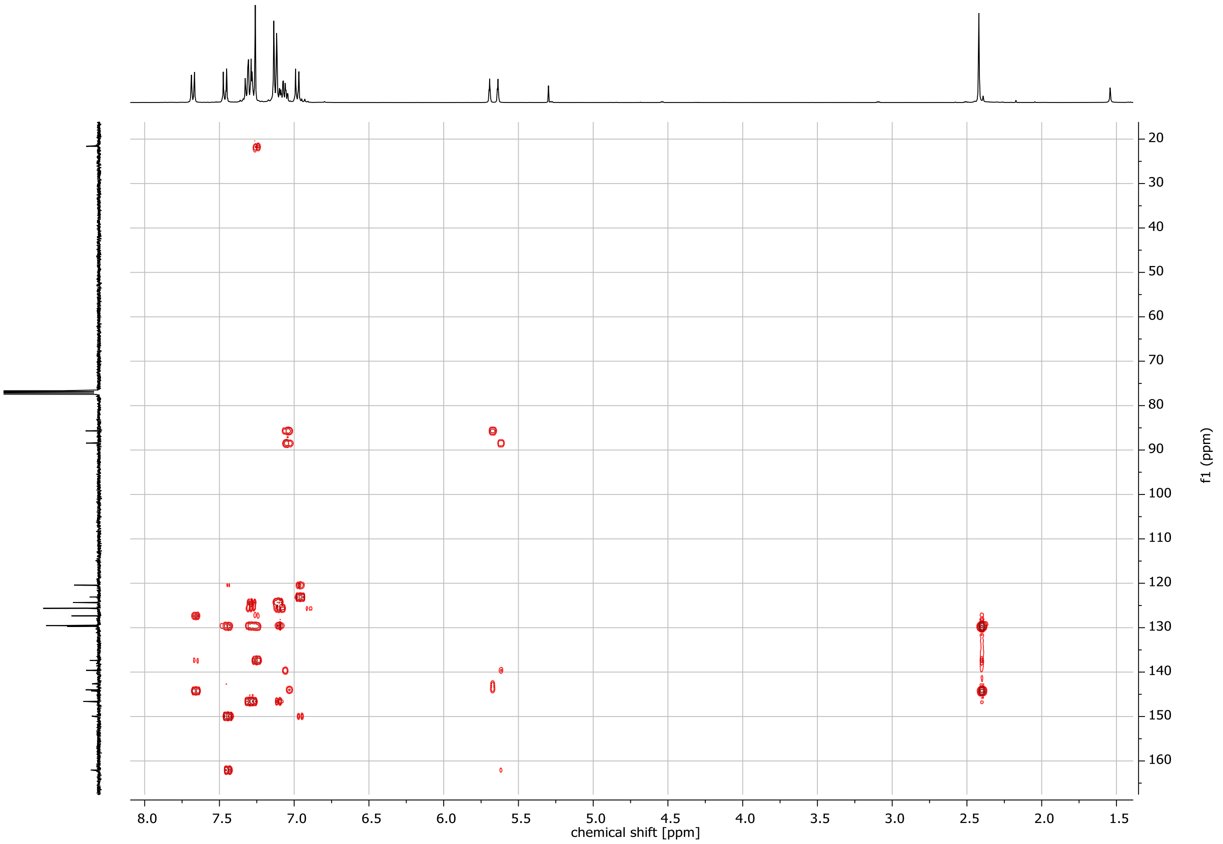

*Figure S26: HMBC spectrum of **O-NBD2** measured in $CDCl_3$ (400 MHz, rt).*

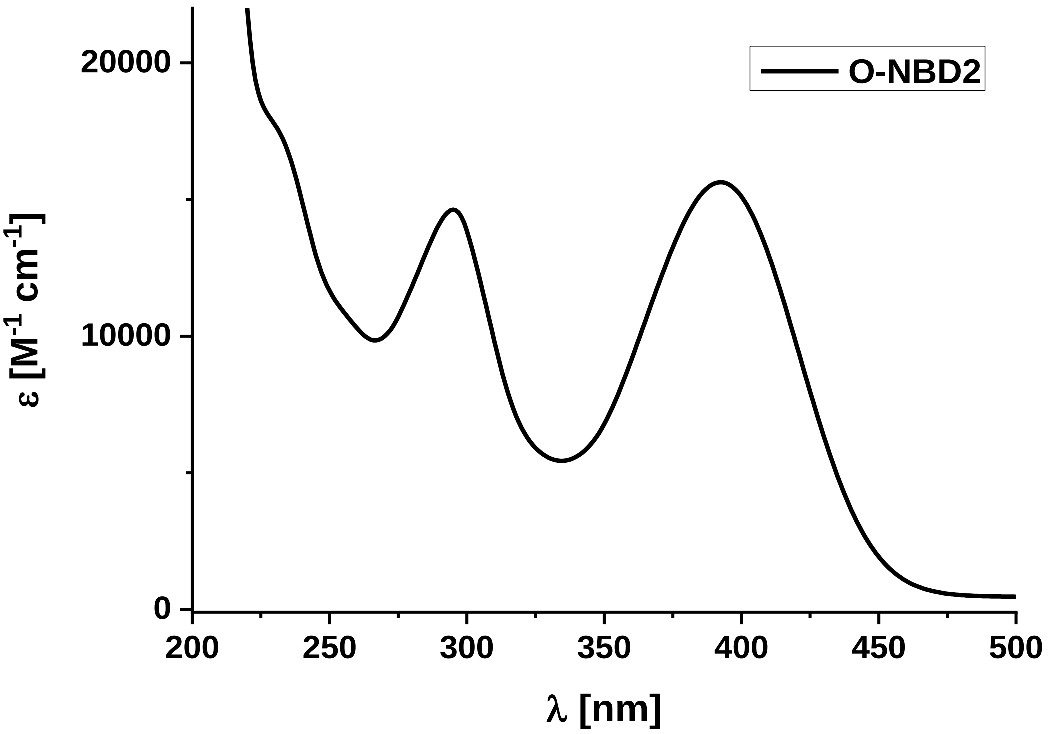

*Figure S27: UV–vis spectrum of **O-NBD2** measured in MeCN; ε (392 nm) = 15600 $^{l}/_{mol*cm}$;, ε (295 nm) = 14600 $^{l}/_{mol*cm}$.*

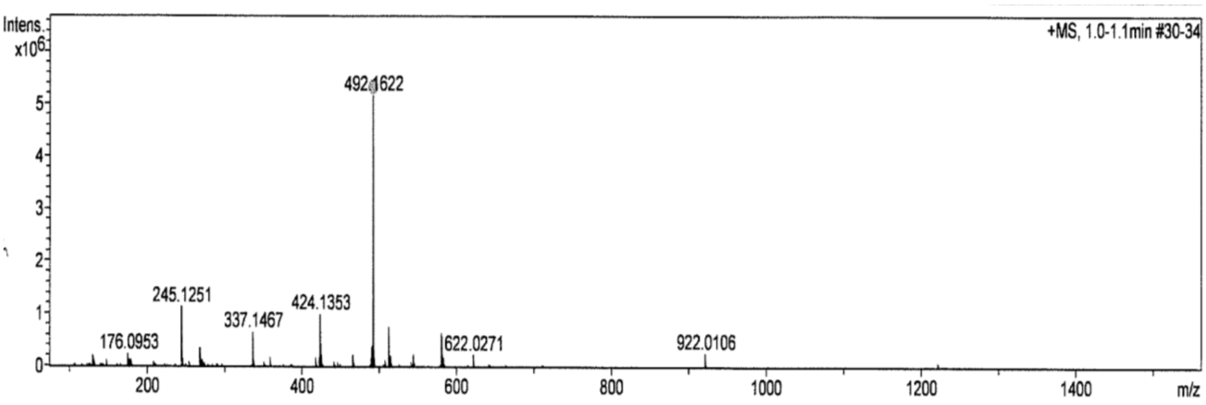

Meas. m/z	#	Ion Formula	m/z	err [ppm]	mSigma	# mSigma	Score	rdb	e⁻ Conf	N-Rule
492.1622	1	C31H26NO3S	492.1628	1.3	34.7	1	100.00	19.5	even	ok

*Figure S28: HRMS (APPI) of **O-NBD2**.*

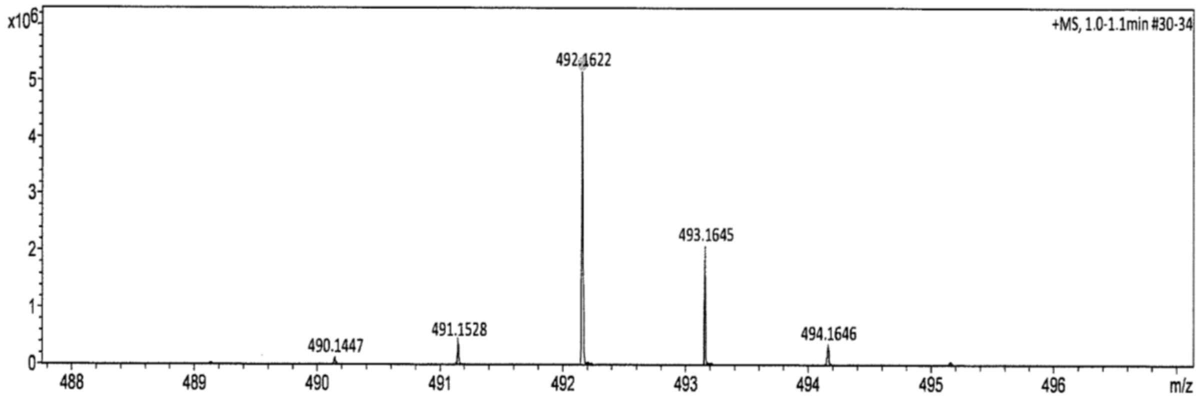

*Figure S29: Zoomed section of HRMS (APPI) of **O-NBD2**.*

1.2.6 N-NBD2 (*tert*-Butyl (1*S*,4*R*)-2-(4-(diphenylamino)phenyl)-3-tosyl-7-azabicyclo[2.2.1]hepta-2,5-diene-7-carboxylate)

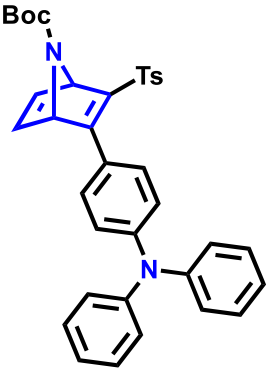

Chemical Formula: $C_{36}H_{34}N_2O_4S$
Molecular Weight: 590.74

According to general procedure A, *tert*-butyl-2-bromo-3-tosyl-7-azabicyclo[2.2.1]hepta-2,5-diene-7-carboxylate (1.23 mmol, 523 mg, 1.0 equiv) was reacted with (4-(diphenylamino)phenyl)boronic acid (1.48 mmol, 427 mg, 1.2 equiv) at 80 °C for 15 h. Purification was achieve *via* automized flash column chromatography using a mixture of 25% EtOAc in hexanes as eluent yielding a yellow semi-solid.

Yield: 200 mg, 0.339 mmol, 26%.

R_f = 0.36 (hexanes/EtOAc 4:1)

^{1}H-NMR (400 MHz, $CDCl_3$, 25 °C) δ_H [ppm]: 7.72-7.62 (m, 2 H), 7.46-7.39 (m, 2 H), 7.33-7.24 (m, 6 H), 7.15-7.10 (m, 6 H), 7.00-6.93 (m, 4 H), 5.51-5.34 (m, 2 H), 2.41 (s, 3 H), 1.35 (broad s, 9 H)

^{13}C-NMR (100 MHz, $CDCl_3$, 25 °C): (δ) [ppm] = 149.9, 146.9, 144.3, 143.4, 139.5, 137.5, 129.7, 129.6, 127.7, 125.7, 124.3, 120.7, 73.1, 81.6, 70.0, 28.1, 21.8.

UV–vis: λ_{max} (ε in M^{-1} cm^{-1}) = 393 nm (17300) nm, $\lambda_{\pi-\pi^*}$ = 294 nm (14200), λ_{onset} = 479 nm.

HRMS (APPI): calc. for ($C_{36}H_{35}N_2O_4S$): 591.2312; found *m/z* = 591.2318 $[M+H]^+$. calc. for ($C_{32}H_{27}N_2O_4S^+$): 535.1687; found *m/z* = 535.1696 $[M+H-C_4H_9]^+$. *Molecule without tert-butyl group*

mp: 68.4-69.1 °C.

Due to the possible inversion of the nitrogen atom, additional NMR spectroscopy was recorded at low temperatures to generate a splitting of the two signal sets.

^{1}H-NMR (main isomer, green) (600 MHz, $CDCl_3$, -10 °C) δ_H [ppm]: 7.63-7.60 (m, 2H), 7.51-7.48 (m, 2H), 6.64 (ddd, *J* = 5.4, 2.5, 0.7 Hz, 1H), 6.61-6.59 (m, 2H), 6.42 (ddd, *J* = 5.4, 2.6, 0.8 Hz, 1H), 5.64-5.63 (m, 1H), 5.50-5.49 (m, 1H), 1.82 (s, 4H)*, 1.37 (s, 9H).

All aromatic signals were not picked due to massive overlapping of both conformers making integration and clean assignment impossible.

*the signals for both tosyl groups overlap thus disturbing the integration.

^{1}H-NMR (side isomer, purple) (600 MHz, $CDCl_3$, -10 °C) δ_H [ppm]: 7.78-7.75 (m, 2H), 7.54-7.51 (m, 2H), 6.76 (dd, *J* = 5.3, 2.5 Hz, 1H), 6.67-6.65 (m, 1H), 6.46 (ddd, *J* = 5.4, 2.5, 0.8 Hz, 1H), 6.42 (ddd, *J* = 5.4, 2.6, 0.8 Hz, 1H), 5.78-5.76 (m, 1H), 5.30-5.28 (m, 1H), 1.82 (s, 3H), 1.24 (s, 9H).

^{13}C-NMR (151 MHz, toluene-d_8, -10 °C): (δ) [ppm] = 162.8, 161.4, 154.5, 153.9, 149.6, 149.5, 147.0, 147.0, 145.6, 144.0, 143.9, 143.3, 143.2, 142.8, 139.1, 138.9, 138.2, 137.9, 137.0, 137.0, 130.3, 130.2, 129.5, 129.5, 129.3, 129.3, 125.5, 125.4, 124.5, 124.0, 123.9, 120.8, 120.7, 80.7, 80.1, 73.4, 73.4, 70.4, 70.4, 53.1, 27.6.

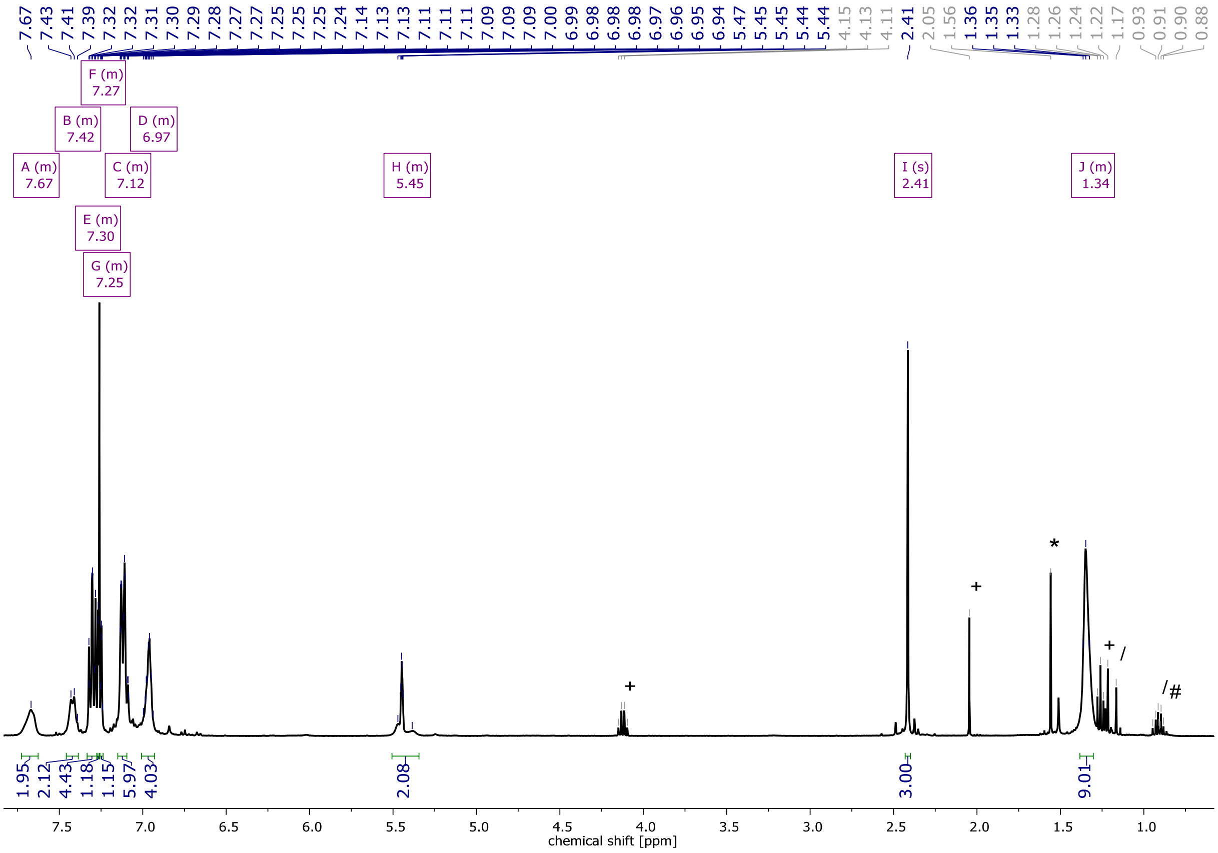

Figure S30: ^{1}H-NMR spectrum of ***N-NBD2*** *measured in in $CDCl_3$ (400 MHz, rt). The additional impurity signals can be assigned as follows: +) quartet at 4.12 ppm, singlet at 2.05 and triplet at 1.25 ppm belong to EtOAc, *) at 1.56 ppm belongs to water, /) at 1.26 and 0.88 ppm belongs to n-hexane, #) at 0.87-0.83 ppm can be assigned H grease, respectively.*

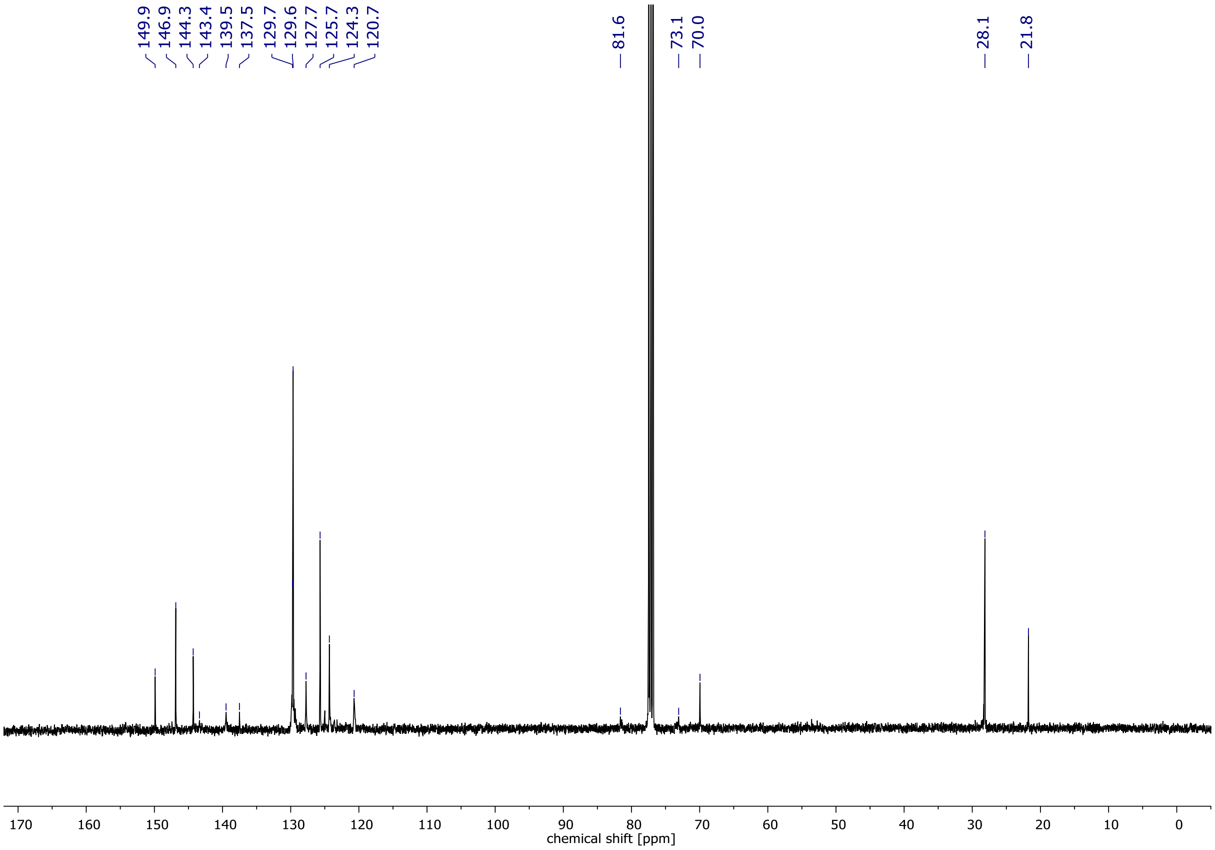

*Figure S31: ^{13}C-NMR spectrum of **N-NBD2** measured in in $CDCl_3$ (100 MHz, rt).*

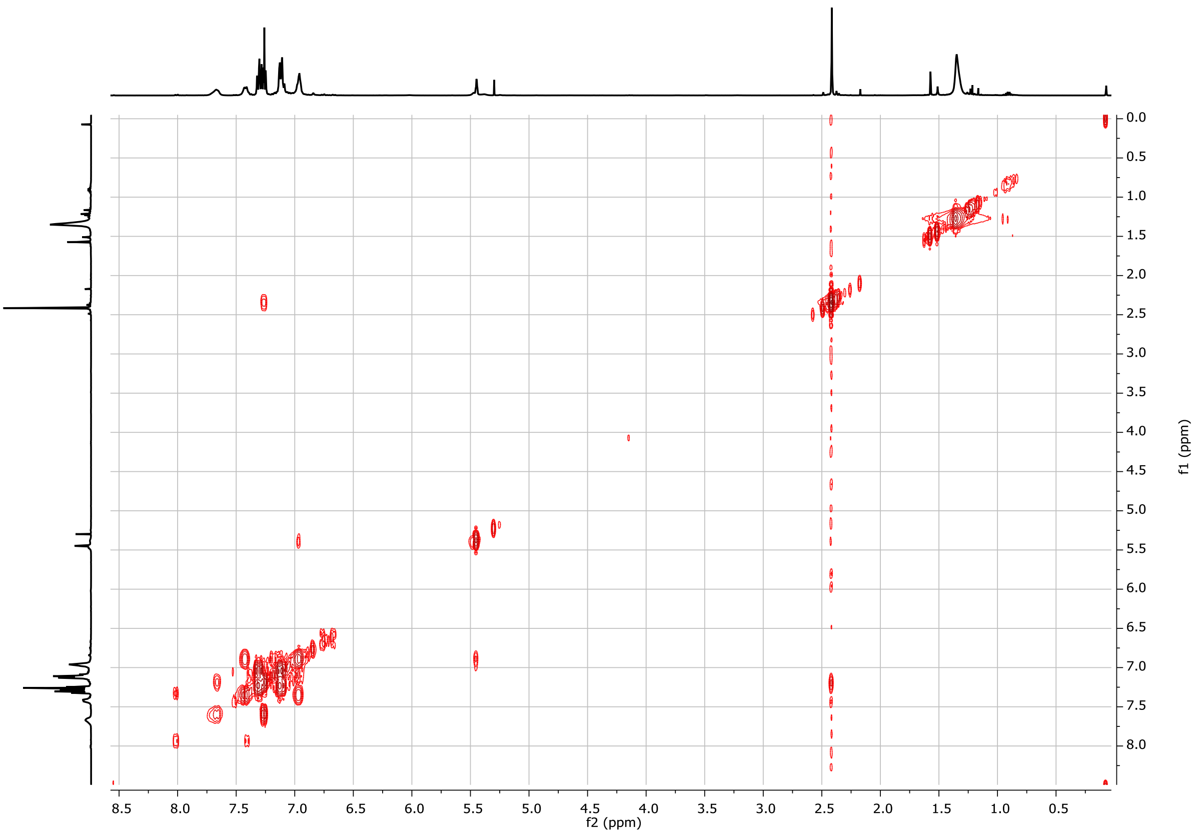

*Figure S32: COSY spectrum of **N-NBD2** measured in $CDCl_3$ (400 MHz, rt).*

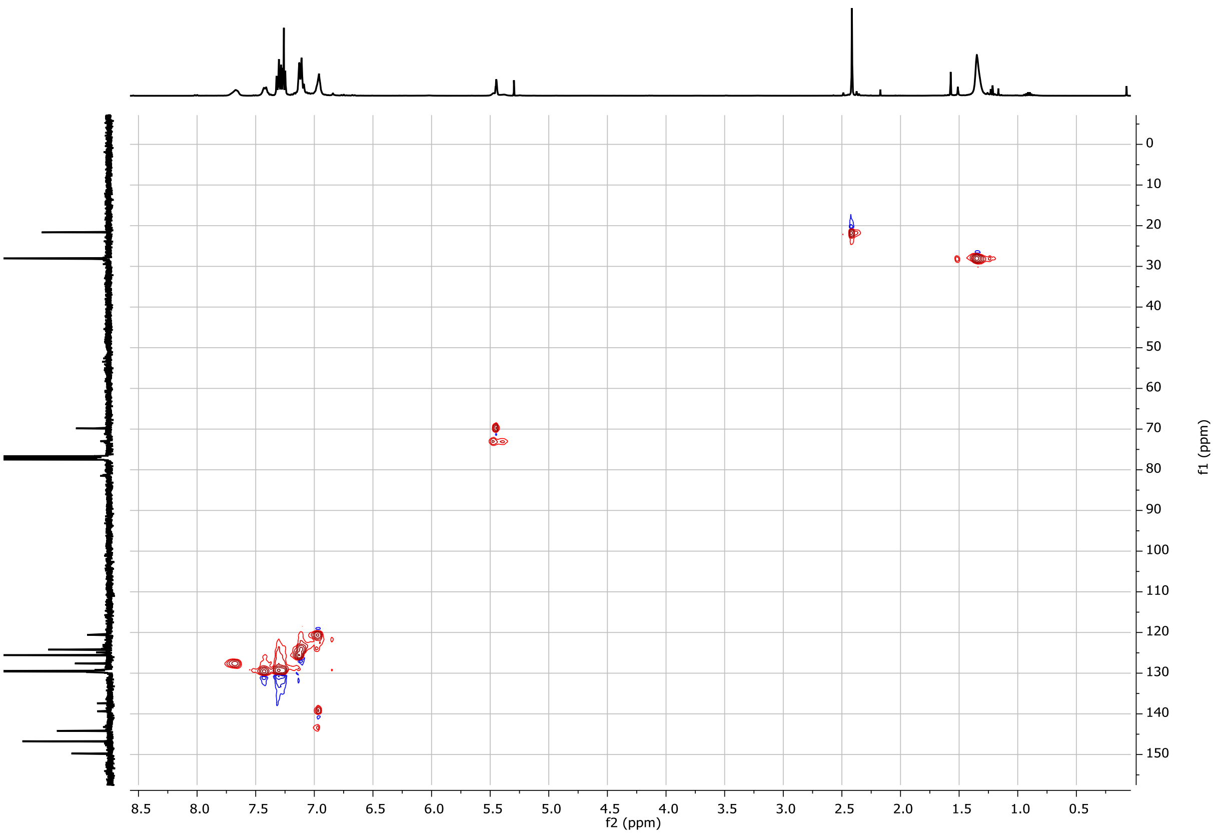

*Figure S33: HSQC spectrum of **N-NBD2** measured in $CDCl_3$ (400 MHz, rt).*

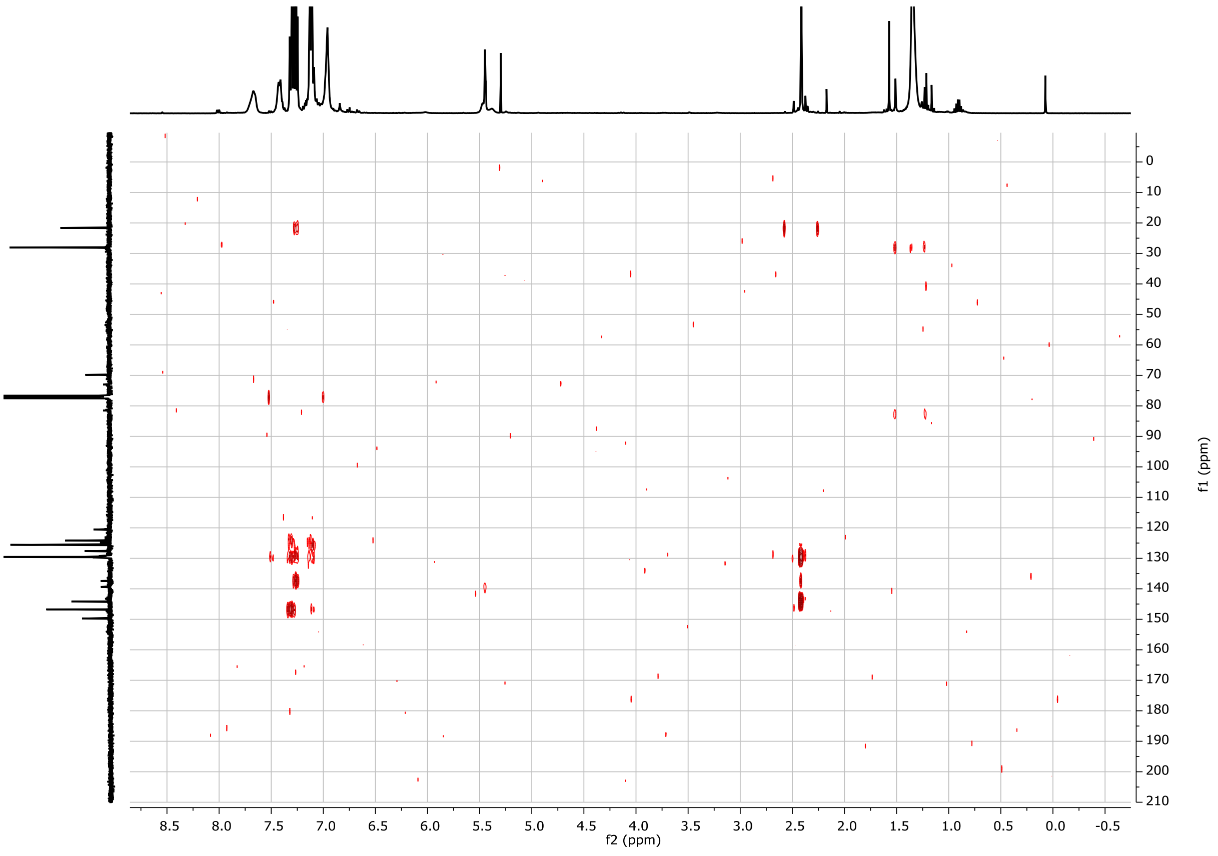

*Figure S34: HMBC spectrum of **N-NBD2** measured in $CDCl_3$ (400 MHz, rt).*

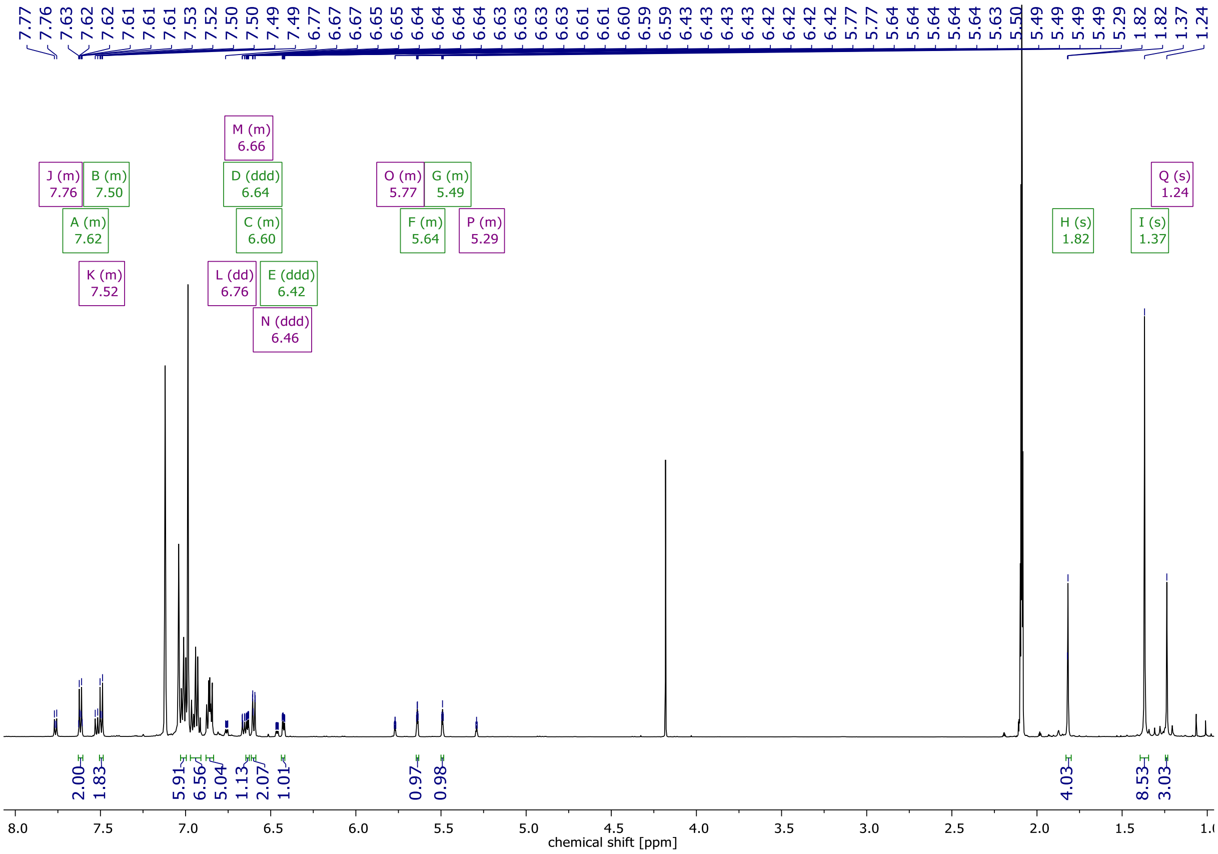

Figure S35: ^{1}H-NMR spectrum of ***N-NBD2*** *measured in in toluene-d_8 (600 MHz, -10 °C).*

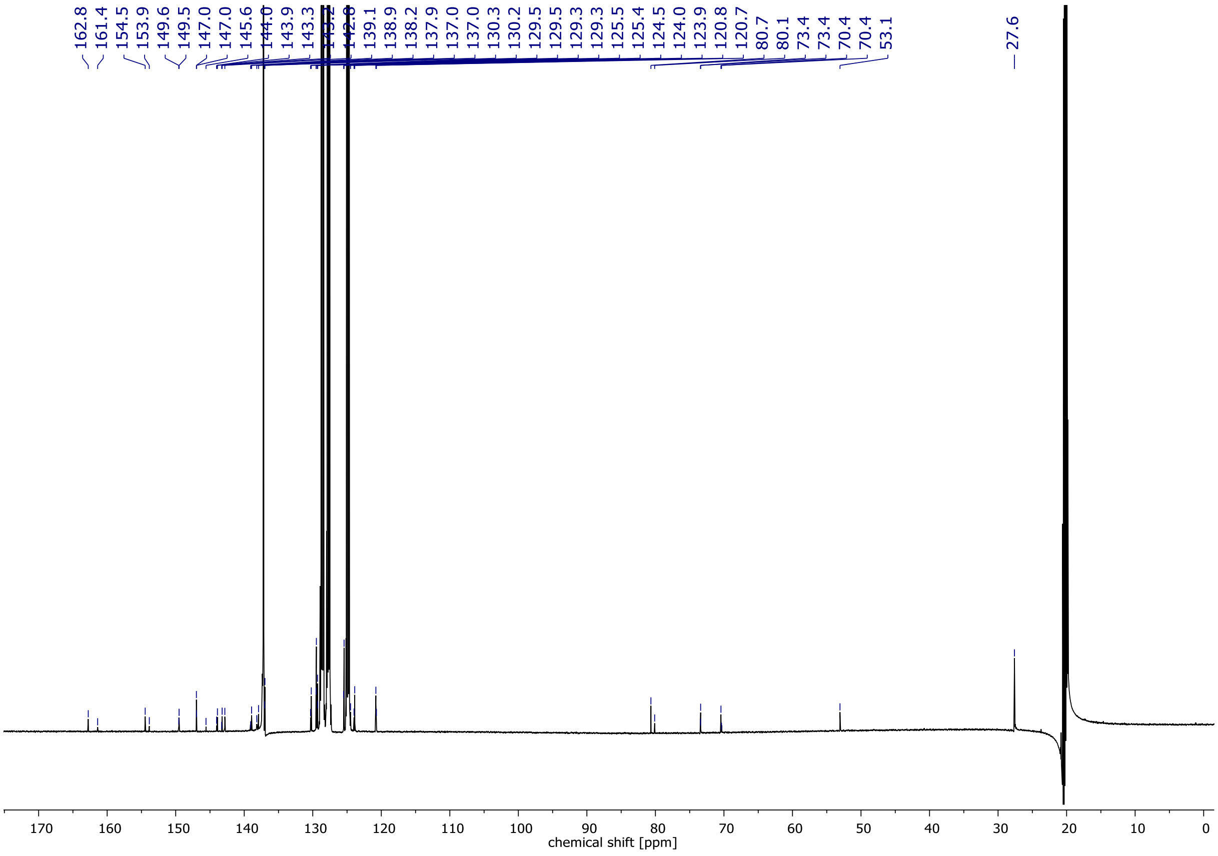

Figure S36: ^{13}C-NMR spectrum of ***N-NBD2*** *measured in in toluene-d_8 (600 MHz, -10 °C).*

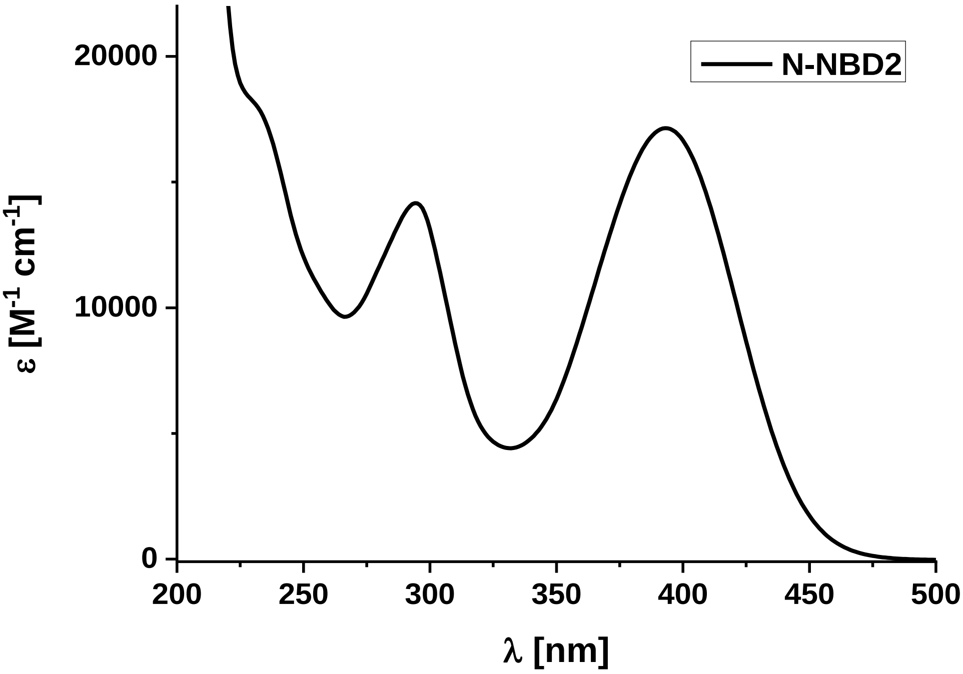

Figure S37: UV–vis spectrum of ***N-NBD2*** *measured in MeCN; ε (393 nm) = 17300 $^{l}/_{mol*cm}$;, ε (294 nm) = 14200 $^{l}/_{mol*cm}$.*

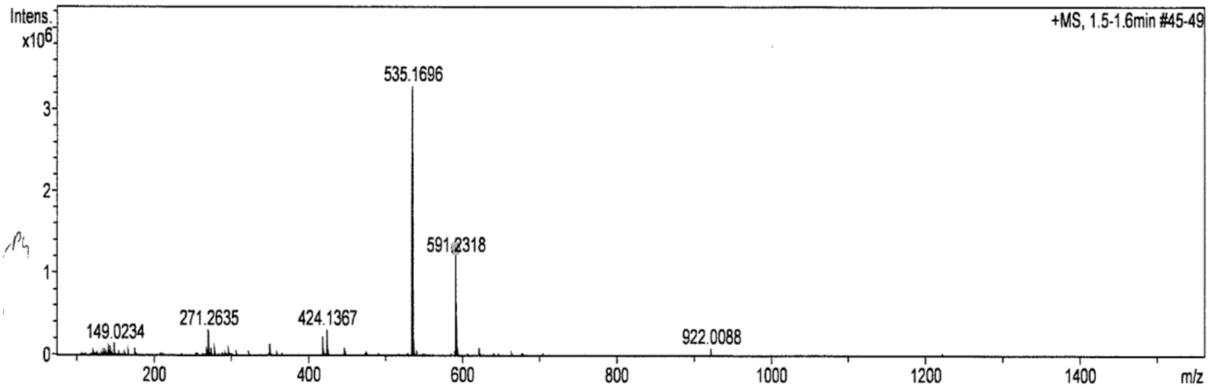

Meas. m/z	#	Ion Formula	m/z	err [ppm]	mSigma	# mSigma	Score	rdb	e⁻ Conf	N-Rule
591.2318	1	C36H35N2O4S	591.2312	-1.0	40.7	1	100.00	20.5	even	ok

Figure S38: of HRMS (APPI) of ***N-NBD2****.*

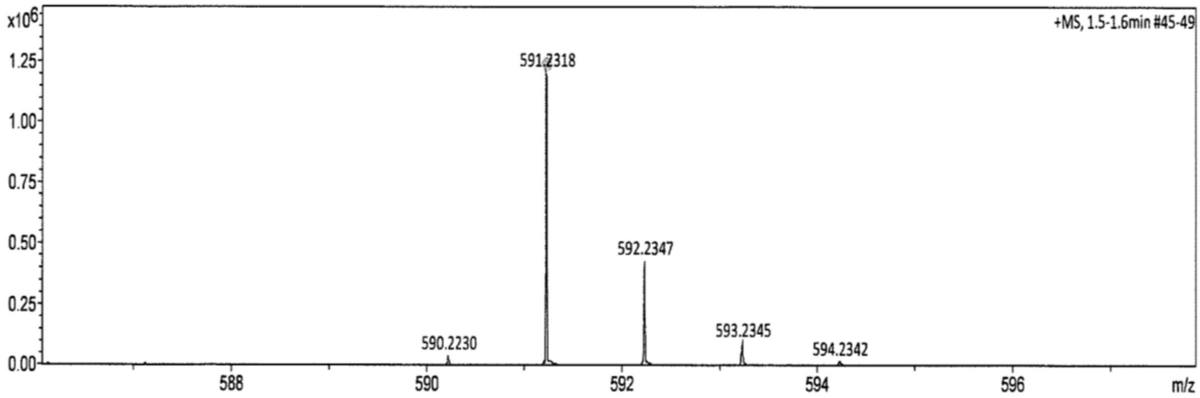

Figure S39: Zoomed section of HRMS (APPI) of ***N-NBD2****.*

1.2.7 **C-QC1** (1-Bromo-5-tosyltetracyclo[3.2.0.0^{2,7}.0^{4,6}]heptane)

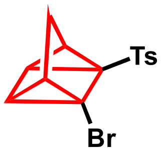

Chemical Formula: $C_{14}H_{13}BrO_2S$
Molecular Weight: 325.22

Switching of **C-NBD1** to **C-QC1** was carried out according to literature. Complete characterization, the detailed photophysical investigation and the measured spectroscopic features of **C-QC1** are in agreement with the reported analysis published before [3].

1.2.8 **O-QC1** (1-Bromo-5-tosyl-3-oxatetracyclo[3.2.0.0^{2,7}.0^{4,6}]heptane)

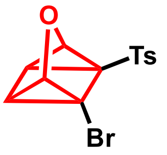

Chemical Formula: $C_{13}H_{11}BrO_3S$
Molecular Weight: 327.19

Switching of **O-NBD1** was conducted according to general procedure B. **O-NBD1** (6.5 mg) was dissolved in $CDCl_3$ and irradiated at 275 nm. Quantitative conversion to **O-QC1** was achieved after 240 min in NMR scale. In UV–vis scale (general procedure C), full isomerization to **O-QC1** was obtained after 300 seconds accompanied by slight indication of photodecomposition.

Conversion: quant. (240 min, $CDCl_3$).

^{1}H-NMR (400 MHz, $CDCl_3$, rt) δ_H [ppm]: 7.86-7.83 (m, 2H), 7.39-7.35 (m, 2H), 5.09 (dd, *J* = 4.0, 1.6 Hz, 1H), 4.65 (dd, *J* = 4.4, 1.6 Hz, 1H), 3.23 (t, *J* = 3.7 Hz, 1H), 2.65 (dd, *J* = 4.4, 3.4 Hz, 1H), 2.47 (s, 3H).

^{13}C-NMR (100 MHz, $CDCl_3$, rt): (δ) [ppm] = 145.0, 136.7, 129.9, 128.4, 71.4, 70.0, 47.2, 29.6, 27.9, 25.1, 21.7.

UV–vis: λ_{max}= 229 nm.

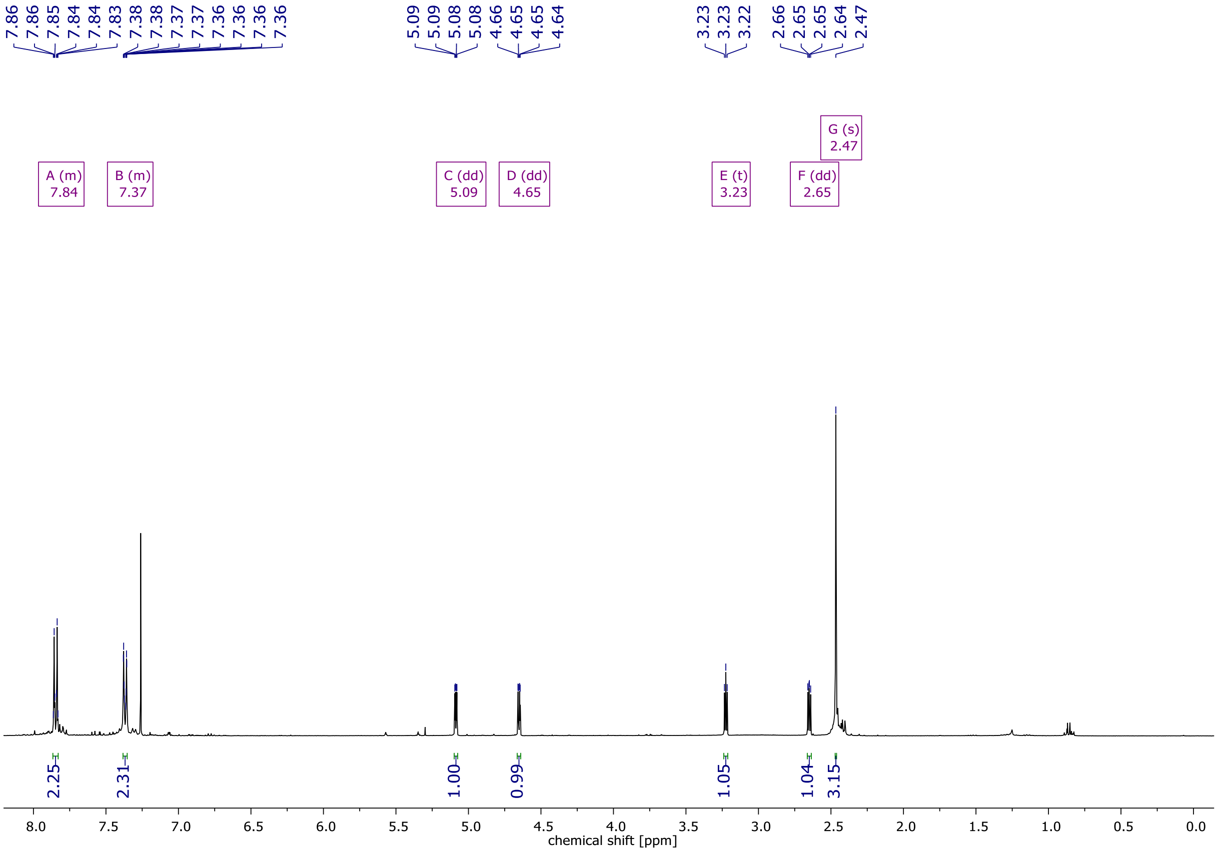

*Figure S40: ^{1}H-NMR spectrum of **O-QC1** measured in in $CDCl_3$ (400 MHz, rt).*

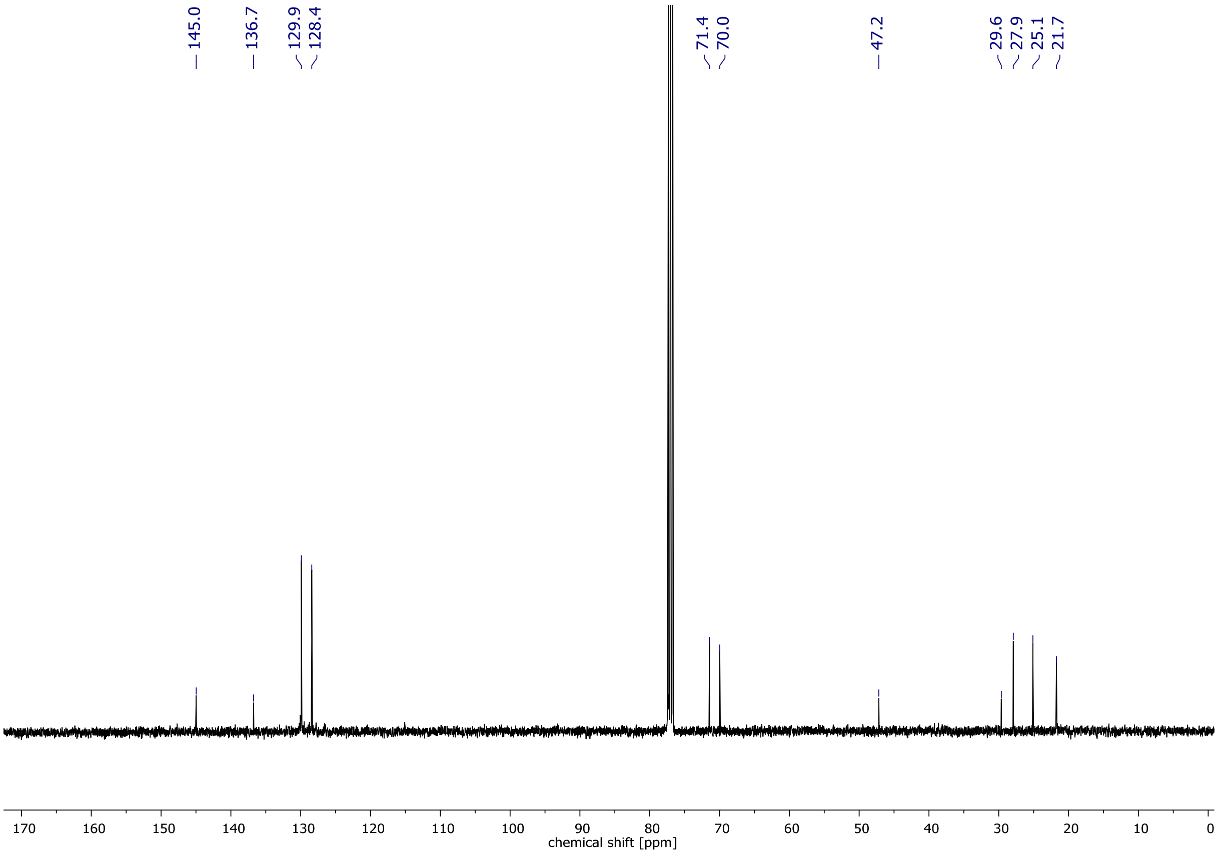

*Figure S41: ^{13}C-NMR spectrum of **O-QC1** measured in in $CDCl_3$ (100 MHz, rt).*

1.2.9 **N-QC1** (*tert*-Butyl 1-bromo-5-tosyl-3-azatetracyclo[3.2.0.0^{2,7}.0^{4,6}]heptane-3-carboxylate)

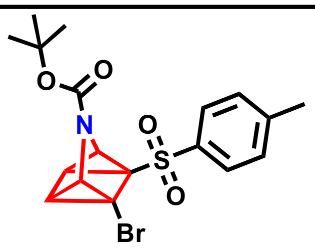

Chemical Formula: $C_{18}H_{20}BrNO_4S$
Molecular Weight: 426.33

Conversion of **N-NBD1** to **N-QC1** could not satisfy be proven. Switching of **N-NBD1** was conducted according to general procedure B. **N-NBD1** (6.5 mg) was dissolved in $CDCl_3$ and irradiated at 275 nm. Quantitative conversion to a new unknown species **N-UnS1** was achieved after 240 min in NMR scale. In UV–vis scale (general procedure C), consumption of **N-NBD1** was finished after 300 seconds accompanied by slight indication of photodecomposition. Prolonged irradiation led to complete photodecomposition. Since the addition of **POR** did not result in the regeneration of **N-NBD1**, formation of **N-QC1** is most likely excluded. The found spectroscopic features fit best for the formation an azepine derivative [10] while confirmation of such structure was not possible with exhaustive mass and NMR analysis.

Conversion: formation of a rearranged species accompanied by photodecomposition.

UV–vis: λ = 346 nm, λ = 218 nm.

1.2.10 **O-QC2** (*N,N*-Diphenyl-4-(5-tosyl-3-oxatetracyclo[3.2.0.0^{2,7}.0^{4,6}]heptan-1-yl)aniline)

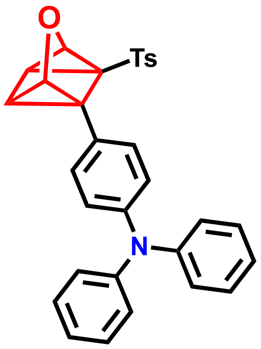

Chemical Formula: $C_{31}H_{25}NO_3S$
Molecular Weight: 491.60

Switching of **O-NBD2** was conducted according to general procedure D. NBD was dissolved in $CDCl_3$ and irradiated at 385 nm in 5 min steps. Quantitative conversion to O-QC2 was achieved 15 min in NMR scale (max. conc. 1.72 × 10^{-2} M). Prolonged irradiation led to the formation of the unidentified species **O-UnS2**. Higher concentration yielded a maximum conversion of 72% followed again by further rearrangement. In UV–vis scale (general procedure E), full isomerization to **O-QC2** was obtained after approx. 3 seconds.

Conversion: quant. (15 min, $CDCl_3$).

^{1}**H-NMR** (500 MHz, $CDCl_3$, -10 °C) δ_H [ppm]: 7.34-7.30 (m, 2H), 7.30-7.27 (m, 4H), 7.18-7.15 (m, 2H), 7.10-7.07 (m, 4H), 7.06-7.02 (m, 2H), 6.96-6.91 (m, 4H), 5.30 (dd, *J* = 4.1, 1.6 Hz, 1H), 4.55 (dd, *J* = 4.0, 1.6 Hz, 1H), 3.12-3.10 (m, 1H), 2.53-2.51 (m, 1H), 2.39 (s, 1H).

^{13}C-NMR (126 MHz, $CDCl_3$, -10 °C): (δ) [ppm] = 147.5, 144.4, 136.8, 131.3, 129.5, 129.4, 128.0, 124.3, 124.2, 123.4, 123.1, 70.0, 69.7, 48.2, 34.1, 27.7, 21.9, 20.4.

UV–vis: λ_{max} = 302 nm, $\lambda_{\pi\rightarrow\pi^*}$ = 229 nm. (leftover absorption can be found at λ = 377 nm)

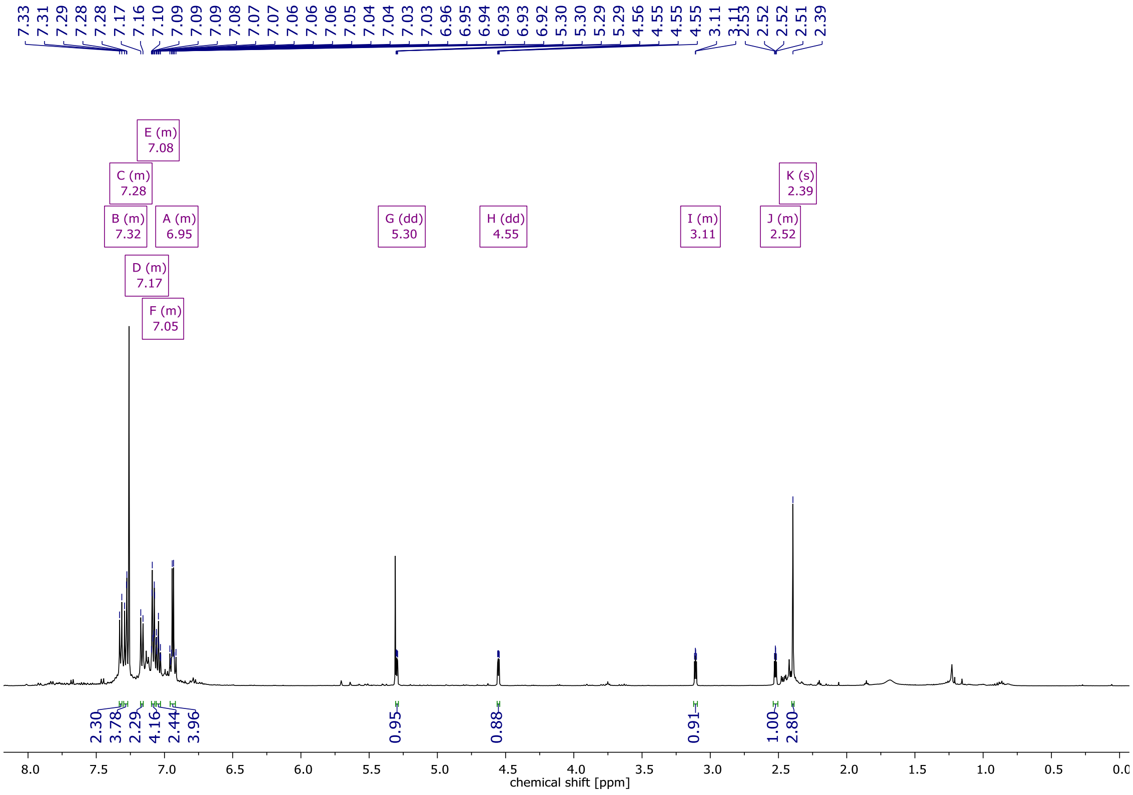

*Figure S42: ^{1}H-NMR spectrum of **O-QC2** measured in in $CDCl_3$ (500 MHz, -10 °C).*

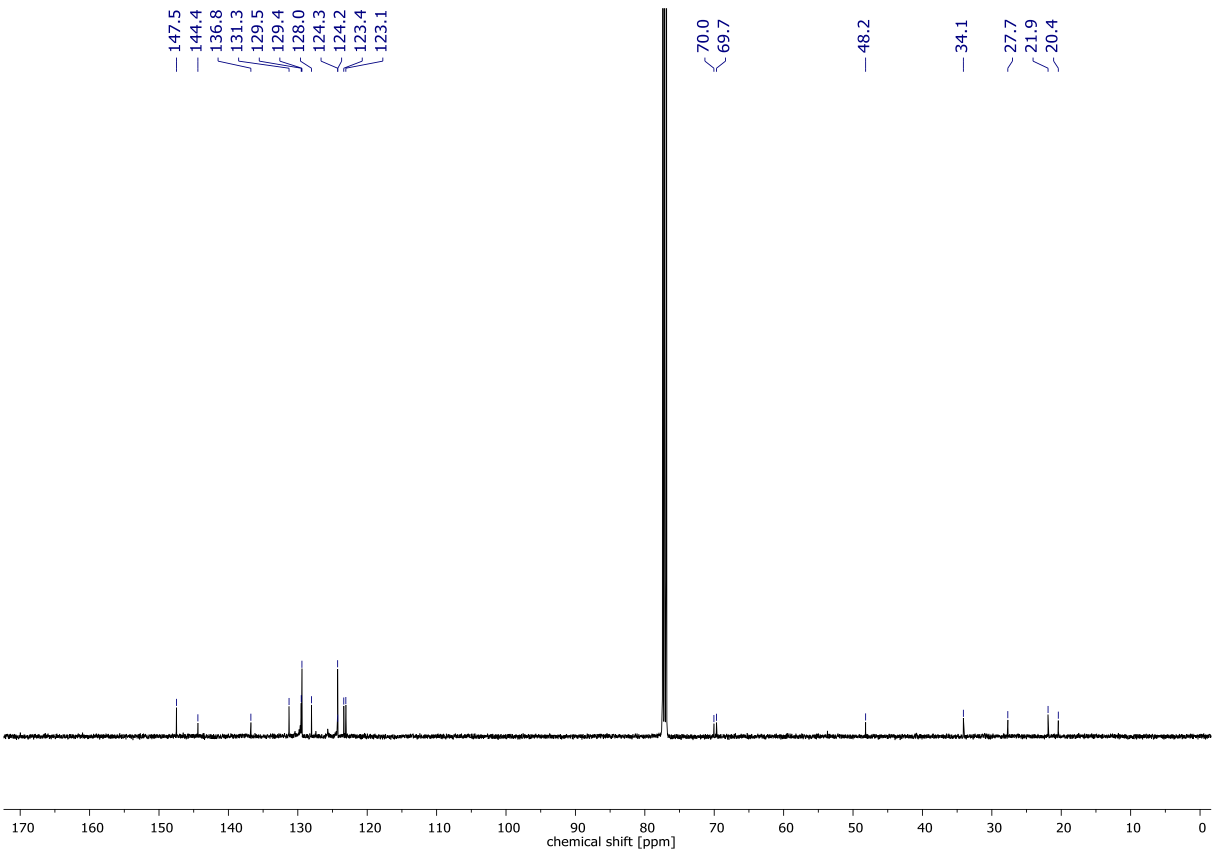

*Figure S43: ^{13}C-NMR spectrum of **O-QC2** measured in in $CDCl_3$ (125 MHz, -10 °C).*

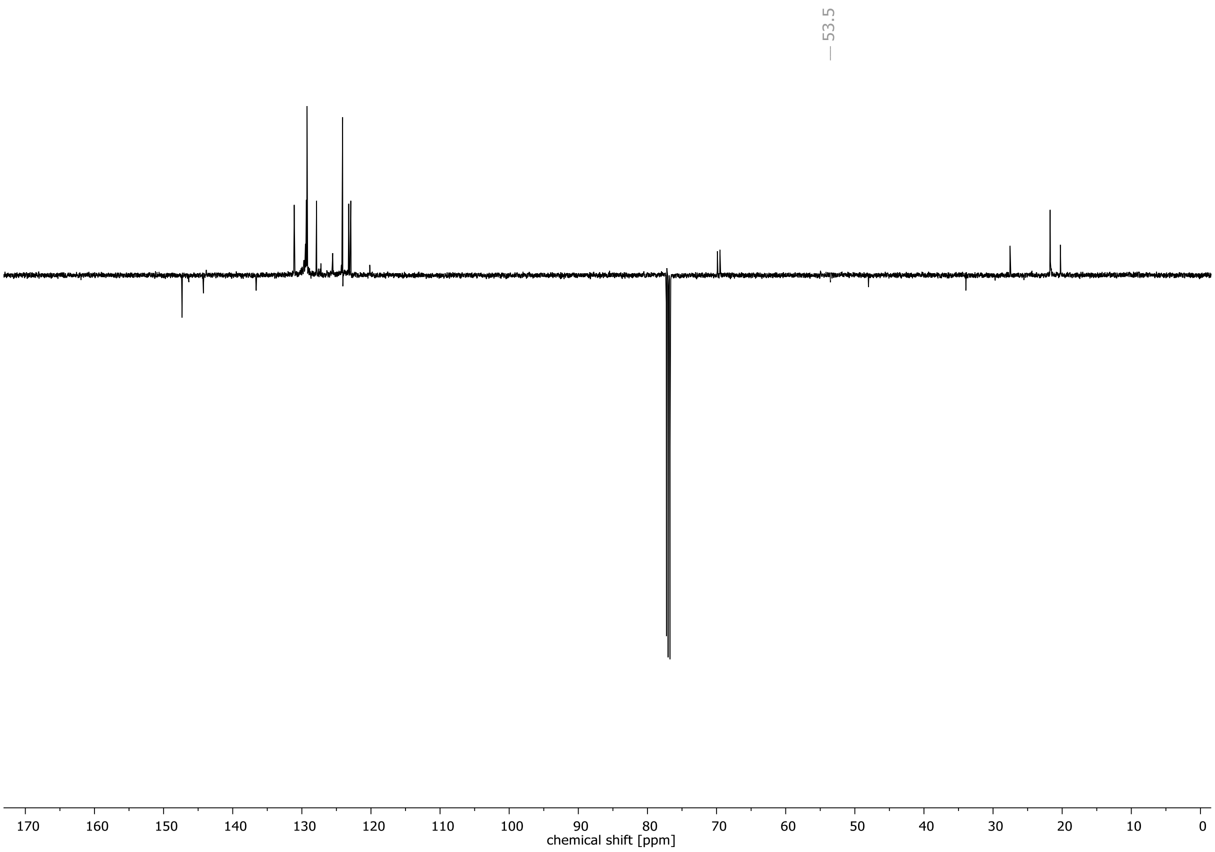

*Figure S44: DEPTq spectrum of **O-QC** measured in in $CDCl_3$ (125 MHz, -10 °C). The residual signal at 53.5 ppm corresponds to methylene chloride.*

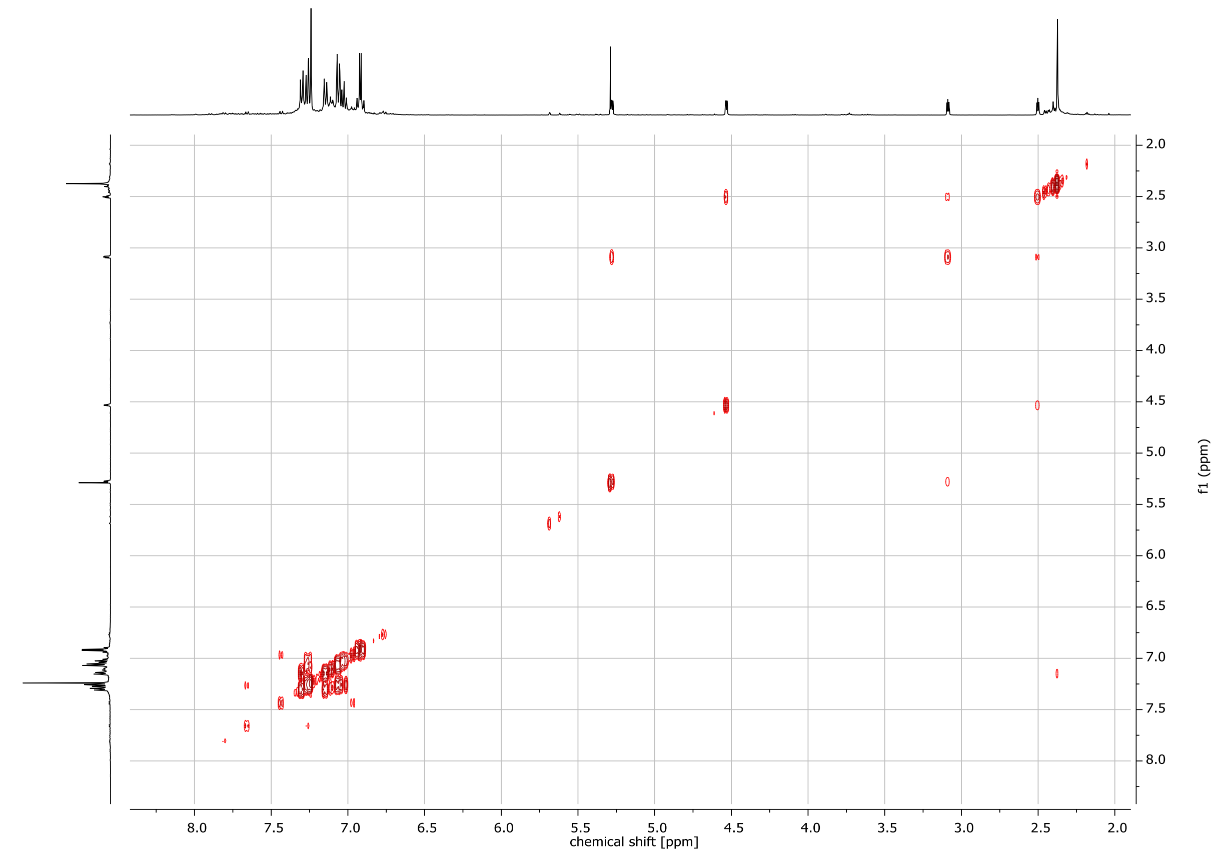

*Figure S45: COSY spectrum of **O-QC2** measured in in $CDCl_3$ (500 MHz, -10 °C).*

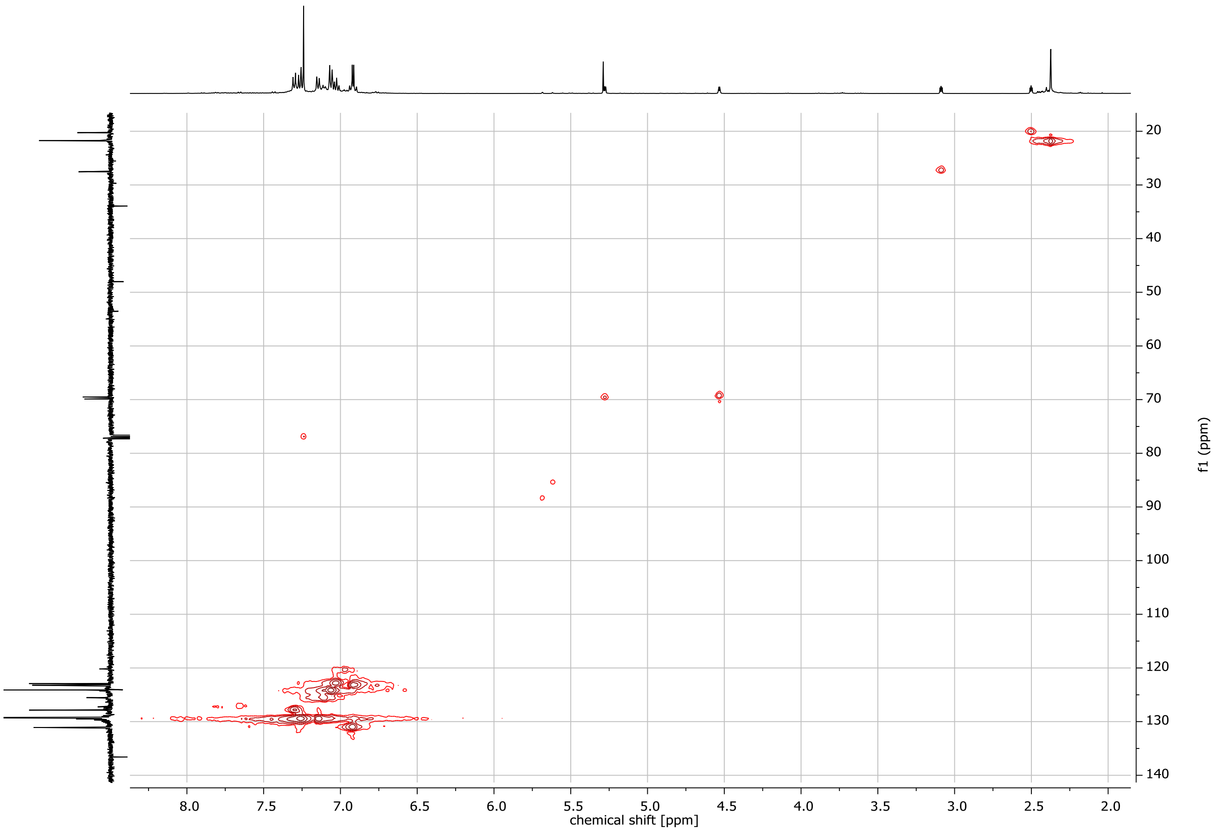

*Figure S46: HSQC spectrum of **O-QC2** measured in in $CDCl_3$ (500 MHz, -10 °C).*

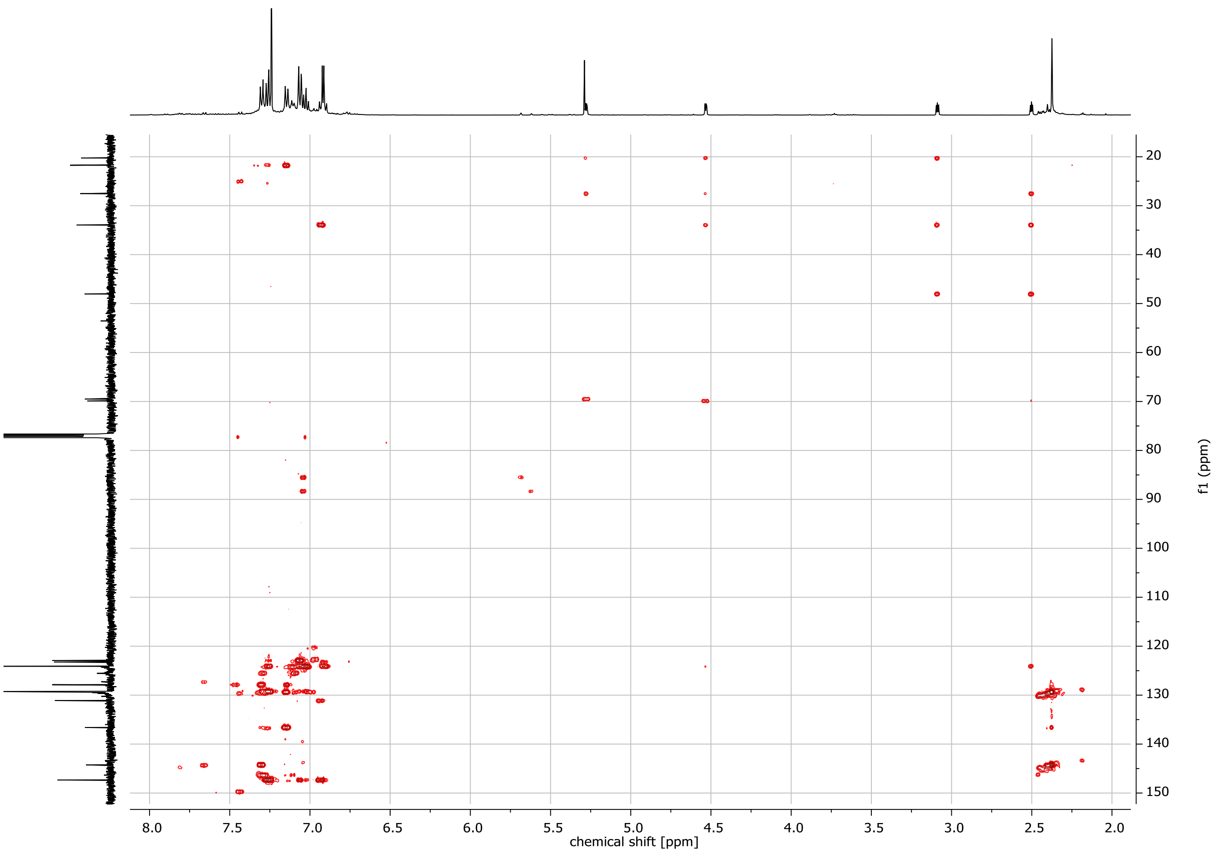

*Figure S47: HMBC spectrum of **O-QC2** measured in in $CDCl_3$ (500 MHz, -10 °C).*

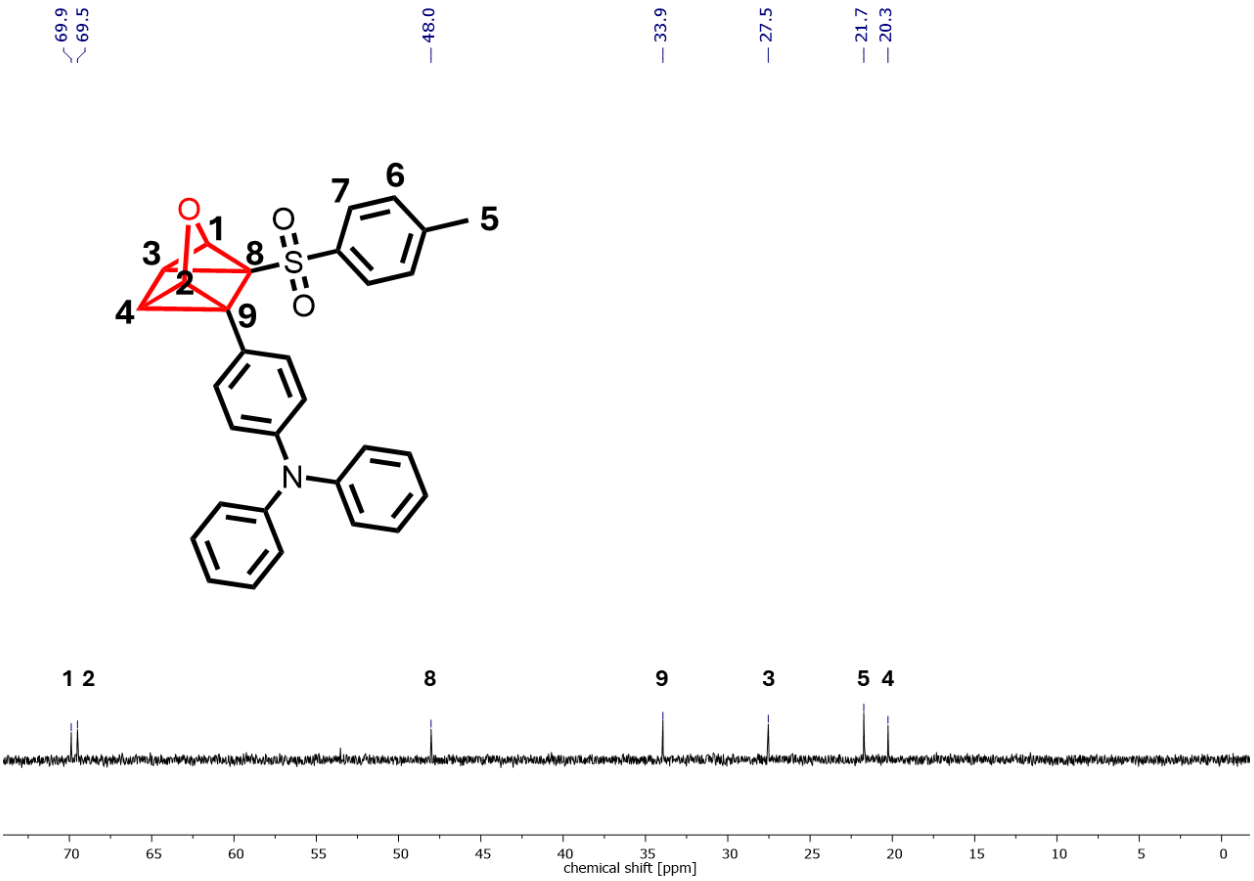

*Figure S48: Significant section of the ^{13}C NMR spectrum of **O-QC2** including assignment of the carbon scaffold based on 2D NMR spectroscopy.*

1.2.11 N-QC2 (*tert*-Butyl 1-(4-(diphenylamino)phenyl)-5-tosyl-3-azatetracyclo[3.2.0.0^{2,7}.0^{4,6}]heptane-3-carboxylate

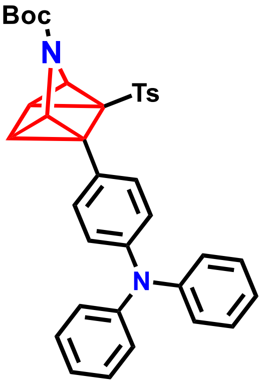

Chemical Formula: $C_{36}H_{34}N_2O_4S$
Molecular Weight: 590.74

Conversion of **N-NBD2** to **N-QC2** could not satisfy be proven. Similar findings as described for **N-NBD1** were found. Switching of **N-NBD2** was conducted according to general procedure B. **N-NBD2** (6.5 mg) was dissolved in $CDCl_3$ and irradiated at 385 nm. Quantitative conversion to a new unknown species **N-UnS2** was achieved after 20 min in NMR scale. In UV–vis scale (general procedure C), consumption of **N-NBD2** was finished after 20 seconds accompanied by slight indication of photodecomposition. Prolonged irradiation led to complete photodecomposition in both spectroscopic investigations. Since the addition of **POR** did not result in the regeneration of **N-NBD2**, formation of **N-QC2** is most likely excluded. The found spectroscopic features might indicate the formation an 2,5-dusubstituted azepine derivative[10] while confirmation of such structure was not possible.

Conversion: Formation of a rearranged species **N-UnS2** accompanied by photodecomposition.

^{1}H-NMR (400 MHz, $CDCl_3$, 25 °C) δ_H [ppm]: 7.73-7.63 (m, 2H), 7.46-7.39 (m, 2H), 7.33-7.28 (m, 4H), 7.27-7.26 (m, 1H), 7.26-7.24 (m, 1H), 7.15-7.08 (m, 6H), 7.01-6.93 (m, 4H), 5.51-5.35 (m, 2H), 2.41 (s, 3H), 1.40-1.31 (m, 9H).

λ_{max} = 298 nm, $\lambda_{\pi\rightarrow\pi^*}$ = 211 nm. (leftover absorption can be found at λ = 370 nm).

1.3 Switching Experiments

1.3.1 C-NBD1 to C-QC1

For interconversion studies of the precursor C-NBD1 to C-QC1 we would like to refer to literature [3].

1.3.2 O-NBD1 to O-QC1

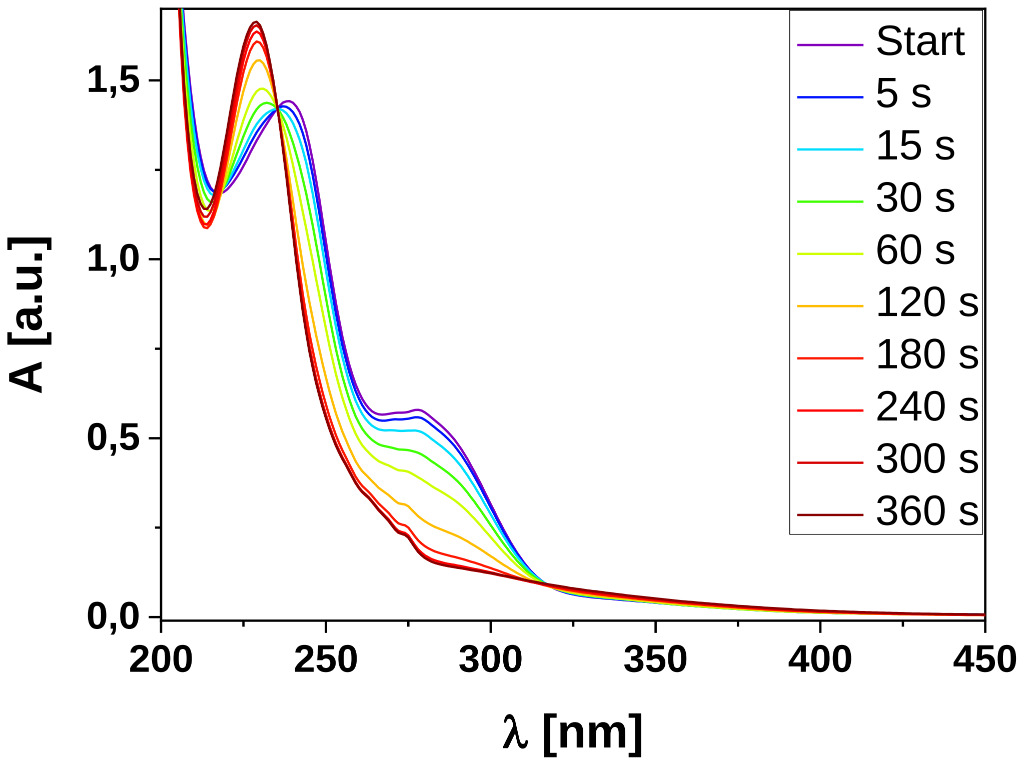

*Figure S49: UV–vis switching study of **O-NBD1** (1.29 × 10^{-4} M) measured in MeCN. For the irradiation a 275 nm LED was used. Prolonged irradiation led to slight photodecomposition. The same figure is also depicted in the main manuscript and here only shown for completeness.*

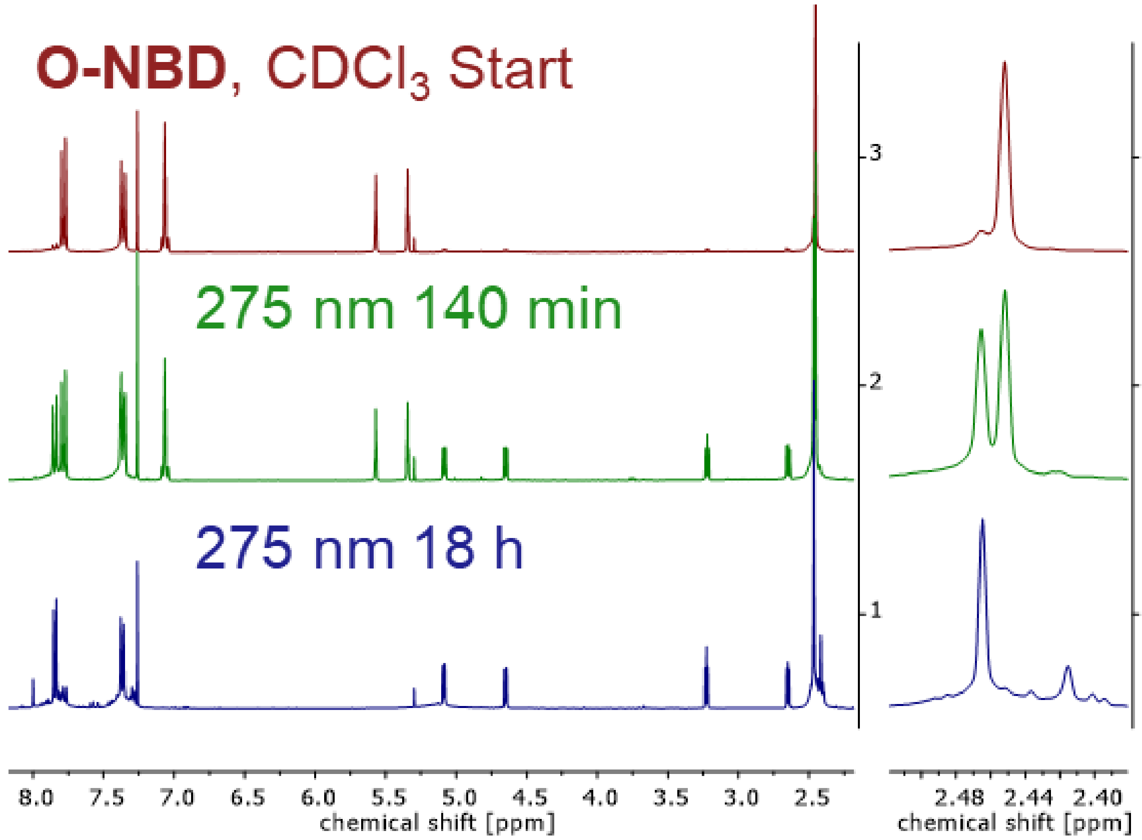

*Figure S50: NMR conversion study measured in $CDCl_3$. Top: **O-NBD1**, middle: mixture of **O-NBD1** and **O-QC1**; bottom: **O-QC1**. The same figure is also depicted in the main manuscript and here only shown for completeness.*

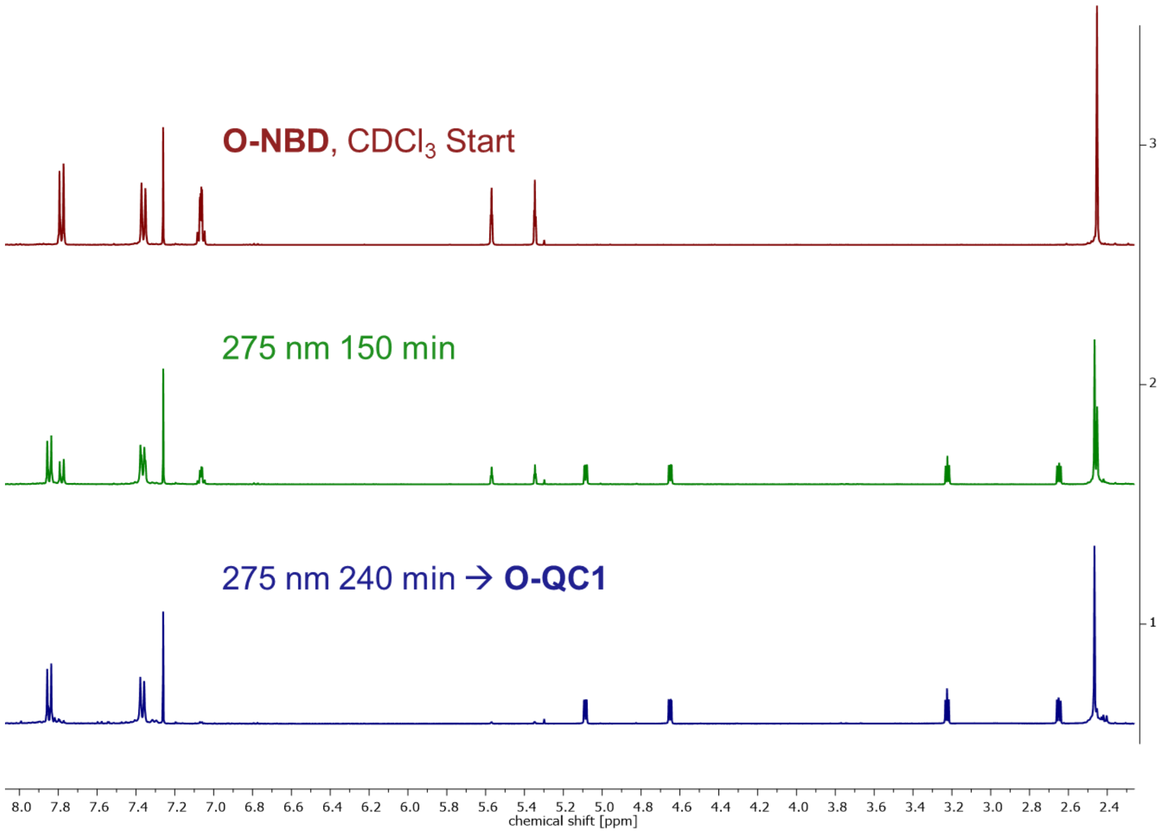

*Figure S51: NMR conversion study of **O-NBD1** measured in $CDCl_3$. Top: **O-NBD1**, middle: mixture of **O-NBD1** and **O-QC1** after 150 min irradiation at 275 nm; bottom: **O-QC1** after 240 min irradiation.*

1.3.3 N-NBD1 to N-QC1/N-UnS1

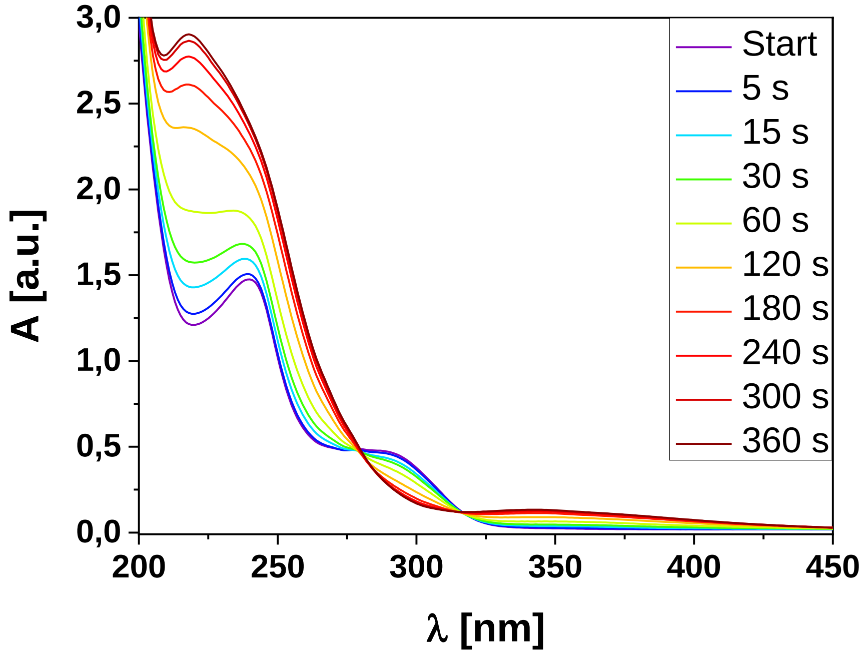

*Figure S 52: UV–vis switching study of **N-NBD1** (1.25 × 10⁻⁴ M) measured in MeCN. For the irradiation a 275 nm LED was used. Prolonged irradiation led to photodecomposition. The same figure is also depicted in the main manuscript and here only shown for completeness.*

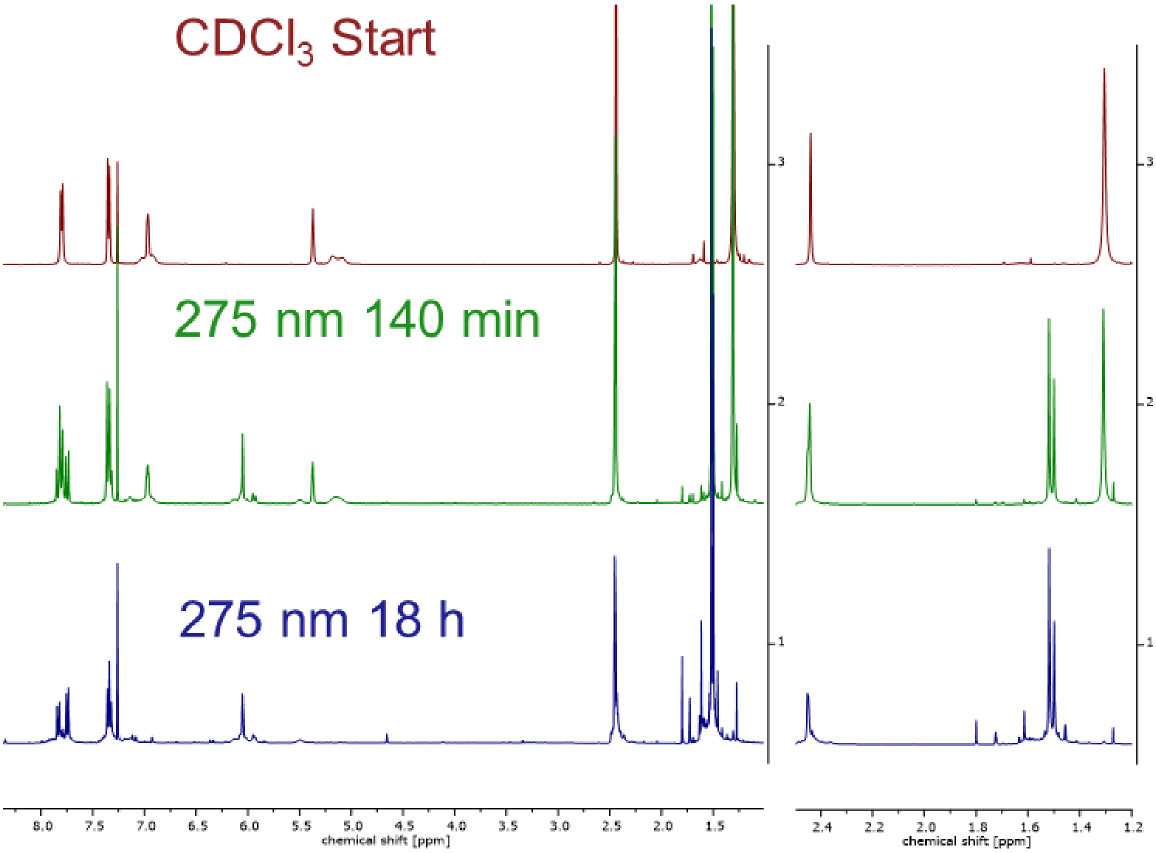

*Figure S53: NMR conversion study of **N-NBD1** measured in $CDCl_3$. Top: **N-NBD1**, middle: mixture of **N-NBD1** and **N-QC1**; bottom: **N-QC1**. The same figure is also depicted in the main manuscript and here only shown for completeness.*

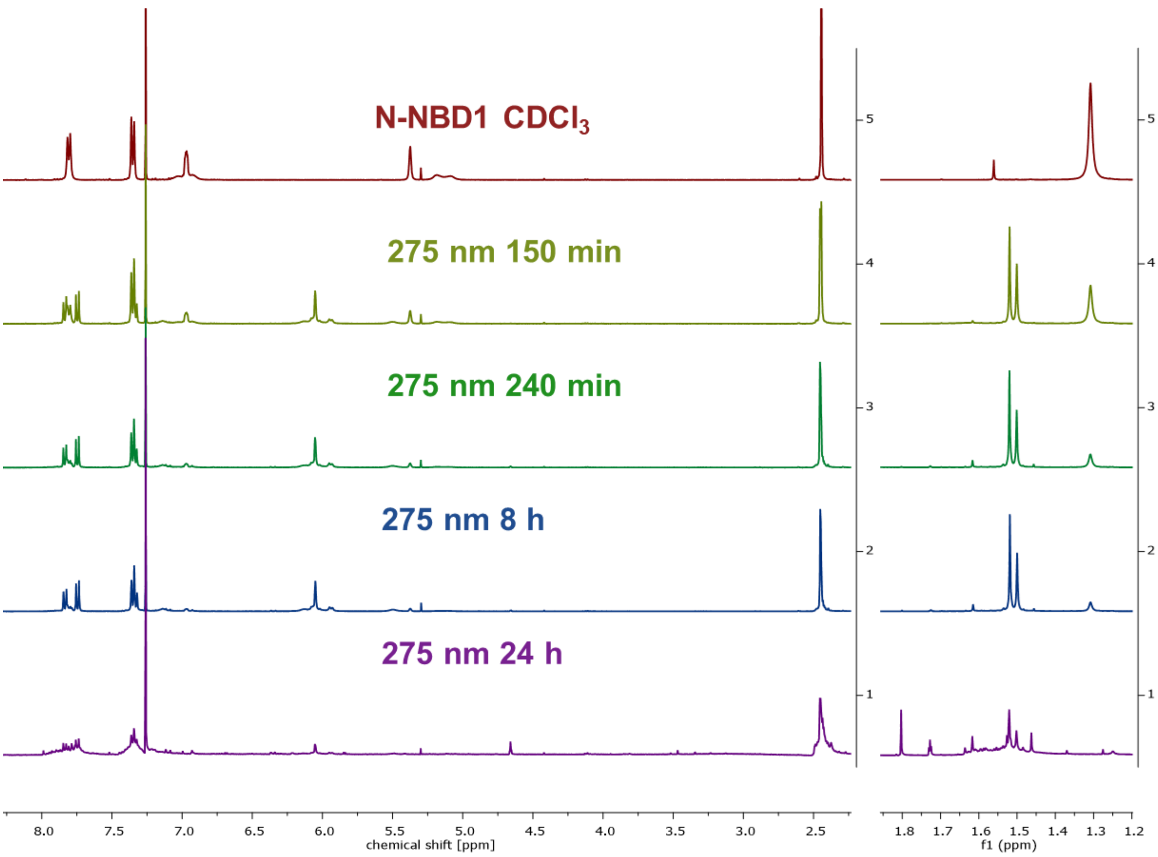

*Figure S54: Stepwise conversion study of **N-NBD1** measured in $CDCl_3$. For irradiation a 275 nm LED was used. After 24h, mainly photodecomposition was found.*

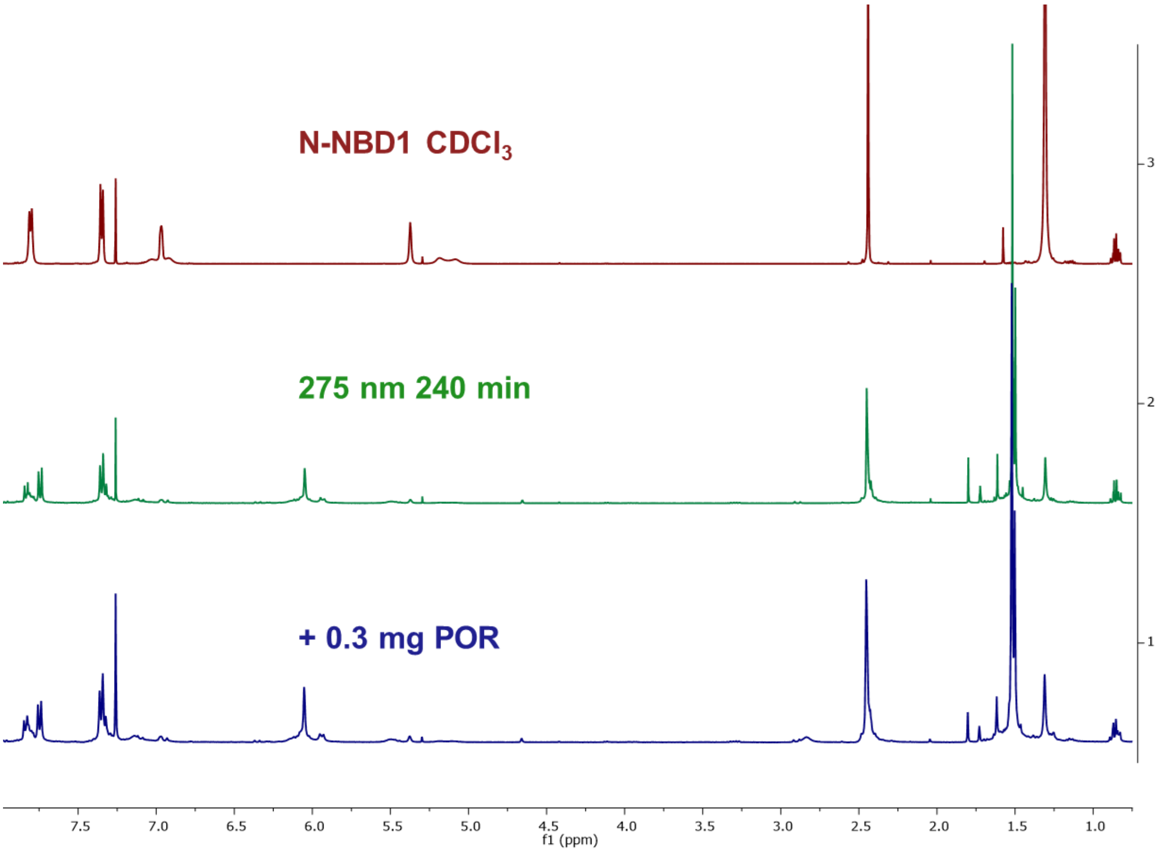

*Figure S55: NMR conversion study of **N-NBD1** measured in $CDCl_3$. Top: **N-NBD1**, middle: after irradiation of 240 min at 275 nm (**N-UnS1**); bottom: addition of POR did not result in the regain of **N-NBD1**.*

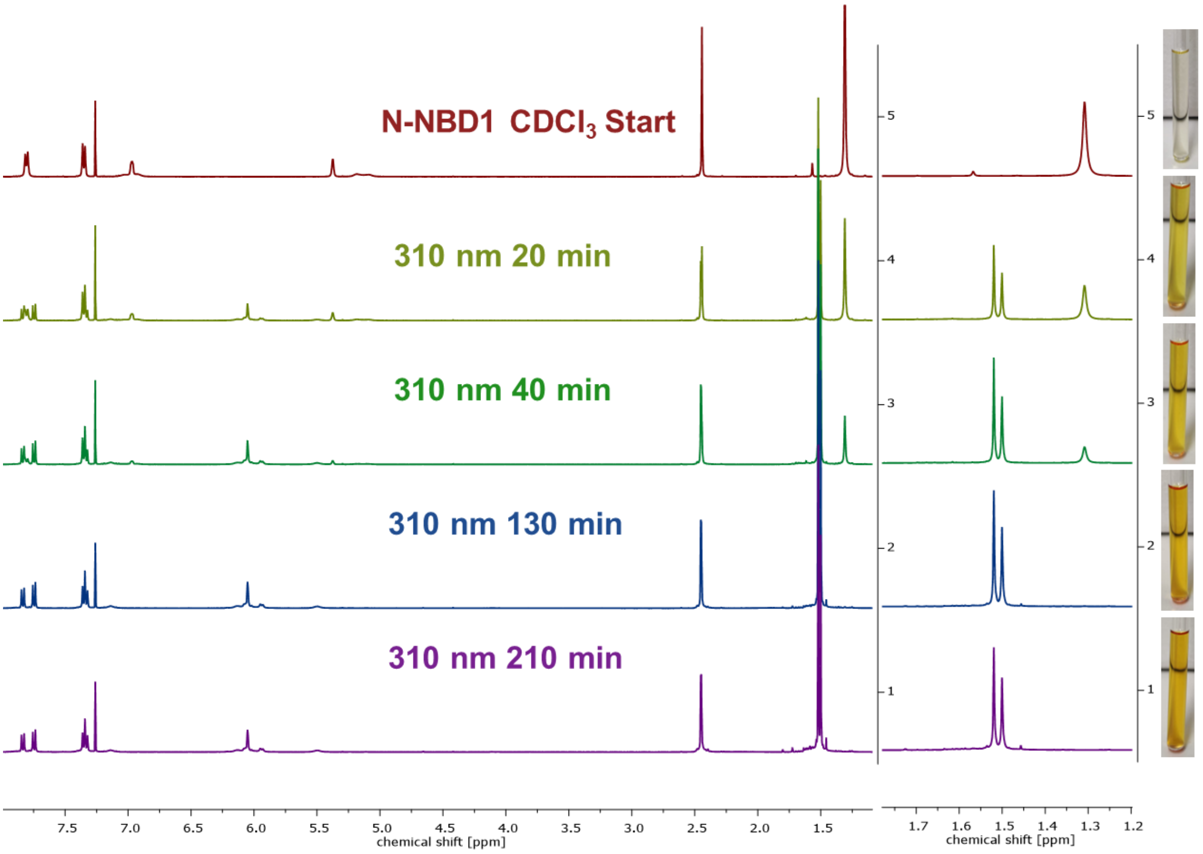

*Figure S56: Stepwise conversion study of **N-NBD1** (6.9 mg) measured in $CDCl_3$ (450 µL) to investigate the influence of the excitation wavelength. For irradiation a 310 nm LED block was used as described in the general information section. The same conversion as found for the 275nm irradiation experiment was observed with slightly less decomposition. Pictures of the corresponding NMR tube are shown on the right side.*

1.3.4 O-NBD2 to O-QC2 and further rearrangement to O-UnS2

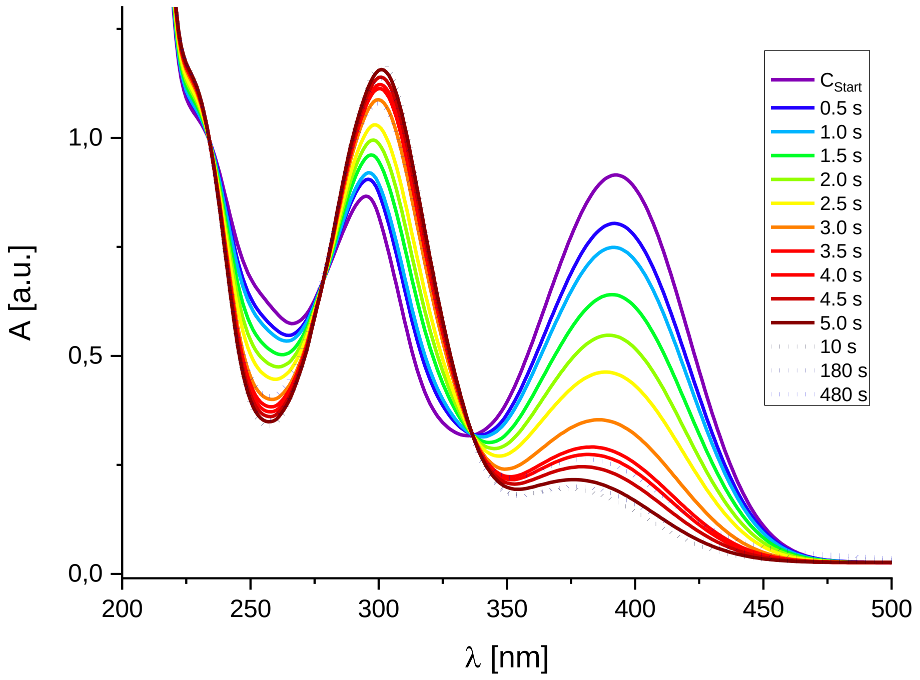

*Figure S57: UV–vis switching study of **O-NBD2** (5.97×10^{-5} M) measured in MeCN. For the irradiation a 385 nm LED was used. Prolonged irradiation led to further rearrangement or photodecomposition.*

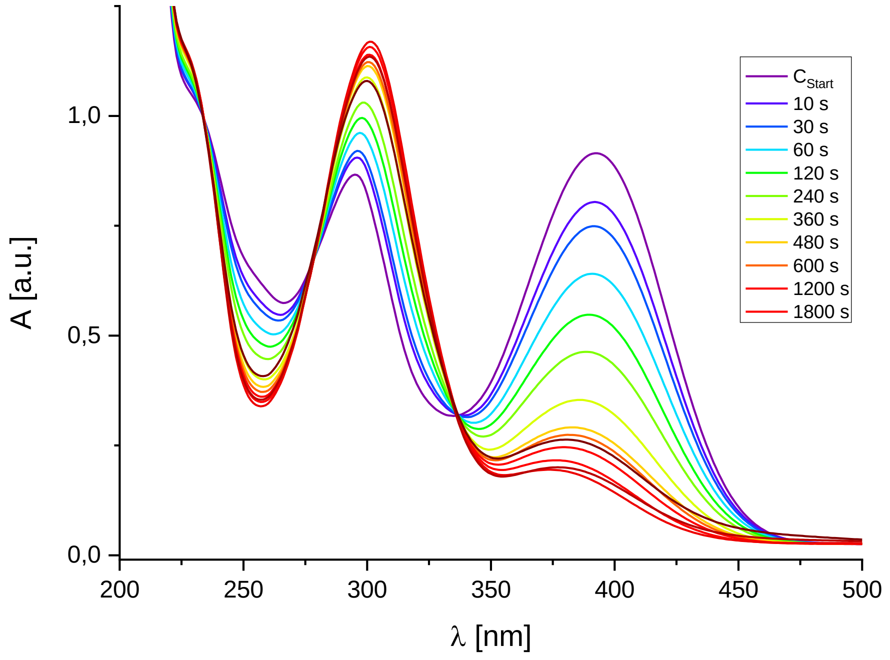

*Figure S58: UV–vis switching study of **O-NBD2** (5.97×10^{-5} M) measured in MeCN. For the irradiation a 385 nm LED was used. Prolonged irradiation led to further rearrangement or photodecomposition.*

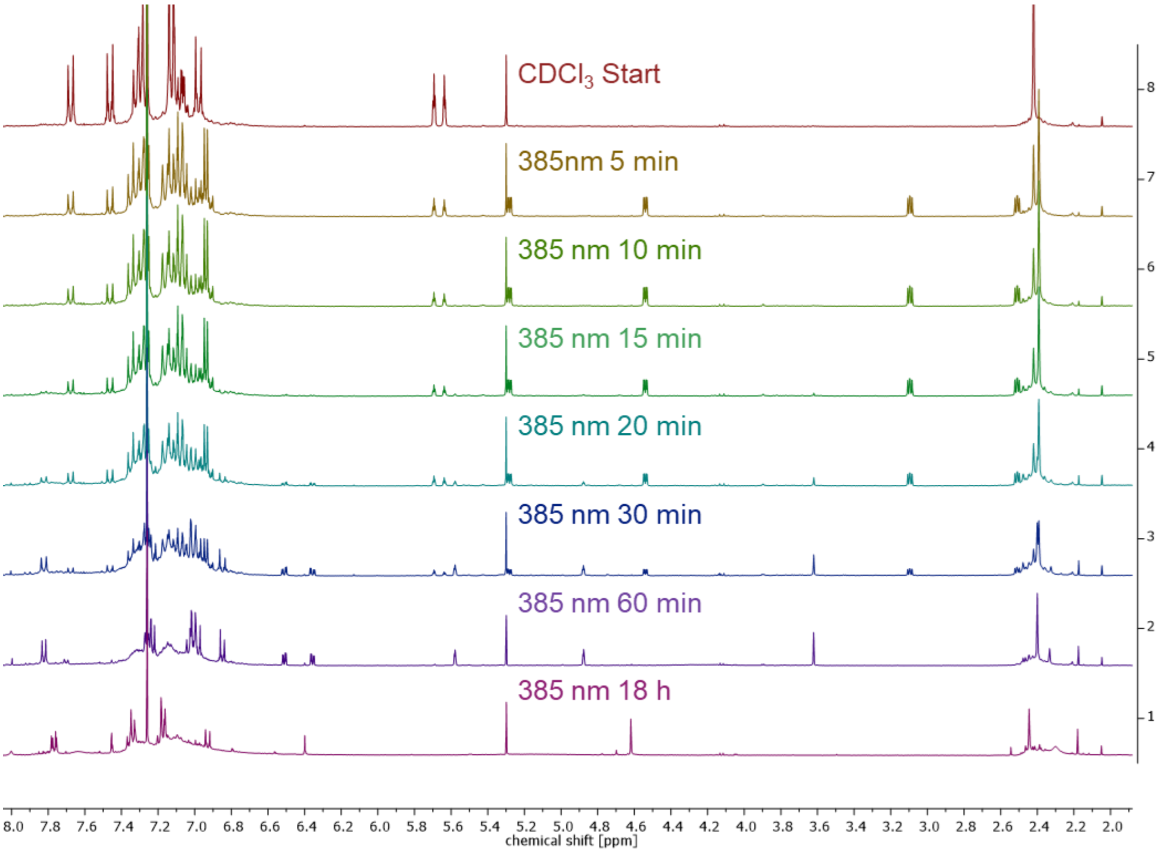

Figure S59: Rearrangement study of ***O-NBD2*** *to* ***O-QC2*** *using a 385 nm LED. 6.9 mg substance in 650 μL $CDCl_3$ were used. The same figure is also depicted in the main manuscript and here only shown for completeness.*

*Table S3: Species ratio determined during the irradiation experiment above. [a]**O-NBD2** signal was normalized to 1.0.*

Irradiation time [min]	Integral ratio[a] of O-UnS2/O-NBD2/O-QC2
5	0.1/1.0/1.86
10	0.12/1.0/2.33
15	0.23/1.0/2.36
20	0.58/1.0/1.96
30	1.86/1.0/1.74
60	7.86/1.0/0.93
1080	Photodecomposition

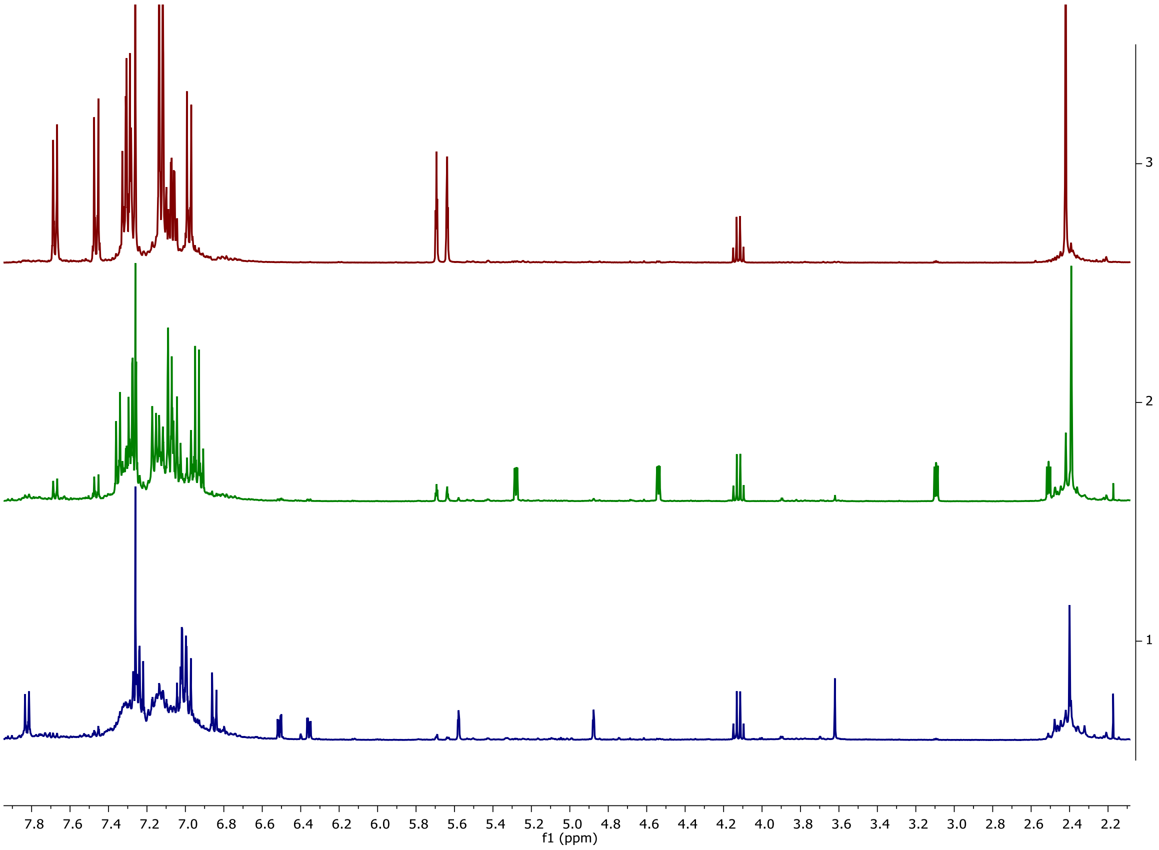

*Figure S60: Conversion of **O-NBD2** (red line) to **O-QC2** at higher concentrations (10.4 mg in 650 µL $CDCl_3$). After 30 min at 385 nm approx. 75% **O-QC2** (green line) was formed. 60 min irradiation at 385 nm led to complete formation of **O-UnS2**.*

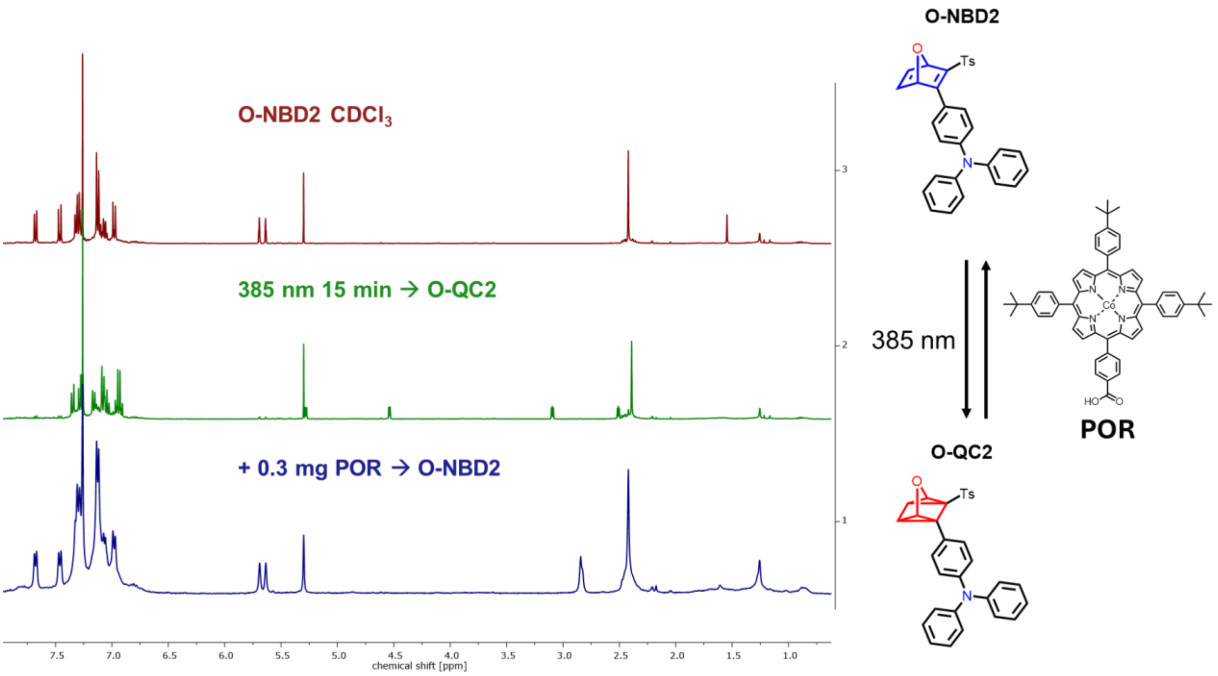

*Figure S61: NMR conversion study measured in $CDCl_3$. Top: **O-NBD2**, middle: **O-QC2** after irradiation at 385 nm for 15 min; bottom: addition of 0.3 mg mono-carboxylic acid-Co^{II}-Porphyrin*[7] *(**POR**) addition.*

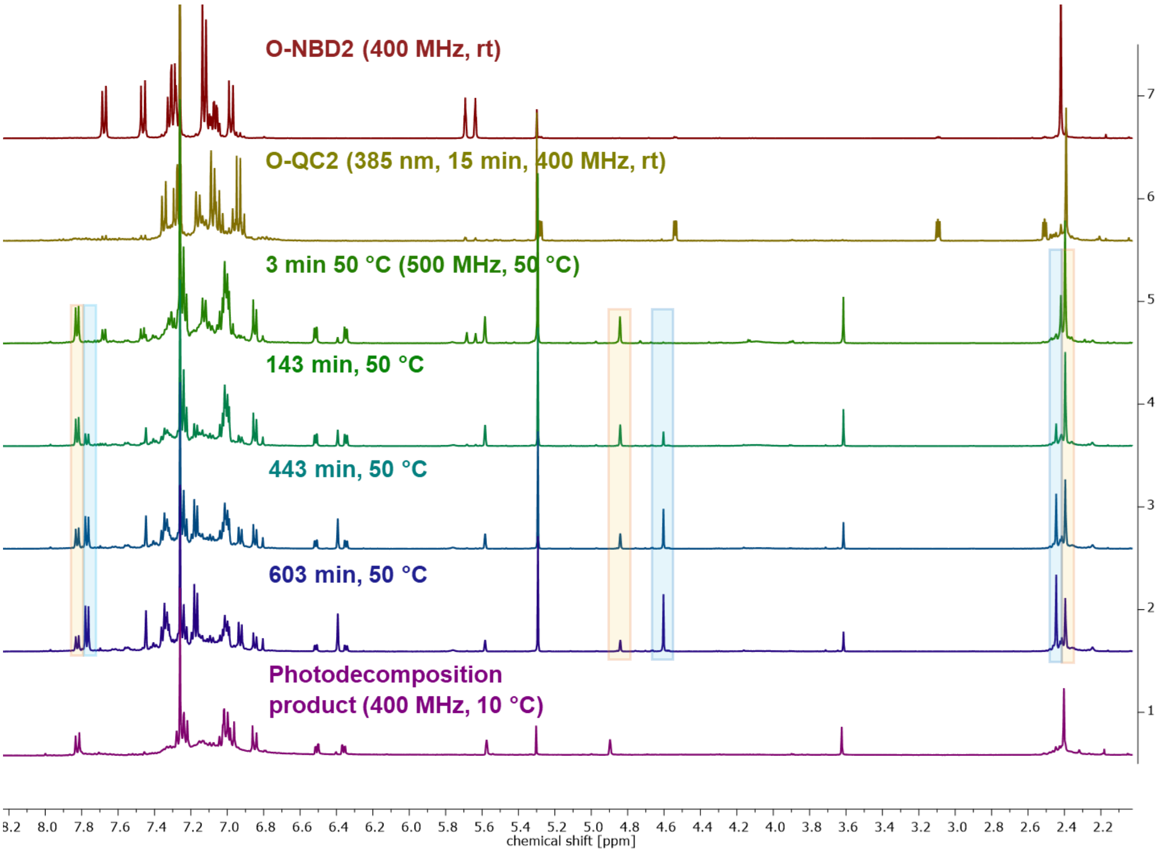

*Figure S62: Thermally induced conversion study of **O-QC2** back to **O-NBD2** monitored via NMR. All spectra were recorded in $CDCl_3$. **O-QC2** already further converted to **O-UnS2** and partially back to **O-NBD2**. Therefore, since no QC species was available, no back-conversion was achieved. However, upon heating, further conversion of **O-UnS2** (orange highlights), to another unidentified species was found (blue highlights). For comparison, the spectrum of **O-UnS2** is given at the bottom measured at 10 °C at 400 MHz reasoning in slightly different chemical shifts compared to the spectra above.*

1.3.5 N-NBD2 to N-QC2/N-UnS2

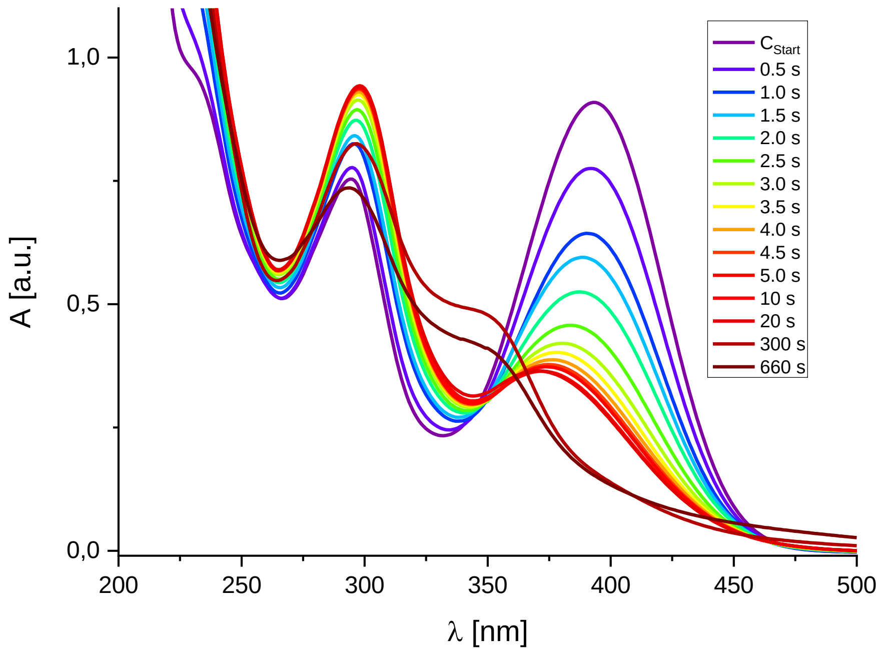

*Figure S63: UV–is switching study of **N-NBD2** (1.19 × 10⁻⁴ M) measured in MeCN. For the irradiation a 385 nm LED was used. Longer irradiation times than 20 s led to pronounced photodecomposition.*

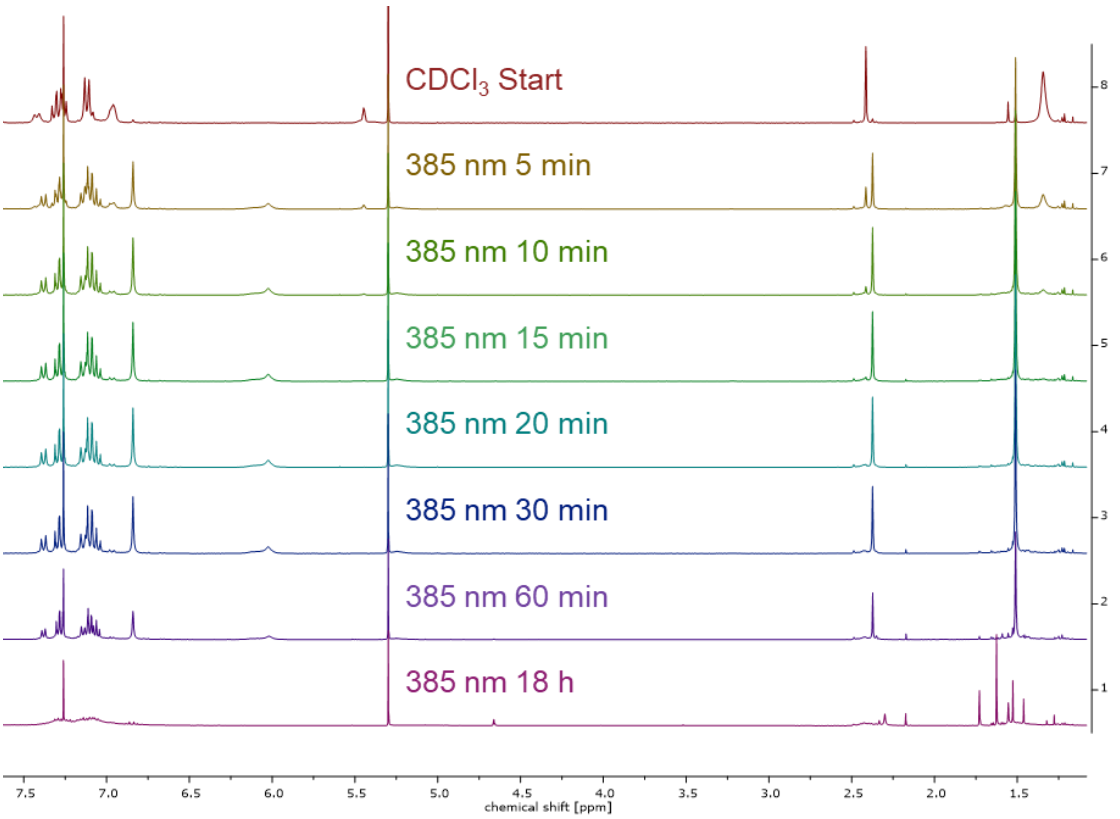

*Figure S64: Rearrangement study of **N-NBD2** to **N-QC2/N-UnS2** using a 385 nm LED. 6.9 mg substance in 650 µL $CDCl_3$ were used. The same figure is also depicted in the main manuscript and here only shown for completeness.*

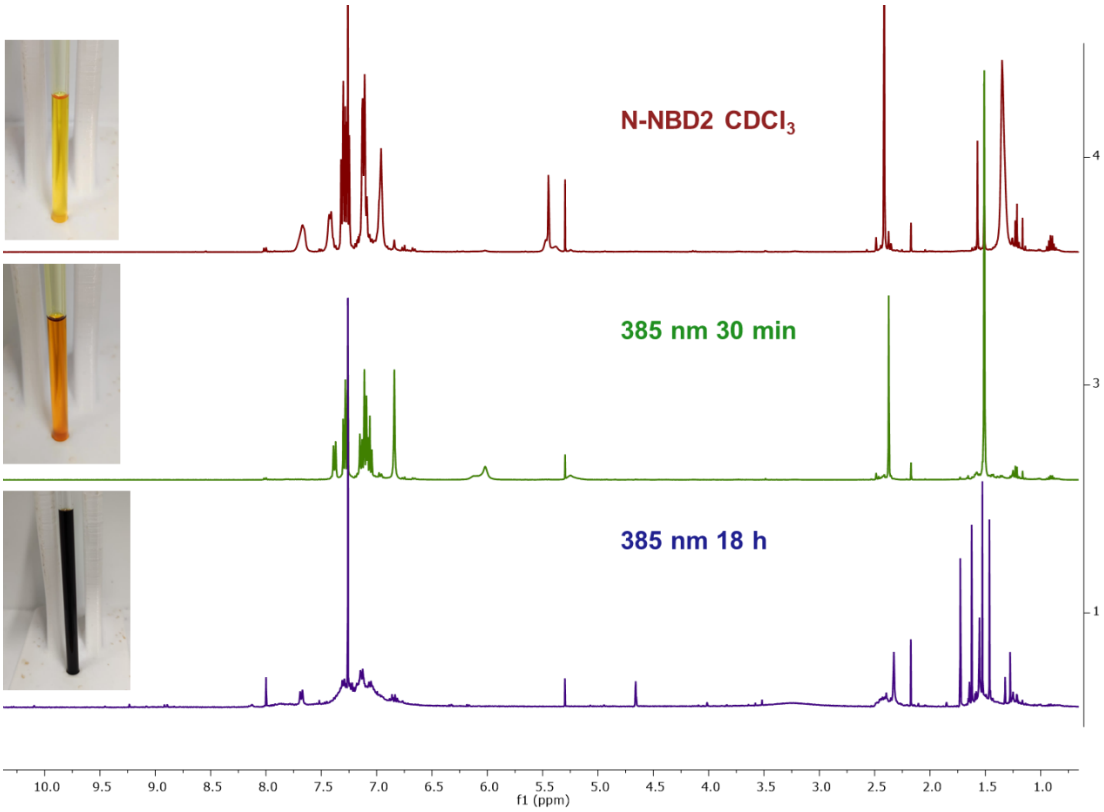

*Figure S65: Stepwise conversion study of **N-NBD2** measured in $CDCl_3$. For irradiation a 385 nm LED was used. After 18h, predominantly photodecomposition was found (yellow to black solution). Pictures of the corresponding NMR tube are shown on the left side.*

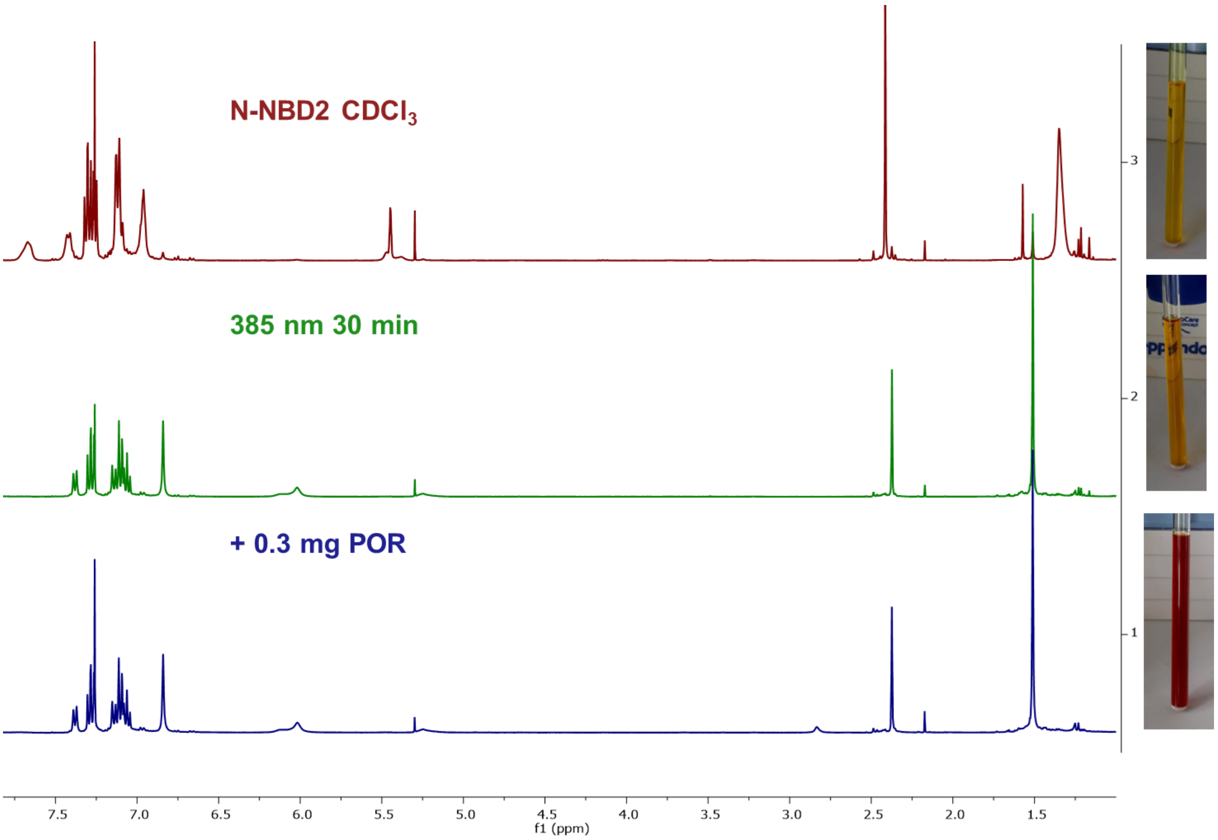

*Figure S66: NMR conversion study of **N-NBD2** measured in $CDCl_3$. Top: **N-NBD2**, middle: after irradiation of 30 min at 385 nm (**N-UnS2**); bottom: addition of POR did not result in the regain of **N-NBD2**. Pictures of the corresponding NMR tube are shown on the right side.*

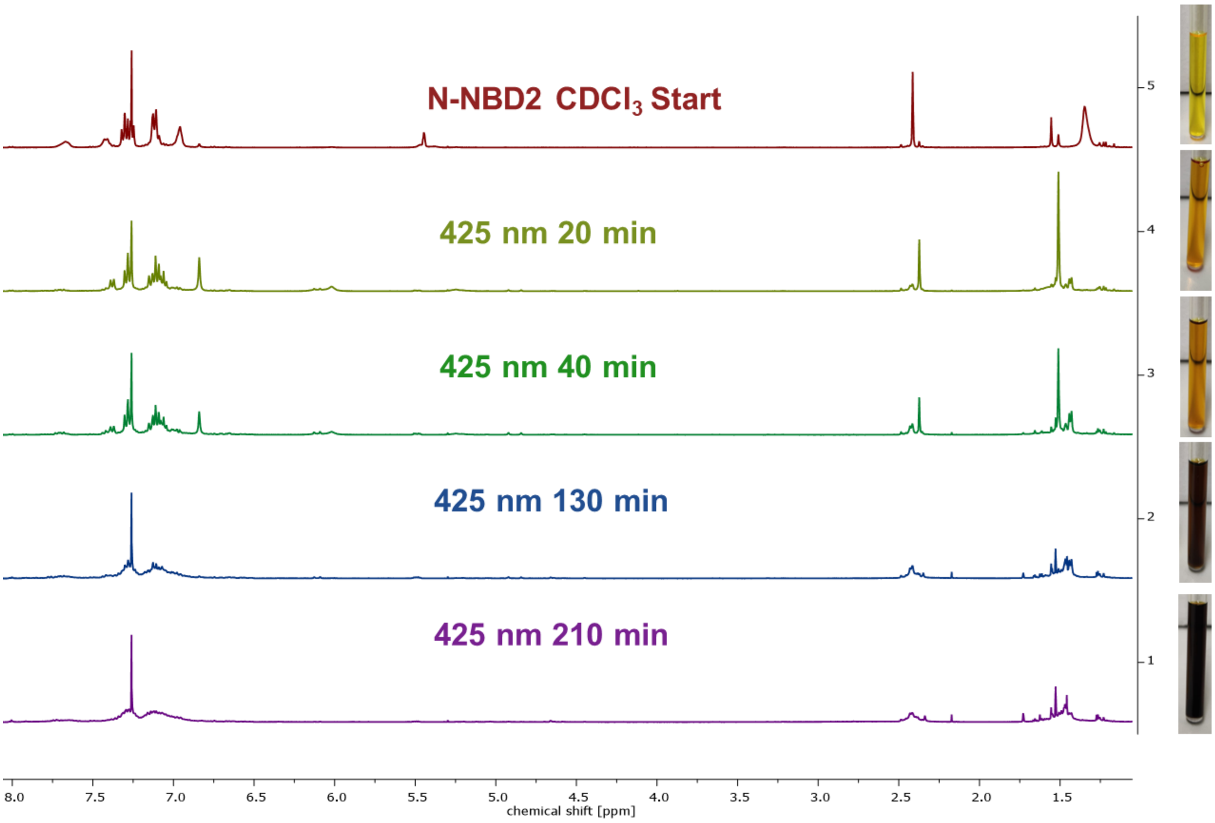

*Figure S67: Stepwise conversion study of **N-NBD2** (3.0 mg) measured in $CDCl_3$ (450 μL) to investigate the influence of the excitation wavelength. For irradiation a 425 nm LED block was used as described in the general information section. The same conversion as found for the 385 nm irradiation experiment was observed. Due to the significantly higher power of the 425 nm LED block, decomposition occurred earlier than before. Pictures of the corresponding NMR tube are shown on the ride side.*

1.4 References

1. Fulmer, G. R.; Miller, A. J. M.; Sherden, N. H.; Gottlieb, H. E.; Nudelman, A.; Stoltz, B. M.; Bercaw, J. E.; Goldberg, K. I. *Organometallics* **2010**, *29*, 2176–2179. doi:10.1021/om100106e

2. Cobas, C.; Sardina, J.; Dominguez, S. MestReNova. Mestrelab Research S.L. 2017

3. Krappmann, D.; Hirsch, A. *Chem. – A Eur. J.* **2024**, *30*, e202401391. doi:10.1002/CHEM.202401391

4. Quant, M.; Hillers-Bendtsen, A. E.; Ghasemi, S.; Erdelyi, M.; Wang, Z.; Muhammad, L. M.; Kann, N.; Mikkelsen, K. V.; Moth-Poulsen, K. *Chem. Sci.* **2022**, *13*, 834–841. doi:10.1039/D1SC05791J

5. Zhang, C.; Ballay, C. J.; Trudell, M. L. *J. Chem. Soc. - Perkin Trans. 1* **1999**, No. 6, 675–676. doi:10.1039/a900970a

6. Luchs, T.; Lorenz, P.; Hirsch, A. *ChemPhotoChem* **2020**, *4*, 52–58. doi:10.1002/cptc.201900194

7. Lorenz, P.; Luchs, T.; Hirsch, A. *Chem. – A Eur. J.* **2021**, *27*, 4993–5002. doi:10.1002/chem.202005427

8. Gil de Montes, E.; Istrate, A.; Navo, C. D.; Jiménez-Moreno, E.; Hoyt, E. A.; Corzana, F.; Robina, I.; Jiménez-Osés, G.; Moreno-Vargas, A. J.; Bernardes, G. J. L. *Angew. Chem., Int. Ed.* **2020**, *59*, 6196–6200. doi:10.1002/anie.201914529

9. Moreno-Clavijo, E.; Moreno-Vargas, A. J.; Kieffer, R.; Sigstam, T.; Carmona, A. T.; Robina, I. *Org. Lett.* **2011**, *13*, 6244–6247. doi:10.1021/ol2027009

10. Prinzbach, H.; Kaupp, G.; Fuchs, R.; Joyeux, M.; Kitzing, R.; Markert, J. *Chem. Ber.* **1973**, *106*, 3824–3849. doi:10.1002/CBER.19731061209
